# Design and Synthesis of Fluorescent Pilicides and Curlicides: Bioactive Tools to Study Bacterial Virulence Mechanisms

**DOI:** 10.1002/chem.201103936

**Published:** 2012-03-16

**Authors:** Erik Chorell–, Jerome S Pinkner, Christoffer Bengtsson, Sofie Edvinsson, Corinne K Cusumano, Erik Rosenbaum, Lennart B Å Johansson, Scott J Hultgren, Fredrik Almqvist

**Affiliations:** a Dr. E. Chorell-, C. Bengtsson, S. Edvinsson, Dr. E. Rosenbaum, Prof. Dr. L. B. Å Johansson, Prof. Dr. F. Almqvist Department of Chemistry Umeå University,90187 Umeå (Sweden), Fax: (+46) 907-867-655; b J. S. Pinkner, Dr. C. K. Cusumano, Prof. Dr. S. J. Hultgren Department of Molecular Microbiology Washington University School of Medicine,St. Louis, MO 63110 (USA); c Dr. E. Chorell-, J. S. Pinkner, Dr. C. K. Cusumano, Prof. Dr. S. J. Hultgren Center for Women's Infectious Disease Research, St. Louis, MO 63110 (USA); d Prof. Dr. F. Almqvist Umeå Centre for Microbial Research, Umeå University, 90187 Umeå (Sweden)

**Keywords:** antivirulence, biological activity, coumarin, fluorescence, structure–activity relationships

## Abstract

Pilicides and curlicides are compounds that block the formation of the virulence factors pili and curli, respectively. To facilitate studies of the interaction between these compounds and the pili and curli assembly systems, fluorescent pilicides and curlicides have been synthesized. This was achieved by using a strategy based on structure–activity knowledge, in which key pilicide and curlicide substituents on the ring-fused dihydrothiazolo 2-pyridone central fragment were replaced by fluorophores. Several of the resulting fluorescent compounds had improved activities as measured in pili- and curli-dependent biofilm assays. We created fluorescent pilicides and curlicides by introducing coumarin and 4,4-difluoro-4-bora-3a,4a-diaza-*s*-indacene (BODIPY) fluorophores at two positions on the peptidomimetic pilicide and curlicide central fragment. Fluorescence images of the uropathogenic *Escherichia coli* (UPEC) strain UTI89 grown in the presence of these compounds shows that the compounds are strongly associated with the bacteria with a heterogeneous distribution.

## Introduction


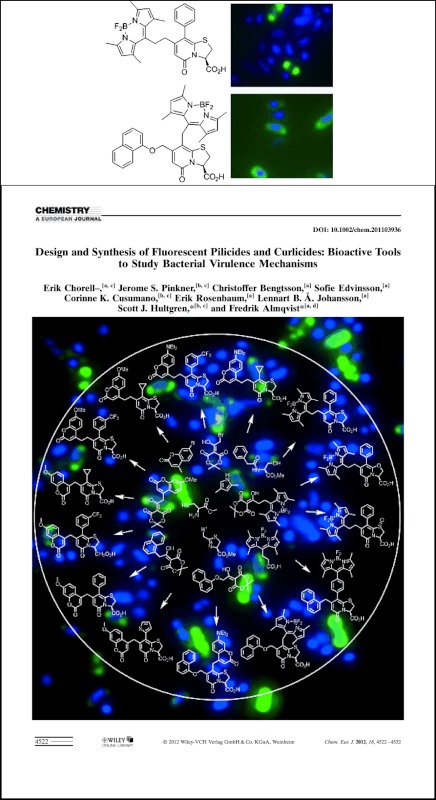


Multidrug resistant (MDR) bacterial strains present a growing global health problem. As a consequence, the search for new antibacterial agents and new methods to deal with bacterial resistance is urgent.^1^ Toward this end, understanding the details of the uropathogenic *E. coli* (UPEC) pathogenic cascade is revealing ways to target critical pathways to develop anti-virulence therapeutics. We have discovered that type-1 pili, an adhesive pili assembled by the chaperone/usher pathway (CUP), play an essential role in invasion of bladder cells and in the formation of biofilm-like intracellular bacterial communities (IBCs) that protect bacteria from host defenses and antibiotics.^2^–^5^ Further, CUP pili play a critical role in biofilm formation, mediating not only interactions with host tissue, but also colonization of catheters and other surfaces in nosocomial settings. Also, an amyloid fiber called curli is critical in UPEC biofilm formation and the molecular machine that mediates curli assembly has been dissected.^6^–^8^ Our understanding of the structural basis of CUP pili biogenesis has led to the design of pilicides that bind to the chaperone and block critical functions thus preventing pilus assembly.^9^–^11^ Numerous (>100) CUP systems are now known to be encoded in gram-negative genomes^12^ but little is known about their function. A typical *E. coli* genome encodes approximately 10 such systems.^13^ Further detailed studies of the function and regulation of CUP pili and other extracellular fibers are an important route to understanding the bacterial adaptation and survival strategies that may be particularly relevant to human infections and providing targets for the development of new therapeutics. Here we describe the development of new compounds that will serve as a strong foundation to support investigation of novel anti-fiber therapeutics targeting critical assembly and adhesion functions of fibers required for the determination of tropism and the organization of bacterial communities during infection.

**Figure 1:**
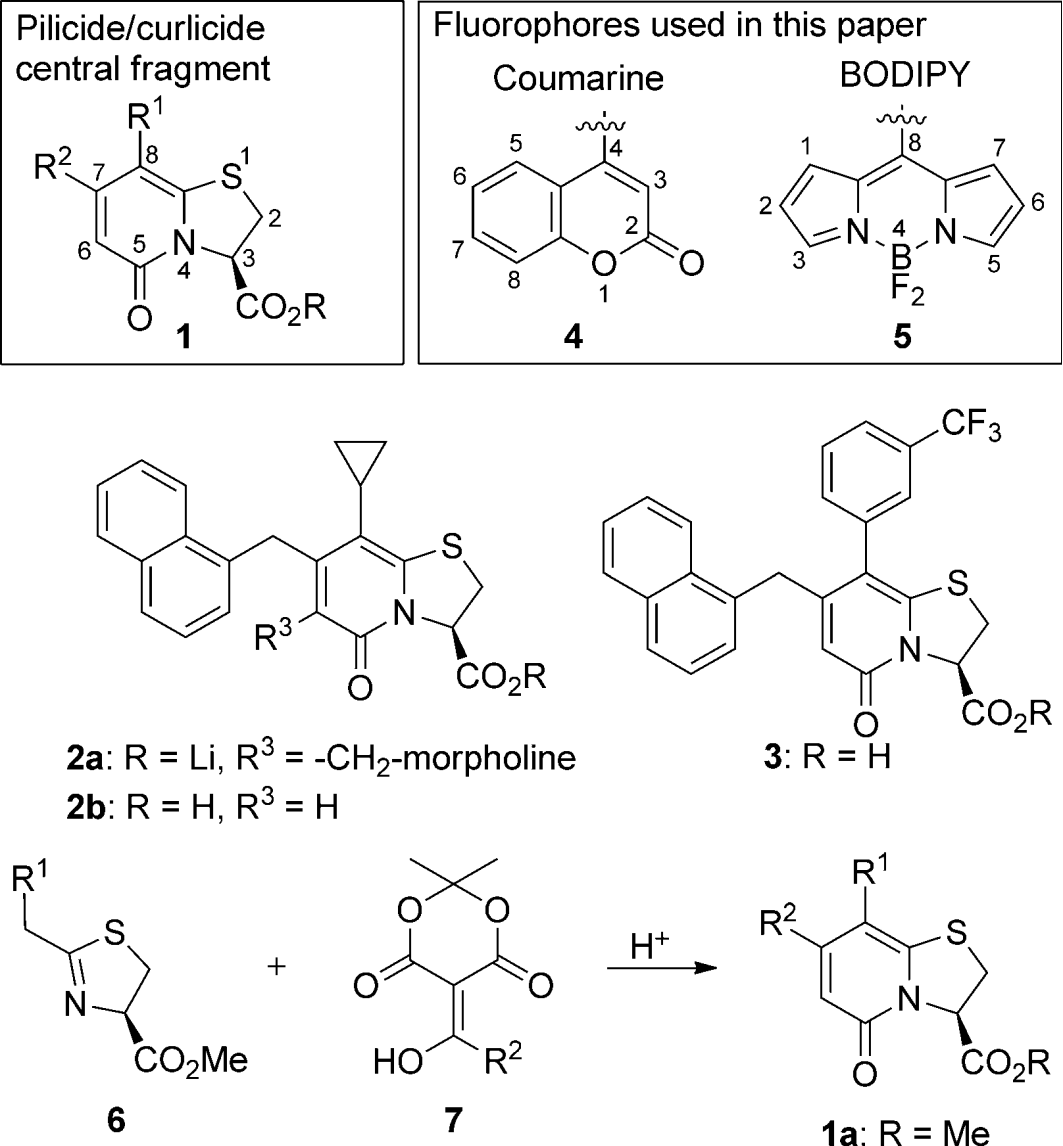
The dihydrothiazolo ring-fused 2-pyridone central fragment 1 (R=H or Li for biological activity), pilicide (2 a,b), curlicide (3), coumarin fluorophore 4, BODIPY fluorophore 5. Synthesis of the thiazolo ring-fused 2-pyridone central fragment (1 a) is performed by using 2-thiazolines (6) and Meldrum’s acid derivatives (7).

Ring-fused 2-pyridones are peptidomimetics that can target protein–protein interactions in macromolecular assembly. We have previously shown that ring-fused dihydrothiazolo 2-pyridones (**1**) provide an excellent central fragment for design and synthesis of compounds that block the formation of pili and curli.^10^, ^11^, ^14^ Pilicides (**2 a**,**b**) are compounds that block pilus biogenesis (exemplified in UPEC), whereas curlicides (such as **3**) prevent curli fiber biogenesis. Development of traceable pilicides and curlicides could potentially be obtained using a biomolecular labeling strategy, for example, by radiolabeling or by the introduction of a fluorescent label. In the latter case, a fluorescent probe is usually attached by using a linker to the biomolecule to avoid any interference with the biomolecular interactions. The low molecular weight of ligands such as the pilicides **2 a**,**b** and curlicide **3**, implies that this technique could change the overall molecular composition to a great extent and thereby potentially reduce the bioactivity of these compounds. An alternative approach would be to replace key substituents for bioactivity by a fluorophore. To increase the likelihood of succeeding by using this approach, and thus both retain the biological effect and gain fluorescent properties, the structure–activity knowledge on the central fragment could be used for both the choice of fluorophore and its positioning on the central fragment. One potential problem with this method is a higher probability for fluorescent quenching of the fluorophore due to its close proximity to the bioactive central fragment. Even so, exchange of one of the substituents on the peptidomimetic central fragment **1** to a fluorophore could render attractive compounds for uptake/distribution studies, development of competition-based assays, Förster resonance energy transfer (FRET) studies on binding interactions, and to specifically image conserved pili and curli assembly machineries in bacterial populations. Initial studies of the structure–activity relationships on the ring-fused dihydrothiazolo 2-pyridone central fragment have shown that the C7 and C8 positions are highly important for bioactivity and should preferably carry larger lipophilic substituents.^10^, ^11^, ^15^–^17^ Consequently, we have in the present study exchanged the substituents in the C7 and C8 positions on the central fragment **1** with coumarin (**4**) or BODIPY (**5**) fluorophores (Figure 1). The use of these particular fluorophores could be justified by their good (compound **4**) to high (compound **5**) quantum yields, absorption/emission wavelengths, lipophilicity, lack of net ionic charge, photostability, different emission colors and relatively small size.^18^–^20^

**Figure 2:**
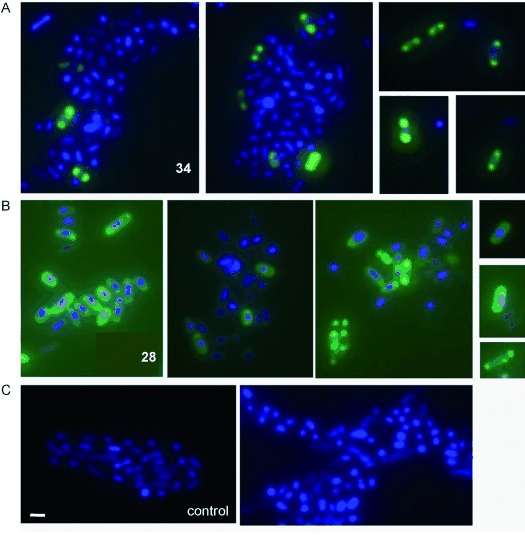
The UPEC strain UTI89 grown for 24 h with and without 100 μM of dual pilicide-curlicide **34** and **28**. Blue is DAPI-nuclear stain, green is the compound. A) Bacteria grown in the presence of 100 μM of compound **34**. B) Bacteria grown in the presence of 100 μM of compound **28**. C) Bacteria grown without any added compound (scale bar is 2 μm).

The synthesis of the ring-fused dihydrothiazolo 2-pyridone central fragment is based upon an acyl-ketene imine cyclocondensation between Δ^2^-thiazolines **6** and acyl Meldrum’s acid derivatives **7** (Figure 1).^21^ Carboxylic acids can be used as starting materials in the synthesis of both **6** and **7** and carboxylic acid functionalized fluorophores have therefore been used as building blocks for further incorporation in the C7 and C8 positions on the central fragment. In addition to this approach, coumarins could also be introduced directly on a bromomethyl-substituted central fragment using deprotonated 4-methyl coumarins. In total, 14 new fluorophore-substituted derivatives of the central fragment **1** have been synthesized and the photophysical measurements of these compounds revealed compounds with high quantum yields. In addition, biological evaluation of these compounds as pilicides and curlicides showed a great biological effect of several compounds, with some being both potent inhibitors of pili- and curli-dependent biofilm formation and having fluorescent properties. Finally, treatment of the UPEC strain UTI89 with the compounds under pili producing conditions shows that the compounds are associated to the bacteria and seem to discriminate between different bacteria in a population.

## Results and Discussion

**Scheme 1:**
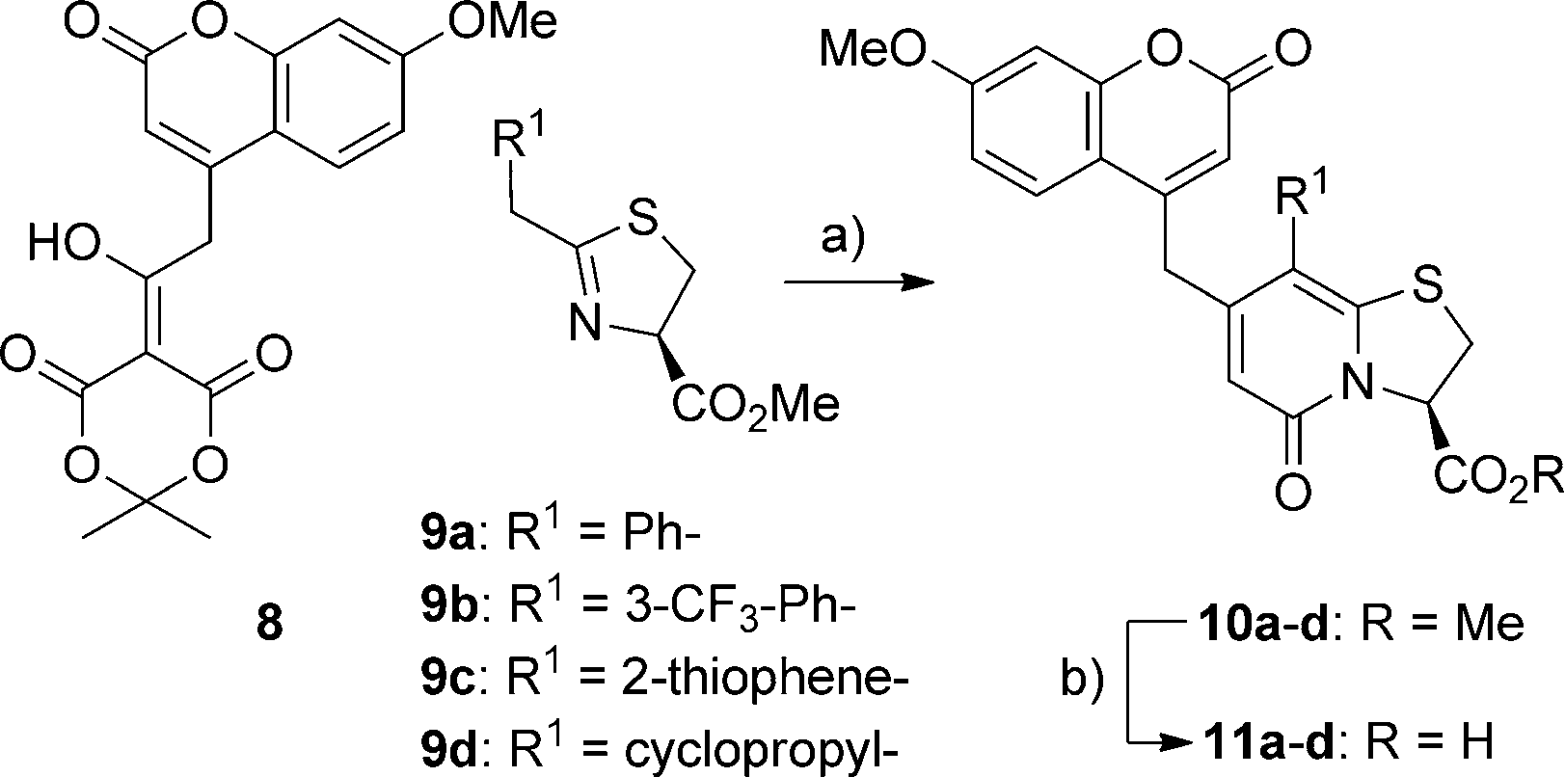
a) TFA, dichloroethane, microwave irradiation (MWI): 120 °C, 140 s (**10 a**–**d**: 77 %, 86 %, 77 and 68 %, respectively); b) i) 0.1 M aq LiOH, THF/MeOH (4:1); ii) AcOH (**11 a**–**d**: 66, 58, 53 and 66 %, respectively).

Coumarins substituted with electron-donating groups in position 7 such as 7-methoxy and 7-diethylamino coumarins are frequently used fluorophores (Figure 1). The linker to the pilicide/curlicide central fragment was preferred through the 4 position on the coumarins to resemble the geometry of the C7 naphthyl substituent in **2** and **3**. Consequently, the 7-methoxy coumarin-4-yl acetic acid was first coupled to its corresponding acyl Meldrum’s acid derivative **8** using standard conditions.^10^ On the basis of previous structure–activity relationships of the pilicide/curlicide central fragment, a phenyl,^9^, ^15^ a 3-trifluoromethylphenyl,^10^, ^22^ a 2-thiophenyl,^17^ and a cyclopropyl^9^, ^15^ were selected as substituents on the Δ^2^-thiazolines (**9 a**–**d**). Reacting **8** with the **9 a**–**d** in the acyl ketene imine cyclocondensation gave coumarin-substituted thiazolo ring-fused 2-pyridones **10 a**–**d** in 68–86 % yield (Scheme 1). Compounds **10 a**–**d** were next subjected to hydrolysis to give the corresponding carboxylic acids **11 a**–**d** in 53–66 % yield.

**Scheme 2:**
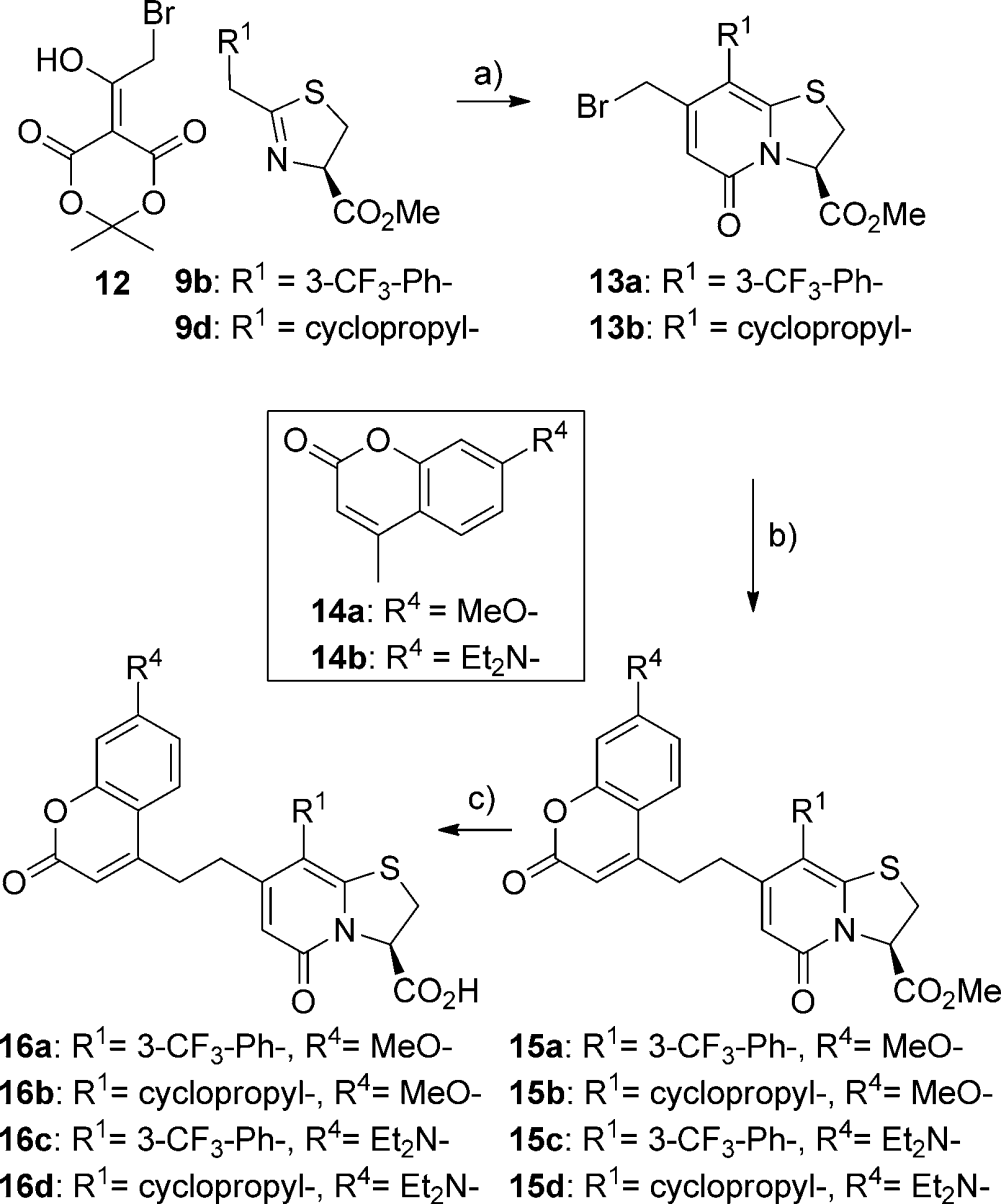
a) TFA, dichloroethane, MWI: 120 °C, 140 s (**13 a**,**b**: 92 and 87 %, respectively); b) **14 a** or **14 b**, LiHMDS, THF, −35 °C, 1 h (**15 a**–**d**: 74, 82, 83 and 75 %, respectively); c) For **16 a**,**c**: 0.1 M aq LiOH, THF/MeOH (4:1) 1.5 h; ii) AcOH (**16 a**,**c**: 86 and 82 %, respectively), For **16 b**,**d**: i) LiBr, TEA, MeCN (+2 v/v % H_2_O), RT, 3 h; ii) AcOH (**16 b**,**d**: 91 and 81 %, respectively).

The photophysical evaluation of **11 a**–**d** showed relatively low quantum yields of fluorescence (*Φ*_F_, Table 1). The C8 phenyl-substituted **11 a** displayed highest quantum yield (*Φ*_F_=5 %), followed by the C8 3-trifluomethylphenyl-substituted **11 b** (*Φ*_F_=1 %), the C8 2-thiophenyl-substituted **11 c** (*Φ*_F_=0.7 %), and finally the C8 cyclopropyl-substituted **11 d** (*Φ*_F_=0.4 %). These results may be due to a quenching effect of the fluorophore by the neighboring dihydrothiazolo ring-fused 2-pyridone pilicide/curlicide central fragment. In an attempt to circumvent this, the linker between the fluorophore and the 2-pyridone central fragment was increased by a one carbon extension. This was realized by using deprotonated 4-methyl coumarins as nucleophiles on bromomethyl-substituted 2-pyridone central fragments. By using this strategy the introduction of both a 7-methoxy-substituted coumarin and a 7-diethylamine-substituted coumarin could be accomplished (Scheme 2). The intermediate bromomethyl-substituted 2-pyridone central fragments (**13 a** and **13 b**) were synthesized following a previously published procedure.^23^ Addition of lithiated coumarins (**14 a** and **14 b**) to **13 a** and **13 b** generated the one carbon extended coumarin-substituted ring-fused 2-pyridones **15 a**–**d** in 74–83 % yield. Subsequent hydrolysis rendered **16 a**–**d** in high yields (81–91 %; Scheme 2).

**Table 1:** The fluorophore-substituted compounds photophysical properties and abilities to inhibit pili- and curli-dependent biofilm formation. 
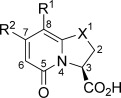

ID	R^1^ (C8)	R^2^ (C7)	X	EC_50_^[a]^ Pili [μM]	EC_50_^[a]^ Curli [μM]	*λ*_abs_^[b,c]^ [nm]	*λ*_fl_^[b,d]^ [nm]	Quantum Yield^[b]^ *Φ*_F_ [%] (*λ*_ex_ nm)
**11 a**	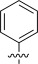	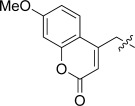	-S-	>200	NA^[e]^	328	394	5 (330)^[f]^
**11 b**	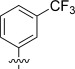	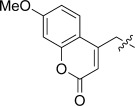	-S-	>200	NA^[e]^	330	420	1 (330)^[f]^
**11 c**	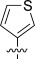	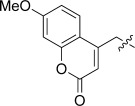	-S-	>200	NA^[e]^	328	396	0.7 (330)^[f]^
**11 d**	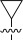	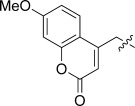	-S-	>200	NA^[e]^	328	413	0.4 (330)^[f]^
**16 a**	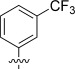	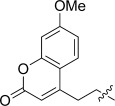	-S-	65	175	329	430	0.6 (355)^[f]^
**16 b**	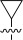	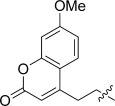	-S-	156	NA^[e]^	327	393	0.5 (330)^[f]^
**16 c**	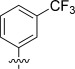	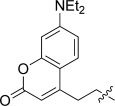	-S-	18	25	393	474	15 (390)^[g]^
**16 d**	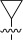	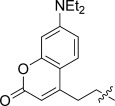	-S-	12	NA^[e]^	392	484	6 (330)^[f]^
**23**	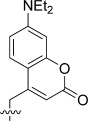	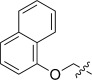	-S-	5	17	396	478	11 (346)^[f]^
**28**	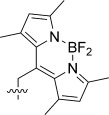	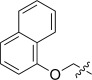	-S-	4	14	506	524	10 (470)^[h]^
**31**	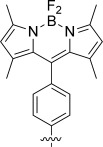	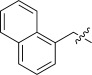	-S-	14	12	502	514	67 (470)^[h]^
**34**	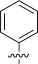	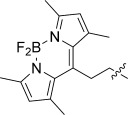	-S-	10	24	498	509	11 (480)^[h]^
**37**	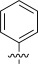	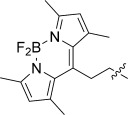	-O-	13	14	497	531	27 (480)^[h]^
**38**	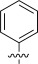	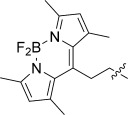	-S(O)-	29	40	498	516	71 (480)^[h]^
**3**	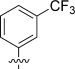	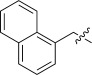	-S-	17	38	–	–	–

[a] Estimated from 16–32 data points on every concentration. [b] All substances were dissolved in DMSO and subsequently diluted in phosphate buffer at pH 7.0. The samples DMSO concentrations never exceed 5 wt %. The sample concentrations in the DMSO stock solutions are adjusted so that the final samples never have a peak absorbance higher than 0.1. [c] Wavelengths of the peak absorption. [d] The peak fluorescence. [e] Not active. [f] Reference: POPOP in MeOH. [g] Reference: Perylene in cyclohexane. [h] Reference: Rhodamine 6G in water.

**Scheme 3:**
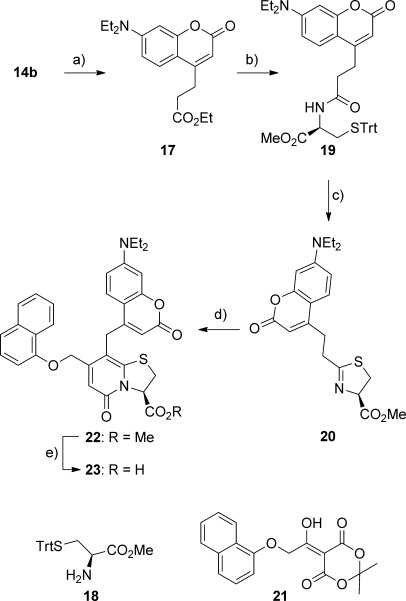
a) LiHMDS, DMPU, ethyl 2-bromoacetate, THF, −20 °C, 35 min, 77 %; b) i) 1 M (aq) LiOH, THF, RT, 16 h, ii) HBTU, **18**, DMF, RT, 17 h, 60 %; c) TiCl_4_, CH_2_Cl_2_, 0 °C to RT, 16 h, 40 %; d) **21**, TFA, dichloroethane, MWI: 120 °C, 3 min, 73 %; e) 0.1 M aqueous LiOH, THF, RT, 1 h; ii) AcOH, 91 %.

The one carbon extended linker did not influence the quantum yield (**16 a**, *Φ*_F_=0.6 % and **16 b** *Φ*_F_=0.5 %; Table 1). However, replacing the 7-(methoxy)coumarin by a 7-(diethylamino)coumarin as in **16 c** and **16 d** increased the quantum yields (*Φ*_F_=15 and 6 %, respectively, Table 1) and gave C7 coumarin-substituted compounds with useful fluorescent properties. As a consequence, the 7-(diethylamino)coumarin was used in the introduction of a coumarin in the C8 position of the pilicide/curlicide central fragment. The coumarin-substituted Δ^2^-thiazoline **20** was synthesized in three steps starting from **14 b** and ethyl 2-bromoacetate (Scheme 3).^24^ Subsequent reaction with acyl Meldrum’s acid derivative **21** rendered the C8 coumarin-substituted central fragment **22** in 73 % yield (Scheme 3). After hydrolysis of **22** into the target compound **23** the photophysical properties were evaluated. From this a quantum yield comparable with the other 7-(diethylamino)coumarin-substituted compounds was observed for **23** (*Φ*_F_=11 %, Table 1). Thus, compounds with a coumarin fluorophore in both the C7 and C8 positions of the pilicide/curlicide central fragment have been synthesized.

**Scheme 4:**
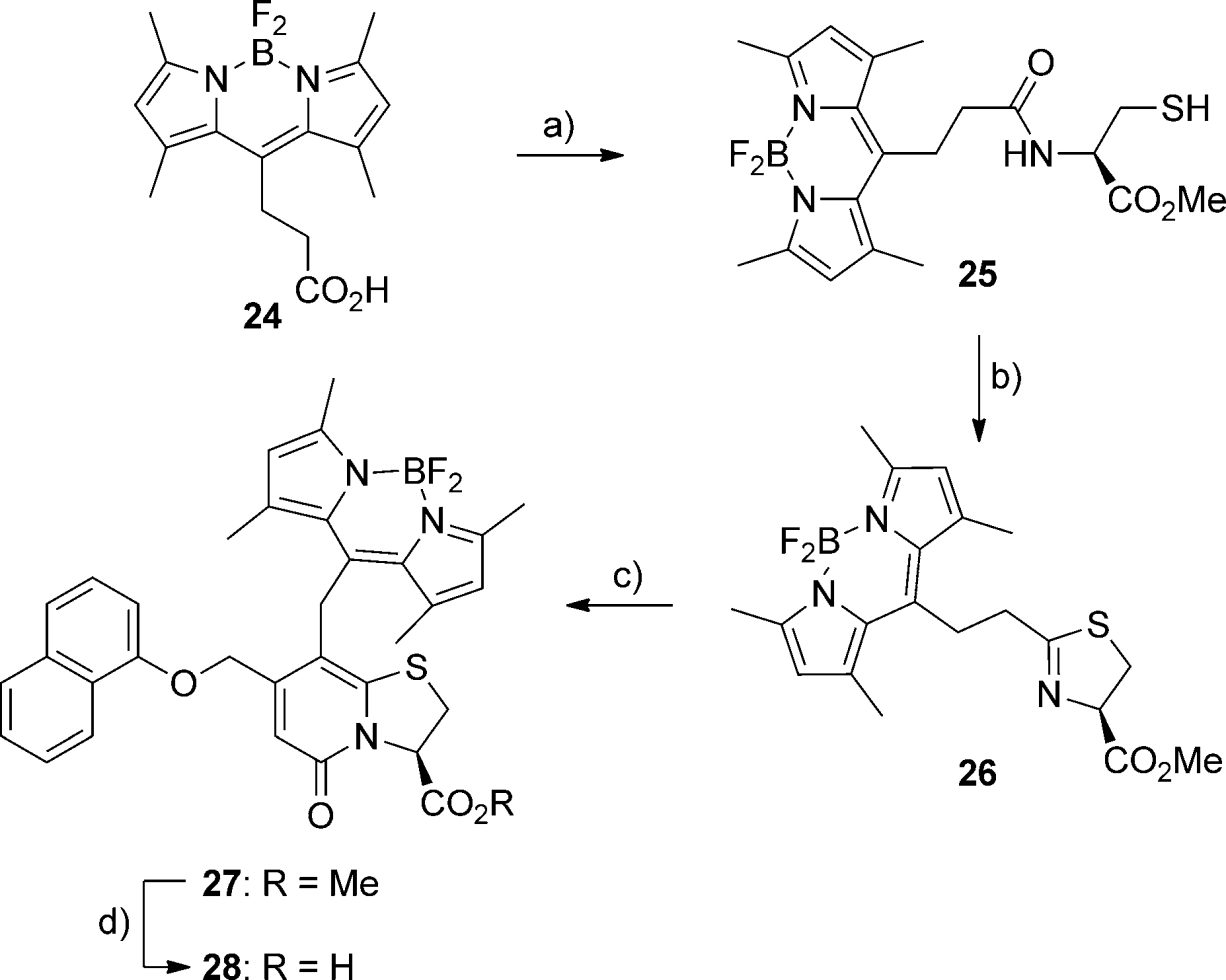
a) i) (COCl)_2_, CH_2_Cl_2_, RT, 17 h; ii) TEA, cysteine methyl ester, CH_2_Cl_2_, 0 °C to RT, 4.5 h, 64 %; b) i) TiCl_4_, CH_2_Cl_2_, 0 °C to RT, 4.5 h; ii) BF_3_**⋅**OEt_2_, CH_2_Cl_2_, RT, 40 min, 75 %; c) **21**, TFA, dichloroethane, MWI: 120 °C, 140 s, 64 %; d) i) LiI, pyridine, MWI: 140 °C, 15 min; ii) TEA, BF_3_**⋅**OEt_2_, dichloroethane, 80 °C, 15 min, 29 %.

To increase the probability of identifying a bioactive compound with useful fluorescence properties, the possibility of introducing a different fluorophore on the central fragment was investigated. 4,4-difluoro-4-bora-3a,4a-diaza-*s*-indacene (BODIPY) is a known fluorophore that normally gives high quantum yields, carries no net charges, is relatively insensitive to the choice of solvent, and should give a different emission color as compared to the coumarins.^20^ The synthesis of the BODIPY core structure is often accompanied by low yields. The desired 8-propanoic acid-functionalized 1,3,5,7-tetramethyl-substituted BODIPY (**24**) has previously been synthesized in 21 % yield.^25^ From **24**, the introduction of a BODIPY substituent in the C8 position of the pilicide/curicide central fragment could be pursued. Coupling **24** using a standard coupling procedure with methylester-protected cysteine gave the intermediate **25** in 64 % yield (Scheme 4). Ring closure of **25** to give **26** followed by acyl-ketene imine cyclocondensation with Meldrum’s acid derivative **21** gave the BODIPY-substituted dihydrothiazolo ring-fused 2-pyridone **27** in 64 % yield. The following hydrolysis proved to be problematic and the harsh conditions needed for this transformation ultimately gave **28** in 29 % yield as a racemate (Scheme 4). However, on the basis of previous reports showing that both enantiomers of the pilicide central fragment are biologically active, it was still entirely possible that racemic **28** could display interesting bioactivity.^9^, ^26^

**Scheme 5:**
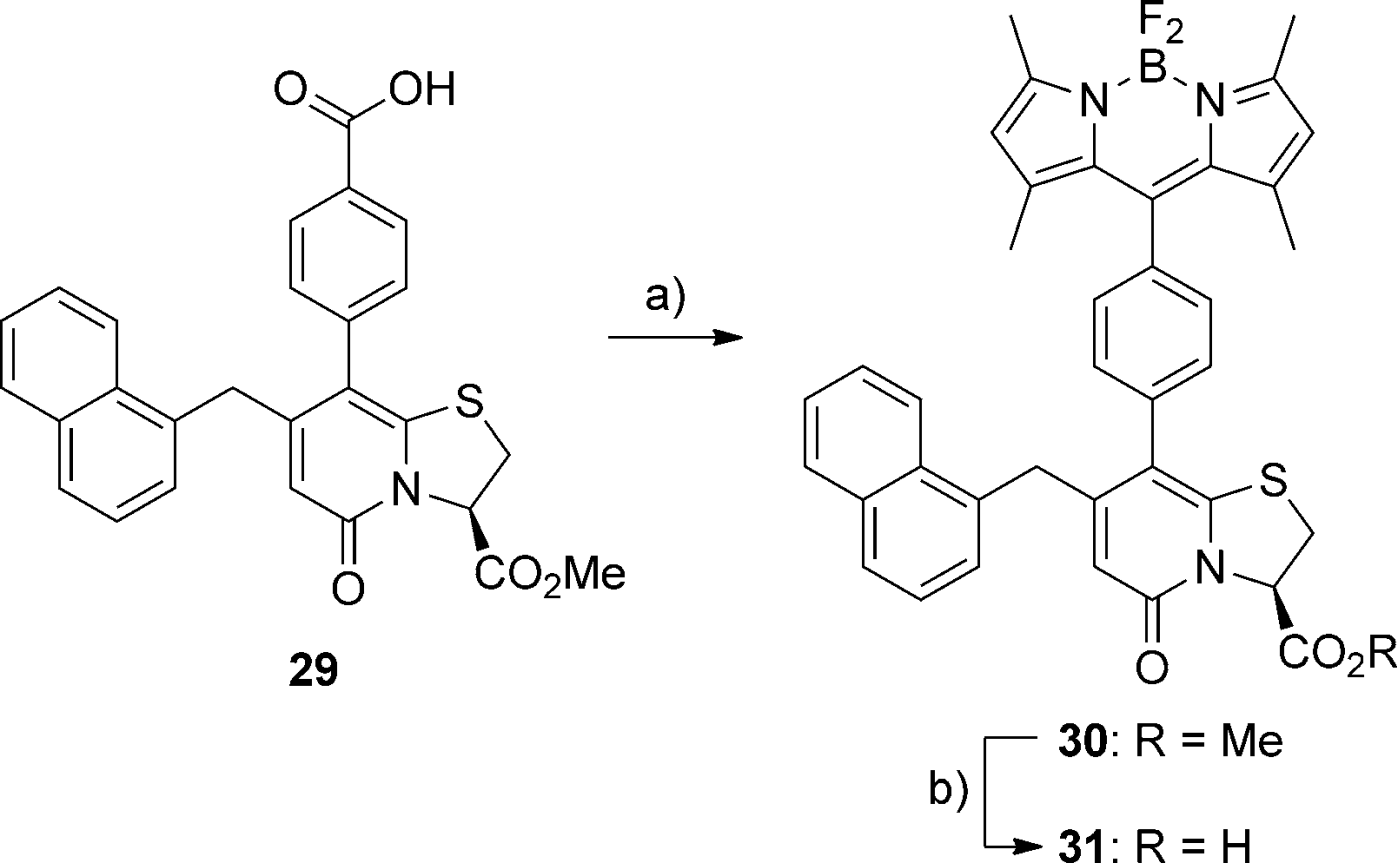
a) i) (COCl)_2_, DMF, CH_2_Cl_2_, RT, 1 h; ii) TEA, BF_3_**⋅**OEt_2_, 2,4-dimethylpyrrole, dichloroethane, MWI: 140 °C, 50 min, 15 %; b) 0.1 M aqueous LiOH, THF, RT, 1 h; ii) H^+^, 84 %.

The photophysical evaluation of **28** gave a quantum yield of 10 %, which is surprisingly low for a BODIPY-substituted compound (Table 1). We hypothesized that photo-quenching was a possible reason for this and thus the use of an aryl linker between the fluorophore and the central fragment could circumvent the problem. This would not only increase the distance between the fluorophore and the pilicide/curlicide central fragment but it would also restrict the rotation of the 1,3,5,7-tetramethyl-substituted BODIPY, which is known to generate higher quantum yields.^20^ Exchange of the methylene linker for a phenyl results in the need for a revised synthetic approach. A recent publication shows that it is possible to introduce a benzoic acid in the C8 position of the pilicide/curlicide central fragment by Suzuki–Miyaura couplings.^27^ From this benzoic acid derivative, the transformation into the desired C8 BODIPY-substituted central fragment seemed feasible. Consequently, 2-pyridone **29** was treated with oxalyl chloride followed by reaction with 2,4-dimethylpyrrole in the presence of BF_3_**⋅**OEt_2_ and triethylamine to give the desired C8 BODIPY-substituted central fragment **30** in 15 % yield. Subsequent hydrolysis was straightforward for this BODIPY derivative, giving the corresponding carboxylic acid **31** in 84 % yield (Scheme 5). As hypothesized, introduction of the phenyl spacer in the C8 position increased the quantum yield of **31** to a satisfactory 67 % (Table 1).

**Scheme 6:**
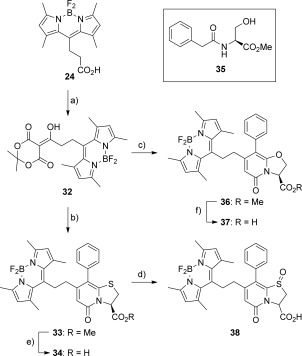
a) DCC, Meldrum’s acid, DMAP, CH_2_Cl_2_, 0 °C RT, 8 h, 82 %; b) **9 a**, TFA, dichloroethane, MWI: 120 °C, 140 s, 85 %; c) i) **35**, (NH_4_)_2_MoO_4_, toluene, reflux soxhlet (3 MS), 6 h; ii) **32**, TFA, toluene, reflux soxhlet (3 MS), 2 h, 73 %; d) starting from **34**, *m*CPBA, CH_2_Cl_2_, RT, 15 min, 80 %; e) i) 0.1 M aq LiOH, THF/MeOH (4:1); ii) AcOH, 88 %; f) i) LiBr, TEA, MeCN (+2 v/v % H_2_O), RT, 3 h; ii) AcOH, 88 %.

With these two C8 BODIPY-substituted compounds (**28** and **31**) in hand, introduction of BODIPY in position C7 on the central fragment was next investigated. Coupling the BODIPY carboxylic acid **24** with Meldrum’s acid using standard coupling conditions and a simple MeOH trituration as purification gave the BODIPY-substituted acyl Meldrum’s acid derivative **32** in 82 % yield (Scheme 6). From this versatile intermediate the following acylketene imine cyclocondensation to the C7 BODIPY substituted pilicide/curlicide central fragment **33** was performed in 88 % yield. Sequential hydrolysis gave the corresponding carboxylic acid **34** (Scheme 6). The photophysical properties of **34** suggest, in analogy with the coumarins, that the BODIPY fluorophore also seems to be partially quenched in this position (*Φ*_F_=11 %, Table 1).

Even though the quantum yields for many of the synthesized compounds are acceptable and, in the case of **31**, high, the underlying reason for the observed photo-quenching was still unknown. Thus, we further investigated the role of the sulfur in position 1 of the pilicide/curlicide central fragment on the photophysical properties. The sulfur in the central fragment was first replaced by an oxygen by reacting **32** and serine derivative **35** in a previously reported two-step one-pot procedure to give **36** in 73 % yield (Scheme 6).^28^, ^29^ Hydrolysis of **36** was straightforward and gave **37** in 88 % yield. The quantum yield of **37** (*Φ*_F_=29 %, Table 1) was almost three times greater than that of its sulfur-containing counterpart **34** (*Φ*_F_=11 %, Table 1). This suggests that the sulfur at position 1 of the pilicide/curlicide central fragment is at least partially responsible for the photo-quenching. To further verify this, the electronic properties of the sulfur were altered through oxidation. Treating **34** with *m*CPBA gave the sulfoxide **38** in 80 % yield as a 4:1 mixture of diastereomers (Scheme 6). Photophysical evaluation of sulfoxide **38** showed a great increase of quantum yield (*Φ*_F_=71 %, Table 1) compared to the parent sulfide **34** (*Φ*_F_=11 %, Table 1) thus supporting the theory that the sulfide in position 1 of the central fragment is involved in the observed partial photo-quenching.

The 14 fluorophore-substituted compounds were biologically evaluated for their ability to block pili and curli formation as measured by pili- or curli-dependent biofilm assays on polyvinylchloride surfaces.^10^, ^11^, ^30^ The amount of biofilm that is formed in the *E. coli* clinical isolate UTI89 grown in the presence of pilicide or curlicide, is related to the potency of the compound in blocking piliation or curliation. The results are summarized in Table 1.

The strategy of generating fluorescent pilicides by replacing key pilicide substituents with fluorophores based on structure–activity knowledge was successful and many of the tested compounds were both highly potent and fluorescent. Several of the compounds were able to inhibit formation of both pili- and curli-dependent biofilm, which is highly valuable from a therapeutic perspective. From the pili-dependent biofilm evaluation, C8 substitution with a methylene spacer using both coumarin (**23**) and BODIPY (**28**) fluorophores gave the most potent pilicides with estimated half-maximal effective concentrations (EC_50_) of 5 and 4 μM, respectively. Interestingly, compound **28** is also among the most potent inhibitors of curli-dependent biofilm formation with an estimated EC_50_ of 14 μM and is thus a potent dual pilicide–curlicide. This should be compared with the best reported di-substituted compound **3** (known as FN075), which exhibited EC_50_ values of 17 μM in pili-dependent biofilm inhibition and 38 μM in curli-dependent biofilm inhibition (Table 1). The phenyl spacer in **31** resulted in a slight decreased pilicide activity compared to the methylene linker in **28**, but is still in the same range as **3**. The C7 BODIPY-substituted derivatives all displayed good inhibitory properties of pili- and curli-dependent biofilm formation. The sulfide (**34**) and oxygen analogue (**37**) are more potent than the sulfoxide (**39**) both as pilicides and curlicides (Table 1). However, the fact that the sulfoxide retains much of the biofilm-inhibition activity might be of importance from a therapeutic and drug metabolism perspective. For the coumarin-substituted compounds with ethylene linker (**16 a**–**d**), the 7-(diethylamino)coumarins **16 c**,**d** are more active pilicides than the 7-(methoxy)coumarins **16 a**,**b** (Table 1). Furthermore, the compounds having an ethylene linker are more potent pilicides than the compounds with a methylene linker (**16 a** and **16 b** compared with **11 b** and **11 d**). None of the four 7-(methoxy)coumarins with a methylene linker **11 a**–**d** showed any activity (Table 1).

Finally, to study the compounds distribution in a bacterial population, UPEC strain UTI89 was grown under pili-producing conditions (24 h at 37 °C static) with compounds **28** and **34** and examined by fluorescence microscopy (Figure 2). Compounds **28** and **34** appeared to bind bacteria differently, with **34** appearing more punctate and **28** appearing more diffuse. Further experiments are working to examine the reason for these observed differences. We know that even under pilus-inducing conditions, not all bacteria are expressing pili. Thus it is likely that the pilicide is only binding bacteria expressing pili. Experiments are underway to confirm this hypothesis.

## Conclusion

We have herein developed compounds that are both fluorescent and highly active inhibitors of pili- and curli-dependent biofilm formation. The synthesis of these compounds was initiated to facilitate profound studies of the pilicides and curlicides and to gain information about the complex systems involved in the formation of pili and curli. To create these fluorescent and bioactive compounds we implemented a strategy based on structure–activity information, in which important substituents for bioactivity on the pilicide/curlicide central fragment were replaced by fluorophores. Synthetic methods were developed to enable a total of 14 compounds with either a coumarin or a BODIPY motif in the C7 or the C8 position. Consequently, new interesting intermediates and new reactions to introduce BODIPY fluorophores (e.g., via Meldrum’s acid derivatives and subsequent acyl ketene cyclocondensations) have been developed. Photo-quenching was frequently observed but its origin was elucidated and circumvented to give compounds with useful fluorescence properties. Biological evaluation using whole bacterial pili- and curli-biofilm assays revealed several compounds that are both fluorescent and highly active. Finally, treatment of UPEC strain UTI89 with the compounds under pili-producing conditions shows that the compounds are associated to the bacteria with a heterogeneous distribution over a population. The use of these compounds to study the biological systems they interact with, for example, the relation between the observed staining pattern of the compounds and the heterogeneous distribution of pili production in bacterial populations, is a matter of future studies within our laboratories.

## Experimental Section

**General synthesis***:* All reactions were carried out under an inert atmosphere with dry solvents under anhydrous conditions, unless otherwise stated. CH_2_Cl_2_ and 1,2-dichloroethane (DCE) and was distilled from calcium hydride and THF was distilled from potassium. DMF was distilled and dried over 3 Å molecular sieves. All microwave reactions were carried out in a monomode reactor (Smith Synthesizer, Biotage AB) using Smith process vials sealed with a Teflon septa and an aluminum crimp top. Reaction times refer to the irradiation time at the target temperature not the total irradiation time. The temperature was measured with an IR sensor. Flash column chromatography (eluent given in brackets) employed normal phase silica gel (Matrex, 60 Å, 35–70 μm, Grace Amicon). The ^1^H and ^13^C NMR spectra were recorded at 298 K with a Bruker DRX-400 spectrometer in CDCl_3_ (residual CHCl_3_ (*δ*_H_=7.26 ppm) or CDCl_3_ (*δ*_C_=77.0 ppm) as internal standard), [D_6_]DMSO (residual [D_5_]DMSO (*δ*_H_=2.50 ppm) or [D_6_]DMSO (*δ*_C_=40.0 ppm) as internal standard), [D_6_]Acetone (residual [D_5_]Acetone (*δ*_H_=2.05 ppm) or [D_6_]Acetone (*δ*_C_=29.9 ppm) as internal standard), and [D_4_]MeOH (residual [D_3_]MeOH (*δ*_H_=3.30 ppm) or [D_4_]MeOH (*δ*_C_=49.0 ppm) as internal standard). IR spectra were recorded on an ATI Mattson Genesis Series FTIR spectrometer. Optical rotations were measured with a PerkinElmer 343 polarimeter at 20 °C. MS data were recorded using electron spray (ES+) ionization on a Waters Micromass ZQ 2000 spectrometer. HRMS was obtained Micromass Q-Tof Ultima mass spectrometer and [*M*+H]^+^ molecular ions were generated by electrospray ionization.

**(3*R*)-7-((7-Methoxy-2-oxo-2*H*-chromen-4-yl)methyl)-5-oxo-8-phenyl-3,5-dihydro-2*H*-thiazolo[3,2-a]pyridine-3-carboxylic acid (11 a)**: LiOH (0.1 M, 1.5 mL, 1 equiv) was added dropwise to a stirred solution of **10 a** (71.3 mg, 0.15 mmol, 1 equiv) dissolved in THF/MeOH (2 mL, 4:1). The reaction mixture was stirred for two hours at room temperature before being concentrated. Purification by silica gel chromatography (CH_2_Cl_2_/MeOH/AcOH, 90:8:2) and then lyophilized from MeCN/H_2_O (1:3) to give **11 a** (47 mg, 66 %). [*α*]_D_=−17 (*c*=0.25 in CHCl_3_); ^1^H NMR (400 MHz, [D_6_]DMSO): *δ*=3.49 (dd, *J*_1_=1.79 Hz, *J*_2_=11.82 Hz, 1 H), 3.72–3.88 (m, 6 H), 5.48 (dd, *J*_1_=1.63 Hz, *J*_2_=9.01 Hz, 1 H), 5.93 (s, 1 H), 6.01 (s, 1 H), 6.88 (dd, *J*_1_=2.50 Hz, *J*_2_=8.83 Hz, 1 H), 6.96 (d, *J=*2.49, 1 H), 7.20–7.28 (m, 1 H), 7.29–7.36 (m, 2 H), 7.36–7.44 ppm (m, 3 H); ^13^C NMR (100 MHz, [D_6_]DMSO): *δ*=32.4, 35.7, 56.8, 64.6, 101.9, 112.8, 113.1, 115.0, 115.3 (2C), 127.0, 129.2, 129.8 (2C), 130.8 (broad, 2C), 136.8, 149.6, 150.8, 154.0, 155.8, 160.79, 160.83, 163.3, 170.4 ppm; IR: 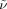
=1712, 1608, 1554, 1484, 1423, 1388, 1257, 1207, 1025, 836, 802, 701 cm^−1^; HRMS (EI): *m/z*: calcd for C_25_H_20_NO_6_S: 462.1011 [*M*+H]; found: 462.1018.

**(3*R*)-7-((7-Methoxy-2-oxo-2*H*-chromen-4-yl)methyl)-5-oxo-8-(3-(trifluoromethyl)phenyl)-3,5-dihydro-2*H*-thiazolo[3,2-a]pyridine-3-carboxylic acid (11b)**: Prepared according to the procedure described for compound **11 a** starting from **10 b** (49 mg, 0.09 mmol, 1 equiv), giving **11 b** (10 mg, 58 %). [*α*]_D_=−4 (*c*=0.51 in CHCl_3_); ^1^H NMR (400 MHz, [D_4_]MeOH/CDCl_3_ 1:1): *δ*=3.60–3.68 (m, 1 H), 3.68–3.78 (m, 3 H), 3.83 (s, 3 H), 5.57 (d, *J*=8.11 Hz, 1 H), 5.97 (s, 1 H), 6.22 (s, 1 H), 6.73–6.81 (m, 2 H), 7.21 (d, *J*=8.73, 1 H), 7.39–7.61 ppm (m, 5 H); ^13^C NMR (100 MHz, [D_4_]MeOH/CDCl_3_ 1:1): *δ*=33.1, 35.8, 56.2, 66.4, 101.5, 112.6, 112.8, 113.3, 115.6, 115.9, 124.3 (q, *J*=272.34 Hz), 125.9, 126.0, 127.4 (d, *J*=29.1 Hz), 130.4, 131.9 (q, *J*=32.42 Hz), 134.2 (d, *J*=34.73 Hz), 137.1, 150.9, 151.0, 154.1, 155.8, 162.3, 162.7, 163.8, 172.5 ppm; IR: 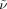
=1716, 1609, 1484, 1434, 1388, 1330, 1288, 1211, 1126, 848, 705 cm^−1^; HRMS (EI): *m/z*: calcd for C_26_H_19_F_3_NO_6_S: 530.0885 [*M*+H]; found: 530.0880.

**(3*R*)-7-((7-Methoxy-2-oxo-2*H*-chromen-4-yl)methyl)-5-oxo-8-(thiophen-2-yl)-3,5-dihydro-2*H*-thiazolo[3,2-a]pyridine-3-carboxylic acid (11c)**: Prepared according to the procedure described for compound **11 a** starting from **10 c** (72.2 mg, 0.15 mmol, 1 equiv) to give **11 c** (38 mg, 53 %). [*α*]_D_=−17 (*c*=0.69 in CHCl_3_); ^1^H NMR (400 MHz, [D_6_]DMSO): *δ*=3.51 (dd, *J*_1_=1.77 Hz, *J*_2_=11.61 Hz, 1 H), 3.78–3.83 (m, 1 H), 3.85 (s, 2 H), 5.43 (dd, *J*_1_=1.52 Hz, *J*_2_=9.16 Hz, 1 H), 5.91 (s, 1 H), 6.04 (s, 1 H), 6.91 (dd, *J*_1_=2.56 Hz, *J*_2_=8.88 Hz, 1 H), 6.98 (d, *J*=2.54 Hz, 1 H), 7.03–7.07 (m, 2 H), 7.48 (d, *J*=8.88 Hz, 1 H), 7.56–7.60 ppm (m, 1 H). ^13^C NMR (100 MHz, [D_6_]DMSO): *δ*=32.6, 35.8, 56.9, 65.1, 101.9, 107.1, 112.78, 112.81, 113.1, 115.5, 126.9, 128.4, 128.9, 130.4, 136.8, 151.5, 152.4, 154.2, 155.8, 160.7, 160.9, 163.3, 170.4 ppm; IR: 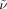
=1712, 1646, 1608, 1481, 1438, 1388, 1280, 1207, 1145, 1018, 987, 840, 705 cm^−1^; HRMS (EI): *m/z*: calcd for C_23_H_18_NO_6_S_2_: 468.0576 [*M*+H]; found: 468.0587.

**(3*R*)-8-Cyclopropyl-7-((7-methoxy-2-oxo-2*H*-chromen-4-yl)methyl)-5-oxo-3,5-dihydro-2*H*-thiazolo[3,2-a]pyridine-3-carboxylic acid (11 d)**: Prepared according to the procedure described for compound **11 a** starting from **10 d** (49.0 mg, 0.11 mmol, 1 equiv) to give **11 d** (31.2 mg, 66 %). [*α*]_D_=−58 (*c*=0.20 in CHCl_3_); ^1^H NMR (400 MHz, [D_6_]DMSO): *δ*=0.51–0.71 (m, 2 H) 0.76–0.88 (m, 2 H), 1.56–1.66 (m, 1 H), 3.52 (dd, *J*_1_=1.76 Hz, *J*_2_=11.86 Hz, 1 H), 3.82 (dd, *J*_1_=9.08 Hz, *J*_2_=11.87 Hz, 1 H), 3.86 (s, 3 H), 4.10–4.27 (m, 2 H), 5.40 (dd, *J*_1_=1.75 Hz, *J*_2_=9.08 Hz, 1 H), 5.79 (s, 1 H), 6.03 (s, 1 H), 6.97 (dd, *J*_1_=2.55 Hz, *J*_2_=8.87 Hz, 1 H), 7.03 (d, *J*=2.51 Hz, 1 H), 7.66 ppm (d, *J*=8.88 Hz, 1 H); ^13^C NMR (100 MHz, [D_6_]DMSO): *δ*=7.6, 7.8, 11.2, 32.2, 34.9, 56.5, 63.8, 101.6, 111.8, 112.4, 112.7, 112.9, 114.5, 127.0, 149.6, 152.9, 154.3, 155.6, 160.4, 160.6, 163.0, 170.1 ppm; IR: *ν<*Ü=>1712, 1643, 1608, 1481, 1438, 1388, 1280, 1207, 1141, 1041, 1022, 987, 840, 705 cm^−1^; HRMS (EI): *m/z*: calcd for C_22_H_20_NO_6_S: 426.1011 [*M*+H]; found: 426.1016.

**(3*R*)-7-(2-(7-Methoxy-2-oxo-2*H*-chromen-4-yl)ethyl)-5-oxo-8-(3-(trifluoromethyl)phenyl)-3,5-dihydro-2*H*-thiazolo[3,2-a]pyridine-3-carboxylic acid (16 a)**: LiOH (0.1 M, 0.36 mL, 1 equiv) was added drop wise to a stirred solution of **15 a** (20 mg, 36 μmol, 1 equiv) dissolved in THF/MeOH (1 mL, 4:1). The reaction mixture was left for approximately one and a half hours at room temperature. The solution was then concentrated, and co-concentrated from MeOH three times. Purification by silica gel chromatography (CH_2_Cl_2_/MeOH, 92:8, 90:8 + 2 % AcOH), concentrated and co-concentrated from CH_2_Cl_2_ and chloroform and then lyophilized from MeCN/H_2_O (1:3) giving **16 a** as a white powder (17 mg, 86 %). [*α*]_D_=2 (*c*=0.51 in CHCl_3_); ^1^H NMR (400 MHz, [D_4_]MeOH/CDCl_3_ 11:9): *δ*=2.62–2.73 (m, 2 H), 2.75–2.84 (m, 2 H), 3.55–3.62 (m, 1 H), 3.75–3.84 (m, 1 H), 3.84 (s, 3 H), 5.64–5.70 (m, 1 H), 5.89 (s, 1 H), 6.34 (s, 1 H), 6.9 (dd, *J*_1_=2.19 Hz, *J*_2_=8.64 Hz, 1 H), 6.78 (d, *J=*2.42 Hz, 1 H), 6.91 (t, *J=*8.54 Hz, 1 H), 7.44–7.51 (m, 1 H), 7.52–7.64 (m, 2 H), 7.67–7.73 ppm (m, 1 H); ^13^C NMR (100 MHz, [D_4_]MeOH/CDCl_3_ 11:9): *δ*=32.46, 32.49, 32.9 (split), 56.1, 64.7, 101.7, 111.4, 112.7, 113.3, 114.6, 115.7, 124.4 (q, *J*=272.22 Hz), 125.6 (split), 126.0 (split), 127.5 (split), 130.7 (d, *J*=5.08 Hz), 132.2 (q, *J*=32.56 Hz), 134.5 (d, *J*=30.19 Hz), 137.5, 150.0, 154.8, 156.05, 156.07, 162.8, 163.7, 169.9, 170.0 ppm; IR: 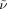
=1712, 1612, 1484, 1438, 1388, 1284, 1207, 1145, 1045, 1022, 987, 840, 705 cm^−1^; HRMS (EI): *m/z*: calcd for C_27_H_21_F_3_NO_6_S: 544.1042 [*M*+H]; found: 544.1049.

**(3*R*)-8-Cyclopropyl-7-(2-(7-methoxy-2-oxo-2*H*-chromen-4-yl)ethyl)-5-oxo-3,5-dihydro-2*H*-thiazolo[3,2-a]pyridine-3-carboxylic acid (16 b)**: By following a previously published procedure,^31^ **15 b** (25 mg, 0.056 mmol) was hydrolyzed to its corresponding carboxylic acid **16 b** (91 % yield). [*α*]_D_=−4 (*c*=0.3 in CHCl_3_/MeOH 2:1); ^1^H NMR (400 MHz, CDCl_3_): *δ*=0.56–0.66 (m, 2 H), 0.85–1.02 (m, 2 H), 1.50–1.60 (m, 1 H), 2.96–3.16 (m, 4 H), 3.64–3.74 (m, 1 H), 3.75–3.83 (m, 1 H), 3.87 (s, 3 H), 5.65–5.73 (m, 1 H), 6.14 (s, 1 H), 6.41 (s, 1 H), 6.81–6.91 (m, 2 H), 7.51–7.59 (m, 1 H), 8.9–9.4 ppm (bs, 1 H); ^13^C NMR (100 MHz, CDCl_3_): *δ*=7.5, 7.9, 11.0, 30.7, 30.9, 31.0, 55.8, 64.2, 101.2, 111.0, 112.4, 112.6, 113.0, 116.2, 125.1, 150.0, 154.7, 155.5, 157.9, 161.3, 162.4, 162.8, 168.4 ppm; IR: 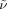
=1714, 1609, 1486, 1207, 1145, 1024, 835 cm^−1^; HRMS (EI): *m/z*: calcd for C_23_H_22_NO_6_S: 440.1168 [*M*+H]; found: 440.1169.

**(3*R*)-7-(2-(7-(Diethylamino)-2-oxo-2*H*-chromen-4-yl)ethyl)-5-oxo-8-(3-(trifluoromethyl)phenyl)-3,5-dihydro-2*H*-thiazolo[3,2-a]pyridine-3-carboxylic acid (16c)**: Prepared according to the procedure described for compound **16 a**, starting from **15 c** (20 mg, 0.036 mmol, 1 equiv) gave **16 c** as a pale yellow powder (41 mg, 82 %). [*α*]_D_=3 (*c*=0.60 in CHCl_3_); ^1^H NMR (400 MHz, [D_4_]MeOH): *δ*=1.18 (t, *J*=6.99 Hz, 6 H), 2.59–2.78 (m, 4 H), 3.38 (q, *J*=7.03 Hz, 4 H), 3.52–3.80 (m, 2 H), 5.48–5.74 (m, 2 H), 6.27–6.50 (m, 3 H), 6.65–6.81 (m, 1 H), 7.42–7.75 ppm (m, 4 H); ^13^C NMR (100 MHz, [D_6_]DMSO): *δ*=12.2 (2C), 31.1, 32.0 (broad, split), 32.7 (broad, split), 43.9, 65.3 (broad), 79.2, 96.9, 106.7, 106.9, 108.3, 112.2, 113.7, 124.0 (q, *J*=272.59 Hz), 124.8, 125.1, 126.8 (d, *J*=9.48 Hz), 129.7 (q, *J*=31.61 Hz), 130.2, 134.7, 137.7, 148.7, 150.1, 152.0, 155.4, 155.8, 160.3, 160.7, 167.7 ppm; IR: 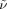
=1708, 1596, 1527, 1484, 1415, 1330, 1272, 1160, 1122, 1072, 802, 705 cm^−1^; HRMS (EI): *m/z*: calcd for C_30_H_28_F_3_N_2_O_5_S: 585.1671 [*M*+H]; found: 585.1667.

**(3*R*)-8-Cyclopropyl-7-(2-(7-(diethylamino)-2-oxo-2*H*-chromen-4-yl)ethyl)-5-oxo-3,5-dihydro-2*H*-thiazolo[3,2-a]pyridine-3-carboxylic acid (16 d)**: By following a previously published procedure,^31^ **15 d** (27.5 mg, 0.056 mmol) was hydrolyzed to its corresponding carboxylic acid **16 d** (81 % yield). [*α*]_D_=5 (*c*=0.13 in MeOH); ^1^H NMR (400 MHz, CDCl_3_): *δ*=0.55–0.67 (m, 2 H), 0.86–1.03 (m, 2 H), 1.21 (t, *J*=7.10 Hz, 6 H), 1.51–1.61 (m, 1 H), 2.90–3.13 (m, 4 H), 3.41 (q, *J*=7.07 Hz, 4 H), 3.61–3.70 (m, 1 H), 3.82–3.89 (m, 1 H), 5.64–5.71 (m, 1 H), 5.94 (s, 1 H), 6.33 (s, 1 H), 6.49–6.53 (s, 1 H), 6.57–6.62 (m, 1 H), 7.38–7.43 ppm (m, 1 H); ^13^C NMR (100 MHz, CDCl_3_): *δ*=7.4, 7.9, 11.0, 12.4 (2C), 30.4, 30.6, 31.3, 44.7 (2C), 64.2, 97.9, 107.6, 107.8, 108.7, 113.3, 115.9, 124.9, 149.5, 150.6, 154.9, 156.3, 158.0, 162.2, 162.7, 168.4 ppm; IR: 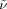
=1710, 1613, 1596, 1487, 1415, 1139, 825 cm^−1^; HRMS (EI): *m/z*: calcd for C_26_H_29_N_2_O_5_S: 481.1797 [*M*+H]; found: 481.1794.

**(3*R*)-8-((7-(Diethylamino)-2-oxo-2*H*-chromen-4-yl)methyl)-7-((naphthalen-1-yloxy)methyl)-5-oxo-3,5-dihydro-2*H*-thiazolo[3,2-a]pyridine-3-carboxylic acid (23)**: Compound **22** (0.34 mmol, 0.2 g) was dissolved in THF (20 mL) and LiOH (0.44 mmol, 0.1 M, 4.4 mL) was added, the reaction was stirred at RT for 1 h. The solvent was evaporated and the crude product was purified with column chromatography on silica gel (first pure EtOAc then CH_2_Cl_2_/MeOH/AcOH 90:5:5) to give **23** as a yellow solid (180 mg, 91 % yield). [*α*]_D_=−10 (*c*=0.5 in CHCl_3_); ^1^H NMR (400 MHz, [D_6_]DMSO): *δ*=7.99 (d, *J*=8.5 Hz, 1 H), 7.82 (d, *J*=8.1 Hz, 1 H), 7.60 (d, *J*=9.1 Hz, 1 H), 7.52–7.30 (m, 4 H), 6.96 (d, *J*=7.7 Hz, 1 H), 6.62 (dd, *J*=9.2, 2.4 Hz, 1 H), 6.48 (s, 1 H), 6.44 (d, *J*=2.4 Hz, 1 H), 5.55–5.49 (m, 1 H), 5.47 (s, 1 H), 5.17–5.06 (m, 2 H), 4.05–3.87 (m, 3 H), 3.65–3.58 (m, 1 H), 3.45–3.34 (m, 4 H), 1.10 ppm (t, 6 H); ^13^C NMR (100 MHz, [D_6_]DMSO): *δ*=169.4, 160.8, 160.1, 155.6, 153.1, 153.0, 150.3, 150.1, 149.5, 133.9, 127.4, 126.4, 125.8, 125.5, 125.3, 124.7, 121.1, 120.5, 113.2, 108.5, 107.3, 105.6, 105.4, 105.2, 96.8, 66.5, 63.3, 44.0 (2C), 31.6, 30.6, 12.3 ppm (2C); HRMS (EI): *m/z*: calcd for C_33_H_30_N_2_O_6_S: 583.1903[*M*+H^+^]; found: 583.1900.

**7-((Naphthalen-1-yloxy)methyl)-5-oxo-8-((1,3,5,7-tetramethyl-4,4-difluoro-4-bora-3a,4a-diaza-s-indacene-8-yl)methyl)-3,5-dihydro-2H-thiazolo[3,2-a]pyridine-3-carboxylic acid (28)**: LiI (111 mg, 0.83 mmol) was added to a solution **27** (52 mg, 0.08 mmol) in dry pyridine (2 mL) while stirring. The reaction mixture was heated to 140 °C for 15 min by microwave irradiation. The reaction mixture was allowed to attain RT, diluted with CH_2_Cl_2_ and washed with 2 % KHSO_4_. The aqueous layer was extracted three times with CH_2_Cl_2_ and the combined organic layers were dried over Na_2_SO_4_, filtered and concentrated in vacuo. The resulting dark oil was diluted in 1,2-dichloroethane (2 mL) and TEA (58 μL, 0.42 mmol) was added dropwise while stirring. After 5 min BF_3_**⋅**OEt_2_ was added and the reaction mixture was heated to 80 °C for 15 min. After being allowed to attain RT the reaction mixture was diluted with CH_2_Cl_2_ and washed with water. The aqueous layer was extracted five times with CH_2_Cl_2_ and the combined organic layers were concentrated in vacuo. Purification by column chromatography (CH_2_Cl_2_, 2 % MeOH→CH_2_Cl_2_, 2 % MeOH, 1 % AcOH) gave **28** as an orange solid (15 mg, 29 %). [*α*]_D_=0; ^1^H NMR (400 MHz, [D_6_]DMSO): *δ*=2.21 (s, 3 H), 2.29 (s, 3 H), 2.43 (s, 6 H), 3.39 (dd, *J*_1_=1.26 Hz, *J*_2_=11.88 Hz, 2 H), 3.56 (dd, *J*_1_=8.76 Hz, *J*_2_=11.88 Hz, 2 H), 4.27 (d, *J*=16.73 Hz, 1 H), 4.35 (d, *J*=16.73 Hz, 1 H), 5.36–5.40 (m, 3 H), 6.18–6.24 (m, 2 H), 6.58 (s, 1 H), 7.17–7.23 (m, 1 H), 7.43–7.49 (m, 1 H), 7.53–7.61 (m, 3 H), 7.89–7.95 (m, 1 H), 8.24–8.29 ppm (m, 1 H); ^13^C NMR (100 MHz, [D_6_]DMSO): *δ*=14.7, 14.72, 15.3, 15.8, 28.53, 32.1, 62.0, 66.8, 105.1, 106.7, 113.4, 121.2, 121.7, 122.1, 122.1, 125.2, 126.2, 127.1, 128.1, 134.1 (broad), 134.6, 138.9, 142.4 (broad), 144.8, 150.6, 153.4, 154.5, 154.9 (broad), 160.5, 169.8 ppm; IR: 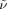
=1647, 1553, 1506, 1198, 1159, 982 cm^−1^; HRMS (EI): *m/z*: calcd for C_33_H_31_BF_2_N_3_O_4_S: 614.2096 [*M*+H]; found: 614.2097.

**(3*R*)-7-(Naphthalen-1-ylmethyl)-5-oxo-8-(4-(1,3,5,7-tetramethyl-4,4-difluoro-4-bora-3a,4a-diaza-*s*-indacene-8-yl)phenyl)-3,5-dihydro-2*H*-thiazolo[3,2-a]pyridine-3-carboxylic acid (31)**: Compound **30** (0.045 mmol, 30 mg) was dissolved in THF (5 mL) and LiOH (0.09 mmol, 0.1 M, 0.9 mL) was added, the reaction was stirred at RT for 1 h. The reaction mixture was diluted with water and pH was set to 1 with 1 M HCl (aq). The waterphase was extracted with EtOAc (2×50 mL), the combined organic phases was dried (Na_2_SO_4_), filtered and concentrated. The crude product was purified with HPLC, (C18, 250×21.2 mm, 5 μm, 0–100 % MeCN over 1 h) and lyophilized to give **31** as a red solid (25 mg, 84 % yield). [*α*]_D_=20 (*c* 0.05, CHCl_3_); ^1^H NMR (400 MHz, [D_6_]DMSO): *δ*=7.96–7.88 (m, 1 H), 7.85–7.78 (m, 1 H), 7.78–7.71 (m, 1 H), 7.64–7.34 (m, 7 H), 7.31–7.25 (m, 1 H), 6.18 (s, 1 H), 6.03 (s, 1 H), 5.70 (s, 1 H), 5.55–5.46 (m, 1 H), 4.09 (s, 2 H), 3.92–3.83 (m, 1 H), 3.57–3.49 (m, 1 H), 2.44 (s, 3 H), 2.41 (s, 3 H), 1.42 (s, 3 H), 0.93 ppm (s, 3 H); ^13^C NMR (100 MHz, [D_6_]DMSO): *δ*=169.5, 160.0, 154.9 (2C), 152.8, 148.2, 142.6 (2C), 141.4, 137.2, 134.0, 133.8, 133.3, 131.2 (4C), 130.6, 128.6, 128.2 (2C), 127.3, 126.9, 126.3, 125.8, 125.5, 123.4, 121.5 (2C), 114.2, 113.9, 63.4, 35.5, 31.4, 14.2 (2C), 13.8, 13.3 ppm; HRMS (EI): *m/z*: calcd for C_38_H_32_BF_2_N_3_O_3_S: 660.2304 [*M*+H^+^]; found: 660.2303.

**(3R)-5-Oxo-8-phenyl-7-(2-(1,3,5,7-tetramethyl-4,4-difluoro-4-bora-3a,4a-diaza-*s*-indacene-8-yl)ethyl)-3,5-dihydro-2H-thiazolo[3,2-a]pyridine-3-carboxylic acid (34)**: Compound **34** was synthesized by following the same procedure described for **11 a** starting from **33** (30 mg, 0.053 mmol). Purification by column chromatography (CH_2_Cl_2_/MeOH 97:3 to CH_2_Cl_2_/MeOH/AcOH 96:3:1) gave **34** as a red non-crystalline solid (25.5 mg, 88 %). [*α*]_D_=34 (*c*=0.05 in MeOH); ^1^H NMR (400 MHz, [D_6_]DMSO): *δ*=2.25 (s, 6 H), 2.36 (s, 6 H), 2.39–2.49 (m, 2 H), 3.14–3.25 (m, 2 H), 3.43–3.50 (m, 1 H), 3.73–3.82 (m, 1 H), 5.40–5.47 (m, 1 H), 6.19 (s, 2 H), 6.30 (s, 1 H), 7.18–7.24 (m, 1 H), 7.25–7.36 (m, 2 H), 7.36–7.43 ppm (m, 2 H); ^13^C NMR (100 MHz, [D_6_]DMSO): *δ*=14.1 (m, 2C), 15.7 (2C), 25.5, 31.7, 33.4, 64.0, 111.5, 113.8, 121.8 (broad), 128.2, 129.0 (2C), 129.9 130.1, 130.5, 136.2, 140.9 (broad), 144.9, 147.9, 152.0, 153.6 (broad), 160.2, 169.5 ppm; IR: 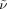
=1636, 1548, 1509, 1489, 1407, 1198, 981, 704 cm^−1^; HRMS (EI): *m/z*: calcd for C_29_H_29_BF_2_N_3_O_3_S: 548.1991 [*M*+H]; found: 548.1972.

**(3*R*)-5-Oxo-8-phenyl-7-(2-(1,3,5,7-tetramethyl-4,4-difluoro-4-bora-3a,4a-diaza-*s*-indacene-8-yl)ethyl)-3,5-dihydro-2*H*-oxazolo[3,2-a]pyridine-3-carboxylic acid (37)**: Compound **37** was synthesized by following a previously published procedure,^29^ starting from **36** (51 mg, 0.094 mmol). Purification by column chromatography (CH_2_Cl_2_/MeOH 97:3 to CH_2_Cl_2_/MeOH/AcOH 96:3:1) gave **37** as a red non-crystalline solid (27 mg, 54 %). [*α*]_D_=−26 (*c* 0.1, CHCl_3_); ^1^H NMR (400 MHz, [D_6_]DMSO): *δ*=2.23 (bs, 6 H), 2.35 (s, 6 H), 2.44–2.68 (m, 2 H), 3.05–3.29 (m, 2 H), 4.58 (dd, *J*_1_=3.78 Hz, *J*_2_=8.92 Hz, 1 H), 4.78 (t, *J*=9.02 Hz, 1 H), 4.96 (dd, *J*_1_=3.72 Hz, *J*_2_=9.21 Hz, 1 H), 6.08 (s, 1 H), 6.16 (s, 2 H), 7.19–7.28 (m, 3 H), 7.29–7.37 ppm (m, 2 H); ^13^C NMR (100 MHz, [D_6_]DMSO): *δ*=14.1 (m, 2C), 15.6 (2C), 26.0, 33.3, 58.3, 72.8, 97.8, 107.1, 121.8 (broad), 127.3, 128.4 (2C), 130.5 (2C), 130.8 (broad), 132.8, 140.8 (broad), 145.0, 153.6 (broad), 153.9, 154.4, 158.3, 169.6 ppm; IR: 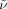
=1659, 1549, 1510, 1197, 981, 703 cm^−1^; HRMS: *m/z*: calcd for C_29_H_29_BF_2_N_3_O_4_: 532.2219 [*M*+H]; found: 532.2213.

**1,5-Dioxo-8-phenyl-7-(2-(1,3,5,7-tetramethyl-4,4-difluoro-4-bora-3a,4a-diaza-*s*-indacene-8-yl)ethyl)-3,5-dihydro-2*H*-thiazolo[3,2-a]pyridine-3-carboxylic acid (38)**: *m*CPBA (70 %, 9 mg, 0.37 mmol) was added to **34** (18.6 mg, 0.034 mmol) in CH_2_Cl_2_ (1.5 mL) at RT. The solution was stirred for 15 min before quenching with Na_2_S_2_O_5_ (aq. saturated). The resulting solution was extracted three times with CH_2_Cl_2_, dried with Na_2_SO_4_, filtered, and concentrated. Purification by column chromatography (CH_2_Cl_2_/acetone 9:1 to CH_2_Cl_2_/acetone/AcOH 88:10:2) gave **38** as a red foam (15.4 mg, 80 % yield). [*α*]_D_=−10 (*c*=0.1 in DMSO); ^1^H NMR (400 MHz, [D_6_]DMSO): *δ*=2.26 (s, 6 H), 2.39 (s, 6 H), 2.54–2.64 (m, 2 H), 3.14–3.32 (m, 2 H), 3.53–3.62 (m, 2 H), 5.40–5.49 (m, 1 H), 6.02 (s, 2 H), 6.79 (s, 1 H), 7.30–7.49 ppm (m, 5 H); ^13^C NMR (100 MHz, [D_6_]DMSO): *δ*=14.1 (2C), 15.6 (2C), 25.4, 33.2, 51.0, 65.2, 119.9, 120.5, 121.9 (broad), 128.47, 128.54, 128.6 130.4, 130.5 (broad), 131.1, 133.4, 140.9 (broad), 144.6, 149.0, 151.8, 153.7 (broad), 159.0, 170.1 ppm; IR: 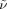
=1650, 1549, 1509, 1407, 1197, 981, 704 cm^−1^; HRMS: *m/z*: calcd for C_29_H_29_BF_2_N_3_O_4_S: 564.1940 [*M*+H]; found: 564.1928.
