# Supplementary material for: Design and Synthesis of Fluorescent Pilicides and Curlicides: Bioactive Tools to Study Bacterial Virulence Mechanisms
Source: Chemistry. 2012 Mar 16;18(15):4522–32. doi: 10.1002/chem.201103936 (PMC3569613; doi:10.1002/chem.201103936)
Supplement: Supplementary file 1 [file chem0018-4522-sd1.pdf]

CHEMISTRY
A EUROPEAN JOURNAL

Supporting Information

© Copyright Wiley-VCH Verlag GmbH & Co. KGaA, 69451 Weinheim, 2012

Design and Synthesis of Fluorescent Pilicides and Curlicides: Bioactive Tools to Study Bacterial Virulence Mechanisms

Erik Chorell–,[a, c] Jerome S. Pinkner,[b, c] Christoffer Bengtsson,[a] Sofie Edvinsson,[a] Corinne K. Cusumano,[b, c] Erik Rosenbaum,[a] Lennart B. Å. Johansson,[a] Scott J. Hultgren,*[b, c] and Fredrik Almqvist*[a, d]

chem_201103936_sm_miscellaneous_information.pdf

Experimental Section

General synthesis: All reactions were carried out under an inert atmosphere with dry solvents under anhydrous conditions, unless otherwise stated. DCM and DCE and was distilled from calcium hydride and THF was distilled from potassium. DMF was distilled and dried over 3Å molecular sieves. All microwave reactions were carried out in a monomode reactor (Smith Synthesizer, Biotage AB) using Smith Process Vials™ sealed with Teflon septa and an aluminium crimp top. Reaction times refer to irradiation time at the target temperature not the total irradiation time. The temperature was measured with an IR sensor. Flash column chromatography (eluent given in brackets) employed normal phase silica gel (Matrex, 60 Å, 35-70 µm, Grace Amicon). The ^{1}H and ^{13}C NMR spectra were recorded at 298 K with a Bruker DRX-400 spectrometer in $CDCl_3$ [residual $CHCl_3$ (δ_H 7.26 ppm) or $CDCl_3$ (δ_C 77.0 ppm) as internal standard], [D_6]DMSO [residual [D_5]DMSO (δ_H 2.50 ppm) or [D_6]DMSO (δ_C 40.0 ppm) as internal standard], [D_6]Acetone [residual [D_5]Acetone (δ_H 2.05 ppm) or [D_6]Acetone (δ_C 29.9 ppm) as internal standard], and [D_4]MeOH [residual [D_3]MeOH (δ_H 3.30 ppm) or [D_4]MeOH (δ_C 49.0 ppm) as internal standard]. IR spectra were recorded on an ATI Mattson Genesis Series FTIR™ spectrometer. Optical rotations were measured with a Perkin-Elmer 343 polarimeter at 20 °C. MS data were recorded using electron spray (ES+) ionization on a Waters Micromass ZQ 2000 spectrometer. HRMS was obtained Micromass Q-Tof Ultima™ mass spectrometer and $(M+H)^+$ molecular ions were generated by electrospray ionization.

9a, **13a**, **13b**, and **35** were synthesized according to published procedures. Data in agreement with published data.[1-3]

(3R)-Methyl 7-((7-methoxy-2-oxo-2H-chromen-4-yl)methyl)-5-oxo-8-phenyl-3,5-dihydro-2H-thiazolo[3,2-a]pyridine-3-carboxylate (10a). Synthesis of **8**: DCC (4.11 g, 19.90 mmol, 2 eq) was added to 2-(7-methoxy-2-oxochroman-4-yl) acetic acid (2.33 g, 9.95 mmol, 1 eq) dissolved in 60 mL of DMF at 0 °C. The solution was left to stir for 45 minutes. Meldrum's acid (1.58 g, 10.94 mmol, 1.1 eq) was added to the solution followed by DMAP (1.94 g, 15.92 mmol, 1.6 eq) and the reaction mixture was left stirring at room temperature over night. The reaction mixture was quenched using 6% $KHSO_4$ (aq.) and the resulting precipitate was filtered off. The filtrate was then extracted three times with CH_2Cl_2. The combined organic phases were extracted twice with $NaHCO_3$ (aq. saturated) and the combined aqueous phases were acidified using HCl (aq.) before being extracted with EtOAc. Drying with Na_2SO_4, filtration, and concentration gave **6** (2.22 g, 62%) as a white solid that was used without further purification. ^{1}H NMR (400 MHz, $CDCl_3$) δ 1.79 (s, 6H), 3.88 (s, 3H), 4.60 (s, 2H), 6.84-6.90 (m, 2H), 7.49 (d, J = 8.49 Hz, 1H). Synthesis of **10a**; **8** (0,27 g, 0.75 mmol) and **9a** (71 mg, 0.30 mmol) were dissolved in 3.5 mL DCE followed by dropwise addition of TFA (23 µl, 0.30 mmol). The reaction vessel was sealed and heated for 140 seconds at 120 °C using microwave irradiation. Purification by column chromatography using parallel equipment (heptane:EtOAc) gave **10a** as an orange solid (0.11 mg, 77%). $[\alpha]_D$ = -151 (c 0.5, $CHCl_3$); ^{1}H NMR (400 MHz, $CDCl_3$) δ 3.50 (dd, J_1 = 2.31 Hz, J_2 = 11.79 Hz, 1H), 3.57-3.75 (m, 3H), 3.83 (s, 3H), 3.84 (s, 3H), 5.65 (dd, J_1 = 2.30 Hz, J_2 = 8.56 Hz, 1H), 5.98 (s, 1H), 6.13 (s, 1H), 6.71-6.78 (m, 2H), 7.14-7.19 (m, 1H), 7.19-7.27 (m, 2H), 7.32-7.43 (m, 3H); ^{13}C NMR (100 MHz, $CDCl_3$) δ 31.7, 35.5, 53.3, 55.7, 63.6, 101.0, 112.0, 112.4, 113.0, 115.5, 115.6, 125.2, 128.7, 129.1, 129.5, 130.0, 135.5, 150.1, 152.2, 155.4, 160.7, 160.8, 162.7, 168.2; IR ν 1716, 1653, 1610, 1483, 1208, 1146, 842, 749, and 704 cm^{-1}; MS (electrospray ionization) calcd for [M + H] $C_{26}H_{22}NO_6S$ 476, obsd 476. Elemental analysis calcd (%) for $C_{26}H_{21}NO_6S$: C 65.67, H 4.45, N 2.95; found: C 65.2, H 4.5, N 3.0.

(3R)-Methyl 7-((7-methoxy-2-oxo-2H-chromen-4-yl)methyl)-5-oxo-8-(3-(trifluoromethyl)phenyl)-3,5-dihydro-2H-thiazolo[3,2-a]pyridine-3-carboxylate (10b). Prepared according to the procedure described for compound **10a** starting from **8** (30 mg, 0.087 mmol) and **9b** (11 mg, 37 µmol). Giving **10b** as a yellow solid (17 mg, 86%). $[\alpha]_D$ = -122 (c 0.5, $CHCl_3$); ^{1}H NMR (400 MHz, $CDCl_3$) δ 3.51 (dd, J_1 = 1.95 Hz, J_2 = 11.77 Hz, 1H), 3.62 (s, 2H), 3.73 (dd, J_1 = 8.61 Hz, J_2 = 11.80 Hz, 1H), 3.84 (s, 6H), 5.67 (dd, J_1 = 2.24 Hz, J_2 = 8.58 Hz, 1H), 5.98 (s, 1H), 6.17 (s, 1H), 6.72-6.78 (m, 2H), 7.12-7.16 (m, 1H), 7.47-7.63 (m, 4H); ^{13}C NMR (100 MHz, $CDCl_3$) δ 31.8, 35.4, 53.4, 55.8, 63.6, 101.0, 111.8, 112.5, 112.9, 113.8, 116.1, 123.5 (q, J = 272.0 Hz), 124.9, 125.5, 126.7 (d, J = 30.7 Hz), 129.7, 131.4 (q, J = 33.0 Hz), 133.4 (d, J = 40.0 Hz), 136.3, 148.4, 149.6, 151.9, 152.0, 155.3, 160.6, 162.8, 168.0; IR ν 1717, 1657, 1610, 1483, 1331, 1121, 842, and 707 cm^{-1}; MS

(electrospray ionization) calcd for [M + H] $C_{27}H_{21}F_3NO_6S$ 544, obsd 544. Elemental analysis calcd (%) for $C_{27}H_{20}F_3NO_6$: C 59.67, H 3.71, N 2.58; found: C 59.55, H 3.8, N 2.65.

(3R)-Methyl 7-((7-methoxy-2-oxo-2H-chromen-4-yl)methyl)-5-oxo-8-(thiophen-2-yl)-3,5-dihydro-2H-thiazolo[3,2-a]pyridine-3-carboxylate (10c). Prepared according to the procedure described for compound **10a** starting from **8** (0.270 g, 0.75 mmol) and **9c** (72 mg, 0.30 mmol) to give **10c** as a dark yellow solid (0.11 g, 77%). $[\alpha]_D$ = -44 (*c* 0.5, $CHCl_3$); ^1H NMR (400 MHz, $CDCl_3$) δ 3.51 (dd, J_1 = 2.35 Hz, J_2 = 11.80 Hz, 1H), 3.68-3.79 (m, 3H), 3.85 (s, 3H), 3.86 (s, 3H), 5.66 (dd, J_1 = 2.34 Hz, J_2 = 8.59 Hz, 1H), 6.01 (s, 1H), 6.12 (s,1H), 6.75-6.83 (m, 2H), 6.96 (dd, J_1 = 1.06 Hz, J_2 = 3.54 Hz, 1H), 7.02 (dd, J_1 = 3.54 Hz, J_2 = 5.16 Hz, 1H), 7.24 (s, 1H), 7.37 (dd, J_1 = 1.07 Hz, J_2 = 5.19 Hz, 1H); ^{13}C NMR (100 MHz, $CDCl_3$) δ 31.7, 35.6, 53.4, 55.7, 63.9, 101.0, 107.8, 112.1, 112.5, 113.0, 115.6, 125.3, 127.5, 127.7, 129.4, 135.6, 150.7, 151.1, 152.2, 155.4, 160.7, 160.8, 162.7, 168.1; IR ν 1714, 1652, 1610, 1483, 1208, 1146, 840, and 704 cm^{-1}; MS (electrospray ionization) calcd for [M + H] $C_{24}H_{20}NO_6S_2$ 482, obsd 482. Elemental analysis calcd (%) for $C_{24}H_{19}NO_6S_2$: C 59.86, H 3.98, N 2.91; found: C 59.2, H 3.9, N 2.9.

(3R)-Methyl 8-cyclopropyl-7-((7-methoxy-2-oxo-2H-chromen-4-yl)methyl)-5-oxo-3,5-dihydro-2H-thiazolo[3,2-a]pyridine-3-carboxylate (10d). Prepared according to the procedure described for compound **10a** starting from **8** (0.270 g, 0.75 mmol) and **9d** (60 mg, 0.30 mmol). Giving **10d** as a solid (90 mg, 68%). $[\alpha]_D$ = -184 (*c* 0.25, $CHCl_3$); ^1H NMR (400 MHz, $CDCl_3$) δ 0.58-0.70 (m, 2H) 0.80-0.95 (m, 2H), 1.47-1.58 (m, 1H), 3.54 (dd, J_1 = 1.55 Hz, J_2 = 11.64 Hz, 1H), 3.70 (dd, J_1 = 8.63 Hz, J_2 = 11.57 Hz, 1H), 3.80 (s, 3H), 3.88 (s, 3H), 3.92-4.21 (m, 2H), 5.60 (dd, J_1 = 1.45 Hz, J_2 = 8.32 Hz, 1H), 5.98-6.06 (m, 2H), 6.80-6.90 (m, 2H), 7.39-7.46 (m, 1H); ^{13}C NMR (100 MHz, $CDCl_3$) δ 7.2, 7.7, 10.9, 31.7, 35.1, 53.3, 55.8, 62.8, 101.1, 112.4, 112.5, 112.6, 112.9, 113.7, 125.2, 148.6, 152.4, 152.5, 155.5, 160.8, 160.9, 162.8, 168.4; IR ν 1714, 1651, 1609, 1487, 1207, 1145, 841, and 747 cm^{-1}; MS (electrospray ionization) calcd for [M + H] $C_{23}H_{22}NO_6S$ 440, obsd 440. Elemental analysis calcd (%) for $C_{23}H_{21}NO_6S$: C 62.86, H 4.82, N 3.19; found: C 62.6, H 4.8, N 3.2.

(3R)-7-((7-Methoxy-2-oxo-2H-chromen-4-yl)methyl)-5-oxo-8-phenyl-3,5-dihydro-2H-thiazolo[3,2-a]pyridine-3-carboxylic acid (11a). 0.1M LiOH (1.5 mL, 1 eq) was added dropwise to a stirred solution of **10a** (71.3 mg, 0.15 mmol, 1 eq) dissolved in 2 mL THF:MeOH (4:1). The reaction mixture was stirred for two hours at room temperature before being concentrated. Purification by silica gel chromatography (DCM:MeOH:AcOH, 90:8:2) and then lyophilized from MeCN:H_2O (1:3) to give **11a** (47 mg, 66%). $[\alpha]_D$ = -17 (*c* 0.25, $CHCl_3$); ^1H NMR (400 MHz, $[D_6]$DMSO) δ 3.49 (dd, J_1 = 1.79 Hz, J_2 = 11.82 Hz, 1H), 3.72-3.88 (m, 6H), 5.48 (dd, J_1 = 1.63 Hz, J_2 = 9.01 Hz, 1H), 5.93 (s, 1H), 6.01 (s, 1H), 6.88 (dd, J_1 = 2.50 Hz, J_2 = 8.83 Hz, 1H), 6.96 (d, J = 2.49, 1H), 7.20-7.28 (m, 1H), 7.29-7.36 (m, 2H), 7.36-7.44 (m, 3H); ^{13}C NMR (100 MHz, $[D_6]$DMSO) δ 32.4, 35.7, 56.8, 64.6, 101.9, 112.8, 113.1, 115.0, 115.3 (2C), 127.0, 129.2, 129.8 (2C), 130.8 (broad, 2C), 136.8, 149.6, 150.8, 154.0, 155.8, 160.79, 160.83, 163.3, 170.4; IR ν 1712, 1608, 1554, 1484, 1423, 1388, 1257, 1207, 1025, 836, 802, and 701 cm^{-1}; HRMS (electrospray ionization) calcd for [M + H] $C_{25}H_{20}NO_6S$ 462.1011, obsd 462.1018.

(3R)-7-((7-Methoxy-2-oxo-2H-chromen-4-yl)methyl)-5-oxo-8-(3-(trifluoromethyl)phenyl)-3,5-dihydro-2H-thiazolo[3,2-a]pyridine-3-carboxylic acid (11b). Prepared according to the procedure described for compound **11a** starting from **10b** (49 mg, 0.09 mmol, 1 eq), giving **11b** (10 mg, 58%). $[\alpha]_D$ = -4 (*c* 0.51, $CHCl_3$); ^1H NMR (400 MHz, $[D_4]$MeOH :$CDCl_3$ 1:1) δ 3.60-3.68 (m, 1H), 3.68-3.78 (m, 3H), 3.83 (s, 3H), 5.57 (d, J = 8.11 Hz, 1H), 5.97 (s, 1H), 6.22 (s, 1H), 6.73-6.81 (m, 2H), 7.21 (d, J = 8.73, 1H), 7.39-7.61 (m, 5H); ^{13}C NMR (100 MHz, $[D_4]$MeOH :$CDCl_3$ 1:1) δ 33.1, 35.8, 56.2, 66.4, 101.5, 112.6, 112.8, 113.3, 115.6, 115.9, 124.3 (q, J = 272.34 Hz), 125.9, 126.0, 127.4 (d, J = 29.1 Hz), 130.4, 131.9 (q, J = 32.42 Hz), 134.2 (d, J = 34.73 Hz), 137.1, 150.9, 151.0, 154.1, 155.8, 162.3, 162.7, 163.8, 172.5; IR ν 1716, 1609, 1484, 1434, 1388, 1330, 1288, 1211, 1126, 848, and 705 cm^{-1};HRMS (electrospray ionization) calcd for [M + H] $C_{26}H_{19}F_3NO_6S$ 530.0885, obsd 530.0880.

(3R)-7-((7-Methoxy-2-oxo-2H-chromen-4-yl)methyl)-5-oxo-8-(thiophen-2-yl)-3,5-dihydro-2H-thiazolo[3,2-a]pyridine-3-carboxylic acid (11c). Prepared according to the procedure described for compound **11a** starting from **10c** (72.2 mg, 0.15 mmol, 1 eq) to give **11c** (38 mg, 53%). $[\alpha]_D$ = -17 (*c* 0.69, $CHCl_3$); ^1H NMR (400 MHz, $[D_6]$DMSO) δ 3.51 (dd, J_1 = 1.77 Hz, J_2 = 11.61 Hz, 1H), 3.78-3.83 (m, 1H), 3.85 (s, 2H), 5.43 (dd, J_1 = 1.52 Hz, J_2 = 9.16 Hz, 1H), 5.91 (s, 1H), 6.04 (s, 1H), 6.91 (dd, J_1 = 2.56 Hz, J_2 = 8.88 Hz, 1H), 6.98 (d, J = 2.54 Hz, 1H), 7.03-7.07 (m, 2H), 7.48 (d, J = 8.88 Hz, 1H), 7.56-7.60 (m, 1H) ^{13}C NMR (100 MHz, $[D_6]$DMSO) δ 32.6, 35.8, 56.9, 65.1, 101.9, 107.1, 112.78, 112.81, 113.1, 115.5, 126.9, 128.4, 128.9, 130.4, 136.8, 151.5, 152.4, 154.2, 155.8, 160.7, 160.9, 163.3, 170.4; IR ν 1712, 1646, 1608, 1481, 1438, 1388, 1280, 1207, 1145, 1018, 987, 840, and 705 cm^{-1}; HRMS (electrospray ionization) calcd for [M + H] $C_{23}H_{18}NO_6S_2$ 468.0576, obsd 468.0587.

(3R)-8-Cyclopropyl-7-((7-methoxy-2-oxo-2H-chromen-4-yl)methyl)-5-oxo-3,5-dihydro-2H-thiazolo[3,2-a]pyridine-3-carboxylic acid (11d). Prepared according to the procedure described for compound **11a** starting from **10d** (49.0 mg, 0.11 mmol, 1 eq) to give **11d** (31.2 mg, 66%). $[\alpha]_D$ = -58 (*c* 0.20, $CHCl_3$); ^{1}H NMR (400 MHz, [D$_6$]DMSO) δ 0.51-0.71 (m, 2H) 0.76-0.88 (m, 2H), 1.56-1.66 (m, 1H), 3.52 (dd, J_1 = 1.76 Hz, J_2 = 11.86 Hz, 1H), 3.82 (dd, J_1 = 9.08 Hz, J_2 = 11.87 Hz, 1H), 3.86 (s, 3H), 4.10-4.27 (m, 2H), 5.40 (dd, J_1 = 1.75 Hz, J_2 = 9.08 Hz, 1H), 5.79 (s, 1H), 6.03 (s, 1H), 6.97 (dd, J_1 = 2.55 Hz, J_2 = 8.87 Hz, 1H), 7.03 (d, J = 2.51 Hz, 1H), 7.66 (d, J = 8.88 Hz, 1H); ^{13}C NMR (100 MHz, [D$_6$]DMSO) δ 7.6, 7.8, 11.2, 32.2, 34.9, 56.5, 63.8, 101.6, 111.8, 112.4, 112.7, 112.9, 114.5, 127.0, 149.6, 152.9, 154.3, 155.6, 160.4, 160.6, 163.0, 170.1; IR ν 1712, 1643, 1608, 1481, 1438, 1388, 1280, 1207, 1141, 1041, 1022, 987, 840, and 705 cm^{-1}; HRMS (electrospray ionization) calcd for [M + H] $C_{22}H_{20}NO_6S$ 426.1011, obsd 426.1016.

Preparation of 1.0M solution of LiHMDS: To a stirred solution of HMDS (1.03 mmol) in THF (0.117 mL/mmol) was added *n*BuLi (1.5 M, 1 mmol) at -35 °C. The solution was stirred for 30 min before being warmed to 0 °C for 10 min and again cooled to -35 °C before use.

(3R)-Methyl 7-(2-(7-methoxy-2-oxo-2H-chromen-4-yl)ethyl)-5-oxo-8-(3-(trifluoromethyl)phenyl)-3,5-dihydro-2H-thiazolo[3,2-a]pyridine-3-carboxylate (15a). 7-Methoxy-4-methylcoumarin (**14a**) (13 mg, 0.0675 mmol, 1.5 eq) dissolved in 1 mL of THF was added dropwise to a 1.0M solution of LHMDS at -35 °C. The resulting yellow suspension was stirred for 45 min at -35 °C before being added to **13a** (20 mg, 0.045 mmol, 1 eq) dissolved in 1 mL of THF at -35 °C. The reaction was quenched after 15 minutes with NH_4Cl (aq. sat.), extracted with EtOAc, and concentrated. Purification by column chromatography (CH_2Cl_2:MeOH, 98:2) gave **15a** as a white solid (18.5 mg, 74%). $[\alpha]_D$ = -17 (*c* 0.5, $CHCl_3$); ^{1}H NMR (400 MHz, $CDCl_3$) δ 2.55-2.84 (m, 4H), 3.50 (d, J = 11.80 Hz, 1H), 3.66-3.76 (m, 1H), 3.81-3.90 (m, 6H), 5.68 (dd, J_1 = 1.84 Hz, J_2 = 8.47 Hz, 1H), 5.86 (s, 1H), 6.31 (s, 1H), 6.67 (d, J = 8.64 Hz, 1H), 6.75 (d, J = 2.13 Hz, 1H), 6.89 (t, J = 9.32 Hz, 1H), 7.40-7.47 (m, 1H), 7.52-7.60 (m, 2H), 7.64-7.72 (m, 1H); ^{13}C NMR (100 MHz, $CDCl_3$) δ 31.7 (d, J = 8.19 Hz), 31.8 (broad), 32.1 (d, J = 12.03 Hz), 53.4, 55.7, 63.6, 101.1, 111.3, 112.0, 112.3, 114.0, 114.7, 123.6 (q, J = 272.39 Hz), 124.5, 125.4 (broad), 126.8 (broad and splitted), 129,8 (d, J = 5.80 Hz), 131.6 (q, J = 32.87 Hz), 133.6 (d, J = 31.37 Hz), 136.8, 148.0, 153.0, 153.8, 155.5, 160.9, 161.0, 162.6, 168.2; IR ν 1718, 1656, 1611, 1483, 1329, 1122, 841, and 708 cm^{-1}; MS (electrospray ionization) calcd for [M + H] $C_{28}H_{23}F_3NO_6S$ 558, obsd 558. Elemental analysis calcd (%) for $C_{28}H_{22}F_3NO_6S$: C 60.32, H 3.98, N 2.51; found: C 60.1, H 4.0, N 2.5.

(3R)-Methyl 8-cyclopropyl-7-(2-(7-methoxy-2-oxo-2H-chromen-4-yl)ethyl)-5-oxo-3,5-dihydro-2H-thiazolo[3,2-a]pyridine-3-carboxylate (15b). Prepared according to the procedure described for compound **15a**, starting from 7-Methoxy-4-methylcoumarin (**14a**) (24.5 mg, 0.128 mmol, 1.1 eq) and **13b** (40 mg, 0.116 mmol, 1 eq), giving **15b** as a yellow solid (43 mg, 82%). $[\alpha]_D$ = -26 (*c* 1.0, $CHCl_3$); ^{1}H NMR (400 MHz, $CDCl_3$) δ 0.56-0.67 (m, 2H), 0.84-0.98 (m, 2H), 1.49-1.59 (m, 1H), 2.96-3.09 (m, 4H), 3.47-3.54 (m, 1H), 3.63-3.71 (m, 1H), 3.80 (s, 3H), 3.87 (s, 3H), 5.57-5.63 (m, 1H), 6.13 (s, 1H), 6.18 (s, 1H), 6.81-6.91 (m, 2H), 7.50-7.57, (m, 1H); ^{13}C NMR (100 MHz, $CDCl_3$) δ 7.6, 7.7, 10.8, 30.7, 30.8, 31.6, 53.2, 55.8, 62.8, 101.2, 110.9, 112.4, 112.5, 113.1, 113.8, 124.9, 148.1, 154.7, 155.5, 156.0, 161.1, 161.2, 162.7, 168.5; IR ν 1748, 1714, 1653, 1614, 1579, 1490, 1296, 1215, 1145, and 844 cm^{-1}; MS (electrospray ionization) calcd for [M + H] $C_{24}H_{24}NO_6S$ 454, obsd 454. Elemental analysis calcd (%) for $C_{24}H_{23}NO_6S$: C 63.56, H 5.11, N 3.09; found: C 62.8, H 5.1, N 3.1.

(3R)-Methyl 7-(2-(7-(diethylamino)-2-oxo-2H-chromen-4-yl)ethyl)-5-oxo-8-(3-(trifluoromethyl)phenyl)-3,5-dihydro-2H-thiazolo[3,2-a]pyridine-3-carboxylate (15c). Prepared according to the procedure described for compound **15a**, starting from 7-Diethylamino-4-methylcoumarin (**14b**) (15.5 mg, 0.067 mmol, 1.5 eq) and **13a** (20 mg, 0.045 mmol, 1 eq), giving **15c** as a yellow solid (22 mg, 83%). ^{1}H NMR (400 MHz, $CDCl_3$) δ 1.19 (t, J = 6.95 Hz, 6H), 2.57-3.74 (m, 4H), 3.40 (q, J = 7.00 Hz, 4H), 3.50 (d, J = 11.76 Hz, 1H), 3.67-3.74 (m, 1H), 3.86 (s, 3H), 5.64-5.72 (m, 2H), 6.32 (s, 1H), 6.39 (d, J = 9.31 Hz, 1H), 6.43 (s, 1H), 6.75-6.83 (m, 1H), 7.40-7.48 (m, 1H), 7.52-7.60 (m, 2H), 7.62-7.72 (m, 1H); ^{13}C NMR (100 MHz, $CDCl_3$) δ 12.3 (2C), 31.7 (broad and splitted, 2C), 32.4 (d, J = 15.71 Hz), 44.6 (2C), 53.4, 63.6, 97.7, 107.3, 107.7, 108.4, 114.1, 114.6, 123.7 (q, J = 272.5 Hz), 124.4, 125.2 (d, J = 3.25 Hz), 126.8 (d, J = 27.04 Hz), 129.7 (d, J = 5.98 Hz), 131.4 (q, J = 32.73 Hz), 133.6 (d, J = 32.42 Hz), 136.8, 147.8, 150.4, 153.4, 154.2, 156.2, 161.0, 161.9, 168.2; IR ν 1714, 1652, 1613, 1124, and 708 cm^{-1}; MS (electrospray ionization) calcd for [M + H] $C_{31}H_{30}F_3N_2O_5S$ 599, obsd 599. Elemental analysis calcd (%) for $C_{31}H_{29}F_3N_2O_5S$: C 62.20, H 4.88, N 4.68; found: C 62.15, H 5.05, N 4.7.

(3R)-Methyl 8-cyclopropyl-7-(2-(7-(diethylamino)-2-oxo-2*H*-chromen-4-yl)ethyl)-5-oxo-3,5-dihydro-2*H*-thiazolo[3,2-*a*]pyridine-3-carboxylate (15d). Prepared according to the procedure described for compound **15a**, starting from 7-Diethylamino-4-methylcoumarin (**14b**) (29.5 mg, 0.128 mmol, 1.1 eq) and **13b** (40 mg, 0.116 mmol, 1 eq), giving **15d** as a yellow solid (43 mg, 75%). $[\alpha]_D$ = -5 (*c* 0.5, $CHCl_3$); ^{1}H NMR (400 MHz,

$CDCl_3$) δ 0.56-0.67 (m, 2H), 0.84-0.99 (m, 2H), 1.20 (t, J = 7.07 Hz, 6H), 1.50-1.59 (m, 1H), 2.90-3.08 (m, 4H), 3.41 (q, J = 7.08 Hz, 4H), 3.47-3.53 (m, 1H), 3.63-3.70 (m, 1H), 3.80 (s, 3H), 5.57-5.62 (m, 1H), 5.94 (s, 1H), 6.19 (s, 1H), 6.48-6.52 (m, 1H), 6.56-6.61 (m, 1H), 7.38-7.43 (m, 1H); ^{13}C NMR (100 MHz, $CDCl_3$) δ 7.6, 7.7, 10.9, 12.4 (2C), 30.7, 31.3, 31.6, 44.7 (2C), 53.2, 62.8, 97.9, 107.6, 107.8, 108.6, 113.3, 113.8, 124.9, 147.9, 150.6, 155.1, 156.3, 156.5, 161.2, 162.2, 168.6; IR ν 1709, 1653, 1613, 1596, 1414, 1200, 1139, and 824 cm^{-1}; MS (electrospray ionization) calcd for [M + H] $C_{27}H_{31}N_2O_5S$ 495, obsd 495. Elemental analysis calcd (%) for $C_{27}H_{30}N_2O_5S$: C 65.57, H 6.11, N 5.66; found: C 65.7, H 6.1, N 5.25.

(3R)-7-(2-(7-Methoxy-2-oxo-2H-chromen-4-yl)ethyl)-5-oxo-8-(3-(trifluoromethyl)phenyl)-3,5-dihydro-2H-thiazolo[3,2-a]pyridine-3-carboxylic acid (16a). 0.1M LiOH (0.36 mL, 1 eq) was added drop wise to a stirred solution of **15a** (20 mg, 36 µmol, 1 eq) dissolved in 1 mL THF:MeOH (4:1). The reaction mixture was left for approximately one and a half hours at room temperature. The solution was then concentrated and coconcentrated from MeOH 3 times. Purification by silica gel chromatography (DCM:MeOH, 92:8, 90:8 + 2% AcOH), concentrated and coconcentrated from DCM and chloroform and then lyophilized from MeCN:H_2O (1:3) giving **16a** as a white powder (17 mg, 86%). $[\alpha]_D$ = 2 (c 0.51, $CHCl_3$); ^{1}H NMR (400 MHz, [D$_4$]MeOH :$CDCl_3$ 11:9) δ 2.62-2.73 (m, 2H), 2.75-2.84 (m, 2H), 3.55-3.62 (m, 1H), 3.75-3.84 (m, 1H), 3.84 (s, 3H), 5.64-5.70 (m, 1H), 5.89 (s, 1H), 6.34 (s, 1H), 6.9 (dd, J_1 = 2.19 Hz, J_2 = 8.64 Hz, 1H), 6.78 (d, J = 2.42 Hz, 1H), 6.91 (t, J = 8.54 Hz, 1H), 7.44-7.51 (m, 1H), 7.52-7.64 (m, 2H), 7.67-7.73 (m, 1H); ^{13}C NMR (100 MHz, [D$_4$]MeOH :$CDCl_3$ 11:9) δ 32.46, 32.49, 32.9 (splitted), 56.1, 64.7, 101.7, 111.4, 112.7, 113.3, 114.6, 115.7, 124.4 (q, J = 272.22 Hz), 125.6 (splitted), 126.0 (splitted), 127.5 (splitted), 130.7 (d, J = 5.08 Hz), 132.2 (q, J = 32.56 Hz), 134.5 (d, J = 30.19 Hz), 137.5, 150.0, 154.8, 156.05, 156.07, 162.8, 163.7, 169.9, 170.0; IR ν 1712, 1612, 1484, 1438, 1388, 1284, 1207, 1145, 1045, 1022, 987, 840, and 705 cm^{-1}; HRMS (electrospray ionization) calcd for [M + H] $C_{27}H_{21}F_3NO_6S$ 544.1042, obsd 544.1049.

(3R)-8-Cyclopropyl-7-(2-(7-methoxy-2-oxo-2H-chromen-4-yl)ethyl)-5-oxo-3,5-dihydro-2H-thiazolo[3,2-a]pyridine-3-carboxylic acid (16b). By following a previously published procedure,[30] **15b** (25 mg, 0.056 mmol) was hydrolysed to its corresponding carboxylic acid **16b** (91% yield). $[\alpha]_D$ = -4 (c 0.3, $CHCl_3$:MeOH 2:1); ^{1}H NMR (400 MHz, $CDCl_3$) δ 0.56-0.66 (m, 2H), 0.85-1.02 (m, 2H), 1.50-1.60 (m, 1H), 2.96-3.16 (m, 4H), 3.64-3.74 (m, 1H), 3.75-3.83 (m, 1H), 3.87 (s, 3H), 5.65-5.73 (m, 1H), 6.14 (s, 1H), 6.41 (s, 1H), 6.81-6.91 (m, 2H), 7.51-7.59 (m, 1H), 8.9-9.4 (bs, 1H); ^{13}C NMR (100 MHz, $CDCl_3$) δ 7.5, 7.9, 11.0, 30.7, 30.9, 31.0, 55.8, 64.2, 101.2, 111.0, 112.4, 112.6, 113.0, 116.2, 125.1, 150.0, 154.7, 155.5, 157.9, 161.3, 162.4, 162.8, 168.4; IR ν 1714, 1609, 1486, 1207, 1145, 1024, and 835 cm^{-1}; HRMS (electrospray ionization) calcd for [M + H] $C_{23}H_{22}NO_6S$ 440.1168, obsd 440.1169.

(3R)-7-(2-(7-(Diethylamino)-2-oxo-2H-chromen-4-yl)ethyl)-5-oxo-8-(3-(trifluoromethyl)phenyl)-3,5-dihydro-2H-thiazolo[3,2-a]pyridine-3-carboxylic acid (16c). Prepared according to the procedure described for compound **16a**, starting from **15c** (20 mg, 0.036 mmol, 1 eq) gave **16c** as a pale yellow powder (41 mg, 82%). $[\alpha]_D$ = 3 (c 0.60, $CHCl_3$); ^{1}H NMR (400 MHz, [D$_4$]MeOH) δ 1.18 (t, J = 6.99 Hz, 6H), 2.59-2.78 (m, 4H), 3.38 (q, J = 7.03 Hz, 4H), 3.52-3.80 (m, 2H), 5.48-5.74 (m, 2H), 6.27-6.50 (m, 3H), 6.65-6.81 (m, 1H), 7.42-7.75 (m, 4H). ^{13}C NMR (100 MHz, [D$_6$]DMSO) δ 12.2 (2C), 31.1, 32.0 (broad, splitted), 32.7 (broad, splitted), 43.9, 65.3 (broad), 79.2, 96.9, 106.7, 106.9, 108.3, 112.2, 113.7, 124.0 (q, J = 272.59 Hz), 124.8, 125.1, 126.8 (d, J = 9.48 Hz), 129.7 (q, J = 31.61 Hz), 130.2, 134.7, 137.7, 148.7, 150.1, 152.0, 155.4, 155.8, 160.3, 160.7, 167.7; IR ν 1708, 1596, 1527, 1484, 1415, 1330, 1272, 1160, 1122, 1072, 802, and 705 cm^{-1}; HRMS (electrospray ionization) calcd for [M + H] $C_{30}H_{28}F_3N_2O_5S$ 585.1671, obsd 585.1667.

(3R)-8-Cyclopropyl-7-(2-(7-(diethylamino)-2-oxo-2H-chromen-4-yl)ethyl)-5-oxo-3,5-dihydro-2H-thiazolo[3,2-a]pyridine-3-carboxylic acid (16d). By following a previously published procedure,[30] **15d** (27.5 mg, 0.056 mmol) was hydrolysed to its corresponding carboxylic acid **16d** (81% yield). $[\alpha]_D$ = 5 (c 0.13, MeOH); ^{1}H NMR (400 MHz, $CDCl_3$) δ 0.55-0.67 (m, 2H), 0.86-1.03 (m, 2H), 1.21 (t, J = 7.10 Hz, 6H), 1.51-1.61 (m, 1H), 2.90-3.13 (m, 4H), 3.41 (q, J = 7.07 Hz, 4H), 3.61-3.70 (m, 1H), 3.82-3.89 (m , 1H), 5.64-5.71 (m, 1H), 5.94 (s, 1H), 6.33 (s, 1H), 6.49-6.53 (s, 1H), 6.57-6.62 (m, 1H), 7.38-7.43 (m, 1H); ^{13}C NMR (100 MHz, $CDCl_3$) δ 7.4, 7.9, 11.0, 12.4 (2C), 30.4, 30.6, 31.3, 44.7 (2C), 64.2, 97.9, 107.6, 107.8, 108.7, 113.3, 115.9, 124.9, 149.5, 150.6, 154.9, 156.3, 158.0, 162.2, 162.7, 168.4; IR ν 1710, 1613, 1596, 1487, 1415, 1139, and 825 cm^{-1}; HRMS (electrospray ionization) calcd for [M + H] $C_{26}H_{29}N_2O_5S$ 481.1797, obsd 481.1794.

5-(1-Hydroxy-2-(naphthalen-1-yloxy)ethylidene)-2,2-dimethyl-1,3-dioxane-4,6-dione (21). 2-(naphthalen-1-yloxy)acetic acid (5 g, 24.7 mmol) and DCC (5.87 g, 28.4 mmol) was dissolved in DCM (195 mL) at 0 °C and stirred for 30 min before 2,2-dimethyl-1,3-dioxane-4,6-dione (3.92 g, 27.2 mmol) and DMAP (4.83 g, 39.6 mmol) was added and the solution was allowed to attain rt. The reaction mixture was stirred over night before

being quenched with 6% (aq.) $KHSO_4$ and washed four times with 6% $KHSO_4$ (aq.), dried with Na_2SO_4, filtered and concentrated. Used without further purification. ^{1}H NMR (400 MHz, $CDCl_3$) δ 1.78 (s, 6H), 5.66 (s, 2H), 6.82 (d, *J* = 7.55Hz, 1H), 7.33-7.40 (m, 1H), 7.47-7.56 (m, 3H), 7.79-7.86 (m, 1H), 8.33-8.40 (m, 1H). ^{13}C NMR (100 MHz, $CDCl_3$) δ 26.7 (2C), 67.4, 90.3, 105.1, 105.9, 121.4, 121.8, 125.3, 125.4, 125.5, 126.6, 127.4, 134.4, 153.3, 159.9, 170.2, 191.8.

Ethyl 3-(7-(diethylamino)-2-oxo-2H-chromen-4-yl)propanoate (17). 1,1,1,3,3,3-Hexamethyldisilazane (11.25 mmol, 2.39 mL) was dissolved in dry THF (10 mL) and cooled to -20 °C. *n*BuLi (11.25 mmol, 0.97 mL) was added and the reaction was stirred for 15min. DMPU (11.25 mmol, 0.97 mL) was added and the reaction was warmed to 0 °C for 30min. The reaction mixture was again cooled to -20 °C and **14b** (8.65 mmol, 2 g) dissolved in dry THF (5 mL) was added over a 5min period and the reaction was allowed to stirr for 30 min. Ethyl bromoacetate (12.98 mmol, 1.44 mL) was added and the reaction was stirred for 30 min before quenching with saturated $NaHCO_3$ (aq). The reaction mixture was extracted with EtOAc (2x100mL), the combined organic phases was dried (Na_2SO_4), filtered, and concentrated. The crude product was purified with column chromatography on silica gel (heptane:EtOAc 80:20 to 70:30) to give **17** as a yellow oil (2.12g, 77% yield). ^{1}H-NMR (400MHz, $CDCl_3$) δ 7.38 (d, J = 9Hz, 1H), 6.55 (dd, J = 9.0, 2.5 Hz, 1H), 6.45 (d, J = 2.5 Hz, 1H), 5.88-5.89 (m, 1H), 4.13 (q, 2H), 3.37 (q, 4H), 3.01-2.95 (m, 2H), 2.67-2.61 (m, 2H), 1.23 (t, 3H), 1.17 (t, 6H). ^{13}C-NMR (100MHz, $CDCl_3$)δ 172.1, 162.1, 156.3, 154.8, 150.6, 125.1, 108.6, 107.8, 107.5, 97.8, 60.9, 44.7 (2C), 32.7, 26.5, 14.2, 12.5 (2C).

(R)-Methyl 2-(3-(7-(diethylamino)-2-oxo-2H-chromen-4-yl)propanamido)-3-(tritylthio)propanoate (19). 17 (0.26 mmol, 83 mg) was dissolved in THF (2 mL) before LiOH (0.31 mmol, 1 M aq, 0.31 mL) was added and the reaction was stirred at rt for 16h. The reaction mixture was neutralized with 1 M HCl (0.31 mL) and the solvent was evaporated. DMF (3 mL), HBTU (0.31 mmol, 118 mg) and DIPEA (0.65 mmol, 0.11 mL) was added and the reaction was stirred at rt for 1h. **18** (0.26 mmol, 100 mg) dissolved in DMF (3 mL) was added and the reaction was stirred at rt for 16h. The reaction mixture was diluted with water and pH was set to 8 with saturated $NaHCO_3$ (aq). The water phase was extracted with EtOAc (3x50mL) and the combined organic phases were washed with water (100mL). The organic phase was dried (Na_2SO_4), filtered, and concentrated. The crude product was purified with column chromatography on silica gel (heptane:EtOAc 60:40 to 50:50) to give **19** as a pale yellow foam (100 mg, 60% yield) $[\alpha]_D$ 12 (c 0.5, $CHCl_3$). ^{1}H-NMR (400MHz, d_6-DMSO)δ 8.49 (d, J = 7.8Hz, 1H), 7.49 (d, J = 9.1Hz, 1H), 7.34-7.19 (m, 15H), 6.65 (dd, J = 2.5, 9.1Hz, 1H), 6.48 (d, J = 2.5Hz, 1H), 5.86 (s, 1H), 4.18-4.10 (m, 1H), 3.52 (s, 3H), 3.38 (q, 4H), 2.90 (t, 2H), 2.57-2.46 (m, 3H), 2.39 (dd, J = 5.6, 12.4Hz, 1H), 1.09 (t, 6H). ^{13}C-NMR (100MHz, d_6-DMSO)δ 170.9, 170.7, 160.9, 155.8 (2C), 150.3, 144.1 (3C), 129.1 (6C), 128.1 (6C), 126.9 (3C), 125.7, 108.7, 107.3, 106.5, 96.9, 66.4, 52.1, 51.5, 44.0 (2C), 33.0, 32.8, 26.3, 12.4 (2C).

(R)-Methyl 2-(2-(7-(diethylamino)-2-oxo-2H-chromen-4-yl)ethyl)-4,5-dihydrothiazole-4-carboxylate (20). 19 (0.78 g, 1.2 mmol) was dissolved in DCM (25 mL) and cooled to 0 °C with an icebath and freshly distilled $TiCl_4$ (0.4 mL, 3.6 mmol) was added. The reaction was stirred over night (16 h, 0 °C→ rt). The reaction was quenched with saturated $NaHCO_3$ (aq) and extracted with DCM (2x150 mL), the combined organic phases was dried (Na_2SO_4), filtered and concentrated. The crude product was purified with column chromatography on silica gel (heptane:EtOAc 50:50 to 20:80) to give **20** as a yellow oil (187 mg, 40%) $[\alpha]_D$ 30 (c 0.5, $CHCl_3$). ^{1}H-NMR (400MHz, d_6-DMSO)δ 7.51 (d, J = 9.1Hz, 1H), 6.69 (dd, J = 2.4, 9.1Hz, 1H), 6.51 (d, J = 2.4, 1H), 5.92 (s, 1H), 5.17-5.10 (m, 1H), 3.68 (s, 3H), 3.60 (dd, J = 9.8, 11.2, 1H), 3.49 (dd, J = 8.2, 11.2, 1H), 3.42 (q, 4H), 3.03 (t, 2H), 2.86 (t, 2H), 1.12 (t, 6H). ^{13}C-NMR (100MHz, d_6-DMSO)δ 171.3, 170.8, 160.7, 155.8, 154.9, 150.3, 125.6, 108.7, 107.1, 106.9, 96.9, 77.5, 52.2, 43.9 (2C), 35.2, 31.8, 28.1, 12.3 (2C).

(3R)-Methyl 8-((7-(diethylamino)-2-oxo-2H-chromen-4-yl)methyl)-7-((naphthalen-1-yloxy)methyl)-5-oxo-3,5-dihydro-2H-thiazolo[3,2-a]pyridine-3-carboxylate (22). **20** (0.48 mmol, 187 mg), **21** (1.45 mmol, 486 mg), and TFA (0.96 mmol, 74 μl) was dissolved in DCE (3 mL) and the reaction was heated by microwave irradiation at 120 °C for 3 min. The reaction mixture was diluted with DCM and washed with saturated $NaHCO_3$ (aq). The organic phase was dried (Na_2SO_4), filtered, and concentrated. The crude product was purified with column chromatography on silica gel (heptane:EtOAc 20:80 to 0:100) to give **22** as a pale yellow solid (0.21mg, 73% yield) $[\alpha]_D$ = -5 (*c* 0.5, $CHCl_3$). ^{1}H-NMR (400MHz, [D$_6$]DMSO) *δ* 7.99 (d, J = 8.4Hz, 1H), 7.82 (d, J = 8.1Hz, 1H), 7.60 (d, J = 8.8Hz, 1H), 7.52-7.31 (m, 4H), 6.96 (d, J = 7.6Hz, 1H), 6.63 (dd, J = 9.1, 2.4Hz, 1H), 6.51 (s, 1H), 6.45 (d, J = 2.4Hz, 1H), 5.66 (dd, J = 9.0, 2.0Hz, 1H), 5.47 (s, 1H), 5.19-5.08 (m, 2H), 4.06-3.90 (m, 3H), 3.75 (s, 3H), 3.65 (dd, J = 11.9, 2.0Hz, 1H), 3.40 (q, 4H), 1.11 (t, 6H). ^{13}C-NMR (100MHz, [D$_6$]DMSO)*δ* 168.6, 160.8, 160.0, 155.6, 153.0, 152.9, 150.4, 150.3, 149.5, 133.9, 127.4, 126.4, 125.8, 125.5, 125.3, 124.6, 121.1, 120.5, 113.1, 108.5, 107.3, 105.6, 105.4, 105.3, 96.7, 66.4, 63.2, 52.9, 44.0 (2C), 31.2, 30.6,

12.3 (2C); Elemental analysis calcd (%) for $C_{34}H_{32}N_2O_6S$: C 68.44, H 5.41, N 4.69; found: C 66.9, H 5.4, N 4.65.

(3R)-8-((7-(Diethylamino)-2-oxo-2H-chromen-4-yl)methyl)-7-((naphthalen-1-yloxy)methyl)-5-oxo-3,5-dihydro-2H-thiazolo[3,2-a]pyridine-3-carboxylic acid (23). 22 (0.34 mmol, 0.2 g) was dissolved in THF (20 mL) and LiOH (0.44 mmol, 0.1M, 4.4 mL) was added, the reaction was stirred at rt for 1h. The solvent was evaporated and the crude product was purified with column chromatography on silica gel (first pure EtOAc then DCM:MeOH:AcOH 90:5:5) to give **23** as a yellow solid (180 mg, 91% yield) $[\alpha]_D$ = -10 (*c* 0.5, $CHCl_3$). ^{1}H-NMR (400MHz, $[D_6]$DMSO) δ 7.99 (d, J = 8.5 Hz, 1H), 7.82 (d, J = 8.1 Hz, 1H), 7.60 (d, J = 9.1 Hz, 1H), 7.52-7.30 (m, 4H), 6.96 (d, J = 7.7 Hz, 1H), 6.62 (dd, J = 9.2, 2.4 Hz, 1H), 6.48 (s, 1H), 6.44 (d, J = 2.4 Hz, 1H), 5.55-5.49 (m, 1H), 5.47 (s, 1H), 5.17-5.06 (m, 2H), 4.05-3.87 (m, 3H), 3.65-3.58 (m, 1H), 3.45-3.34 (m, 4H), 1.10 (t, 6H). ^{13}C-NMR (100MHz, $[D_6]$DMSO) δ 169.4, 160.8, 160.1, 155.6, 153.1, 153.0, 150.3, 150.1, 149.5, 133.9, 127.4, 126.4, 125.8, 125.5, 125.3, 124.7, 121.1, 120.5, 113.2, 108.5, 107.3, 105.6, 105.4, 105.2, 96.8, 66.5, 63.3, 44.0 (2C), 31.6, 30.6, 12.3 (2C). HRMS (ES+) calcd $[M+H^+]$ $C_{33}H_{30}N_2O_6S$, 583.1903, obsd 583.1900.

3-(1,3,5,7-Tetramethyl-4,4-difluoro-4-bora-3a,4a-diaza-*s*-indacene-8yl)-propionic Acid (24). 2,4-dimethyl pyrrole (1.3 mL, 10.0 mmol), TEA (1.68 mL, 12 mmol), and BF_3•OEt_2 (1.52 mL, 12 mmol) was added dropwise to succinic anhydride (1.0 g, 10 mmol) in dichloroethane (15 mL) while stirring. The reaction mixture was heated in a sealed tube by MWI 60 min, 120 °C. 2,4-dimethyl pyrrole (1.3 mL, 10.0 mmol), TEA (1.68 mL, 12 mmol), and BF_3•OEt_2 (1.52 mL, 12 mmol) was added and the reaction was again heated in a sealed tube by MWI 60 min, 120 °C. A final addition of 2,4-dimethyl pyrrole (1.3 mL, 10.0 mmol), TEA (1.68 mL, 12 mmol), and BF_3•OEt_2 (1.52 mL, 12 mmol) was perfomred and the reaction was again heated in a sealed tube by MWI 60 min, 120 °C. The reaction mixture was diluted with CH_2Cl_2 and washed three times with 2% $KHSO_4$ (aq.). The combined organic phases were dried over Na_2SO_4, filtrated and concentrated. Purification by column chromatography (heptane:EtOAc 3:1 to 3:2 + 0.5% AcOH) gave **24** as dark red solid (512 mg, 16% yield). IR (neat) 1702, 1548, 1509, 1201, 1066, 973 cm^{-1}; ^{1}H NMR (400 MHz, $CDCl_3$) δ 2.44 (s, 6H), 2.52 (s, 6H), 2.62-2.73 (m, 2H), 3.27-3.39 (m, 2H), 6.07 (s, 2H); ^{13}C NMR (100 MHz, $CDCl_3$) δ 14.5, 16.3, 23.4, 35.2, 122.0, 131.2, 140.4, 142.8, 154.8, 177.3. MS (electrospray ionization) calcd for [M + H] $C_{16}H_{20}BF_2N_2O_2$ 321, obsd 321.

(2R)-Methyl 2-(3-(1,3,5,7-tetramethyl-4,4-difluoro-4-bora-3a,4a-diaza-*s*-indacene-8-yl)propanamido)-3-mercaptopropanoate (25). Oxalyl chloride (45 µl, 0.53 mmol) was added dropwise to **24** (85 mg, 0.27 mmol) in 5 mL dry CH_2Cl_2 while stirring at rt. The reaction mixture was left to stir over night (~17h) and was then concentrated to give a dark brown-red solid, which was co-concentrated three times from dry CH_2Cl_2. The crude product was used in the next step without further purification.

TEA (74 µl, 0.53 mmol) followed by the acid chloride (0.27 mmol) in 1.5 mL dry CH_2Cl_2 was added dropwise to cysteine methyl ester (46 mg, 0.27 mmol) in 1 mL dry CH_2Cl_2 cooled on ice while stirring. The reaction mixture was stirred on ice for 30 min before being allowed to attain rt and stirring was continued for 4h. The reaction mixture was diluted with EtOAc and washed with brine. The aqueous layer was re-extracted three times with EtOAc and the combined organic layers were concentrated to give a dark red-brown solid. Purification by column chromatography (heptane:EtOAc 1:1) gave **25** as a red solid (75 mg, 64% yield). $[\alpha]_D$ = 29 (c 0.45, $CHCl_3$); IR (neat) 3316, 1743, 1658, 1548, 1508, 1200, 1159, and 983 cm^{-1}; ^{1}H NMR (400 MHz, $CDCl_3$) δ 1.29 (t, J = 9.02 Hz, 1H), 2.45 (s, 6H), 2.50 (s, 6H), 2.52-2.62 (m, 2H), 2.92-3.08 (m, 2H), 3.26-3.40 (m, 2H), 3.79 (s, 3H), 4.85-4.91 (m, 1H), 6.07 (s, 2H), 6.36 (d, J = 7.81 Hz, 1H); ^{13}C NMR (100 MHz, $CDCl_3$) δ 14.4, 16.4, 23.6, 26.7, 37.0, 52.8, 53.6, 121.9, 131.2, 140.4, 143.9, 154.5, 170.3, 170.4. MS (electrospray ionization) calcd for [M + H] $C_{20}H_{27}BF_2N_3O_3S$ 438, obsd 438.

(4R)-Methyl 2-(2-(1,3,5,7-tetramethyl-4,4-difluoro-4-bora-3a,4a-diaza-*s*-indacene-8-yl)ethyl)-4,5-dihydrothiazole-4-carboxylate (26). $TiCl_4$ (12 µl, 0.11 mmol) was added dropwise to **25** (37mg, 0.08 mmol) in 3 mL dry CH_2Cl_2 while stirring at 0 °C. Upon this addition the reaction mixture turned deep purple. The reaction mixture was left to stir at 0 °C for 1h before being allowed to attain rt. After a total of 3.5h the reaction was quenched with cold $NaHCO_3$ (aq. saturated) and the aqueous layer was extracted three times with CH_2Cl_2 and three times with EtOAc. The combined organic layers were dried over Na_2SO_4, filtered and concentrated. The crude product was dissolved in 5 mL dry CH_2Cl_2 and BF_3•OEt_2 (7 µl, 0.06mmol) was added at rt while stirring. The reaction mixture was left to stir for 40 min then diluted with CH_2Cl_2 and washed with water. The aqueous layer was extracted three times with CH_2Cl_2 and the combined organic layers were concentrated. Purification by column chromatography (H:E 3:2 to H:E 2:3) gave **26** as dark orange solid (27 mg, 75% yield). $[\alpha]_D$ = 33 (c 0,5, $CHCl_3$); IR (neat) 1741, 1548, 1509, 1197, and 981 cm^{-1}; ^{1}H NMR (400 MHz, $CDCl_3$) δ 2.44 (s, 6H), 2.51 (s, 6H), 2.77-2.86 (m, 2H), 3.30-3.44 (m, 2H), 3.57 (dd, J_1 = 9.74 Hz, J_2 = 11.27 Hz, 2H), 3.65 (dd, J_1 = 8.05 Hz, J_2 = 11.27 Hz, 2H) 3.81 (s, 3H), 5.11-5.18 (m, 1H), 6.06 (s, 2H); ^{13}C NMR (100 MHz, $CDCl_3$) δ 14.4, 14.5, 16.5,

25.7, 35.5, 36.0, 52.7, 77.7, 121.9, 131.2, 140.5, 143.3, 154.6, 171.0, 172.2. MS (electrospray ionization) calcd for [M + H] $C_{20}H_{25}BF_2N_3O_2S$ 420, obsd 420.

(3R)-Methyl 7-((naphthalen-1-yloxy)methyl)-5-oxo-8-((1,3,5,7-tetramethyl-4,4-difluoro-4-bora-3a,4a-diaza-s-indacene-8-yl)methyl)-3,5-dihydro-2H-thiazolo[3,2-a]pyridine-3-carboxylate (27). Meldrum's acid derivative **21** (79 mg, 0.24 mmol) followed by TFA (6 µl, 0.08 mmol) was added to **26** (34 mg, 0.08 mmol) in 0.5 mL DCE while stirring. The reaction mixture was heated in a sealed tube by MWI 140 s, 120 °C. A second addition of **21** (79 mg, 0.24 mmol) followed by TFA (6 µl, 0.08 mmol) was made and the reaction mixture was again heated by MWI 140 s, 120 °C. For all starting material to be consumed a third addition of **21** (40 mg, 0.12 mmol) was required. The reaction mixture was heated for 3min, 120 °C by MWI before being diluted with CH_2Cl_2 and washed with $NaHCO_3$ (aq. saturated). The aqueous layer was extracted three times with CH_2Cl_2 and the combined organic layers were dried over Na_2SO_4, filtered and concentrated. Purification by column chromatography (H:E 2:3) gave **27** as a dark pink solid (32 mg, 64% yield). $[\alpha]_D$ = -38 (*c* 0.2, $CHCl_3$); ^{1}H NMR (400 MHz, $CDCl_3$) δ 2.22 (br s, 6H), 2.53 (s, 6H), 3.33 (dd, J_1 = 1.59 Hz, J_2 = 11.79 Hz, 1H), 3.50 (dd, J_1 = 8.40 Hz, J_2= 11.73 Hz, 1H) 3.75 (s, 3H), 4.12-4.27 (m, 2H), 5.00-5.10 (m, 2H) 5.59 (dd, J_1 = 1.76 Hz, J_2 = 8.42 Hz, 1H), 6.01 (s, 2H), 6.77 (s, 1H), 6.80 (d, J = 7.57 Hz, 1H), 7.33-7.39 (m, 1H), 7.46-7.55 (m, 3H), 7.79-7.84 (m, 1H), 8.25-8.31 (m, 1H); ^{13}C NMR (100 MHz, $CDCl_3$) δ 14.6, 15.5-16.0 (m), 28.6, 32.4, 53.2, 61.9, 67.2, 105.1, 106.1, 155.5, 121.4, 121.8, 121.9, 125.3, 125.6, 126.7, 133.7, 134.6, 137.1, 141.0-141.6 (broad), 144.9, 149.6, 153.4, 155.3-155.9 (broad), 161.0, 168.1; IR ν 1752, 1664, 1552, 1506, 1195, 1159, and 985 cm^{-1}; MS (electrospray ionization) calcd for [M + H] $C_{34}H_{33}BF_2N_3O_4S$ 628, obsd 628. Elemental analysis calcd (%) for $C_{34}H_{32}BF_2N_3O_4S$: C 65.08, H 5.14, N 6.70; found: C 63.75, H 5.15, N 6.5.

7-((Naphthalen-1-yloxy)methyl)-5-oxo-8-((1,3,5,7-tetramethyl-4,4-difluoro-4-bora-3a,4a-diaza-s-indacene-8-yl)methyl)-3,5-dihydro-2H-thiazolo[3,2-a]pyridine-3-carboxylic acid (28). LiI (111 mg, 0.83 mmol) was added to **27** (52 mg, 0.08 mmol) in 2 mL dry pyridine while stirring. The reaction mixture was heated to 140°C for 15 min by microwave irradiation. The reaction mixture was allowed to attain rt, diluted with CH_2Cl_2 and washed with 2% $KHSO_4$. The aqueous layer was extracted three times with CH_2Cl_2 and the combined organic layers were dried over Na_2SO_4, filtered and concentrated in vaccuo. The resulting dark oil was diluted in 2 mL 1,2-dichloroethane and TEA (58 µl, 0.42 mmol) was added dropwise while stirring. After 5 min BF_3•OEt_2 was added and the reaction mixture was heated to 80°C for 15 min. After being allowed to attain rt the reaction mixture was diluted with CH_2Cl_2 and washed with water. The aqueous layer was extracted five times with CH_2Cl_2 and the combined organic layers were concentrated in vaccuo. Purification by column chromatography (CH_2Cl_2, 2% MeOH → CH_2Cl_2, 2% MeOH, 1% AcOH) gave **28** as an orange solid (15 mg, 29%). $[\alpha]_D$ = 0; ^{1}H NMR (400 MHz, [D$_6$]DMSO) δ 2.21 (s, 3H), 2.29 (s, 3H), 2.43 (s, 6H), 3.39 (dd, J_1 = 1.26 Hz, J_2 = 11.88 Hz, 2H), 3.56 (dd, J_1 = 8.76 Hz, J_2 = 11.88 Hz, 2H), 4.27 (d, J = 16.73 Hz, 1H), 4.35 (d, J = 16.73 Hz, 1H), 5.36-5.40 (m, 3H), 6.18-6.24 (m, 2H), 6.58 (s, 1H), 7.17-7.23 (m, 1H), 7.43-7.49 (m, 1H), 7.53-7.61 (m, 3H), 7.89-7.95 (m, 1H), 8.24-8.29 (m, 1H). ^{13}C NMR (100 MHz, [D$_6$]DMSO) δ 14.7, 14.72, 15.3, 15.8, 28.53, 32.1, 62.0, 66.8, 105.1, 106.7, 113.4, 121.2, 121.7, 122.1, 122.1, 125.2, 126.2, 127.1, 128.1, 134.1 (broad), 134.6, 138.9, 142.4 (broad), 144.8, 150.6, 153.4, 154.5, 154.9 (broad), 160.5, 169.8; IR ν 1647, 1553, 1506, 1198, 1159, and 982 cm^{-1}; HRMS (electrospray ionization) calcd for [M + H] $C_{33}H_{31}BF_2N_3O_4S$ 614.2096, obsd 614.2097.

(3R)-Methyl 7-(naphthalen-1-ylmethyl)-5-oxo-8-(4-(1,3,5,7-tetramethyl-4,4-difluoro-4-bora-3a,4a-diaza-*s*-indacene-8-yl)phenyl)-3,5-dihydro-2H-thiazolo[3,2-a]pyridine-3-carboxylate (30). **29** (0.34 mmol, 0.16 g), oxalylchloride (0.34 mmol, 30 µl) and DMF (0.34 mmol, 26 µl) was mixed in DCM (16 mL) and the reaction was stirred at rt for 1h. The solvent was evaporated and the crude material was dissolved in dry DCE (16mL), BF_3•Et_2O (0.41 mmol, 52 µl), TEA (0.41 mmol, 57 µl) and 2,4-dimethyl-1H-pyrrole (0.85 mmol, 88 µl) was added and the reaction was heated in the microwave oven at 140 °C for 50 min. The solvent was evaporated and the crude product was purified with column chromatography on silica gel (heptane:EtOAc:AcOH 29:70:1) followed by HPLC, C18, 250x21.2 mm, 5 µm, 0-100% MeCN over 1h. The product was lyophilized to give **30** as a red solid (35 mg, 15% yield) $[\alpha]_D$ = -38 (*c* 0.05, Acetone). ^{1}H-NMR (400 MHz, [D$_6$]Acetone) δ 7.93-7.87 (m, 1H), 7.84-7.78 (m, 2H), 7.69-7.55 (m, 2H), 7.52-7.39 (m, 5H), 7.36-7.31 (m, 1H), 6.11 (s, 1H), 5.97 (s, 1H), 5.83 (s, 1H), 5.65 (dd, J = 9, 2.4 Hz, 1H), 4.17 (s, 2H), 3.96 (dd, J = 11.9, 9Hz, 1H), 3.78 (s, 3H), 3.60 (dd, J = 11.9, 2.4 Hz, 1H), 2.49 (s, 3H), 2.46 (s, 3H), 1.48 (s, 3H), 1.05 (s, 3H). ^{13}C-NMR (100MHz, [D$_6$]Acetone) δ 169.4, 161.4, 156.2, 156.1, 154.3, 149.1, 143.9, 143.8, 142.5, 138.5, 135.6, 135.2, 134.8, 132.6, 132.3 (2C), 132.2, 132.0, 129.6, 129.5, 128.3, 128.0, 127.1, 126.6, 126.3, 124.5, 122.1 (2C), 115.7, 115.4, 64.6, 53.3, 37.0, 32.2, 14.6 (3C), 14.2; Elemental analysis calcd (%) for $C_{39}H_{34}BF_2N_3O_3S$: C 69.54, H 5.09, N 6.24; found: C 68.5, H 5.2, N 6.25.

(3R)-7-(Naphthalen-1-ylmethyl)-5-oxo-8-(4-(1,3,5,7-tetramethyl-4,4-difluoro-4-bora-3a,4a-diaza-*s*-indacene-8-yl)phenyl)-3,5-dihydro-2H-thiazolo[3,2-a]pyridine-3-carboxylic acid (31). **30** (0.045 mmol, 30

mg) was dissolved in THF (5 mL) and LiOH (0.09 mmol, 0.1M, 0.9 mL) was added, the reaction was stirred at rt for 1h. The reaction mixture was diluted with water and pH was set to 1 with 1M HCl (aq). The waterphase was extracted with EtOAc (2x50 mL), the combined organic phases was dried (Na_2SO_4), filtered and concentrated. The crude product was purified with HPLC, (C18, 250x21.2 mm, 5µm, 0-100% MeCN over 1h) and lyophilized to give **31** as a red solid (25 mg, 84% yield) $[\alpha]_D$ = 20 (*c* 0.05, $CHCl_3$). ^{1}H-NMR (400MHz, $[D_6]$DMSO) δ 7.96-7.88 (m, 1H), 7.85-7.78 (m, 1H), 7.78-7.71 (m, 1H), 7.64-7.34 (m, 7H), 7.31-7.25 (m, 1H), 6.18 (s, 1H), 6.03 (s, 1H), 5.70 (s, 1H), 5.55-5.46 (m, 1H), 4.09 (s, 2H), 3.92-3.83 (m, 1H), 3.57-3.49 (m, 1H), 2.44 (s, 3H), 2.41 (s, 3H), 1.42 (s, 3H), 0.93 (s, 3H). ^{13}C-NMR (100MHz, $[D_6]$DMSO) δ 169.5, 160.0, 154.9 (2C), 152.8, 148.2, 142.6 (2C), 141.4, 137.2, 134.0, 133.8, 133.3, 131.2 (4C), 130.6, 128.6, 128.2 (2C), 127.3, 126.9, 126.3, 125.8, 125.5, 123.4, 121.5 (2C), 114.2, 113.9, 63.4, 35.5, 31.4, 14.2 (2C), 13.8, 13.3. HRMS (ES+) calcd $[M+H^+]$ $C_{38}H_{32}BF_2N_3O_3S$, 660.2304, obsd 660.2303.

5-(3-(1,3,5,7-Tetramethyl-4,4-difluoro-4-bora-3a,4a-diaza-*s*-indacene-8-yl)-1-hydroxypropylidene)-2,2-dimethyl-1,3-dioxane-4,6-dione (32). **24** (170 mg, 0.53 mmol) and DCC (131 mg, 0.64 mmol) was dissolved in DCM (4.2 mL) at 0 °C and stirred for 30 min before 2,2-dimethyl-1,3-dioxane-4,6-dione (84 mg, 0.58 mmol) and DMAP (122 mg, 0.85 mmol) was added and the solution was allowed to attain rt. The reaction mixture was stirred for 8h before being quenched with 2% (aq.) $KHSO_4$ and washed twice with 2% (aq.) $KHSO_4$, dried with Na_2SO_4, filtered and concentrated. Trituration with MeOH gave **32** as a red non crystalline solid (194 mg, 82%). ^{1}H NMR (400 MHz, $CDCl_3$) δ 1.7 (s, 6H), 2.39 (s, 6H), 2.49 (s, 6H), 3.29-3.39 (m, 2H), 3.40-3.50 (m, 2H), 6.04 (s, 2H), 15.71 (s, 1H). ^{13}C NMR (100 MHz, $CDCl_3$) δ 14.4, 16.2, 23.1, 26.7, 35.9, 91.6, 105.2, 122.0, 131.2, 140.4, 142.7, 154.6, 159.9, 170.5, 194.1. MS (electrospray ionization) calcd for [M - H] $C_{22}H_{24}BF_2N_2O_5$ 445, obsd 445.

(3R)-Methyl 5-oxo-8-phenyl-7-(2-(1,3,5,7-tetramethyl-4,4-difluoro-4-bora-3a,4a-diaza-*s*-indacene-8-yl)ethyl)-3,5-dihydro-2H-thiazolo[3,2-a]pyridine-3-carboxylate (33). **33** was synthesized by following the same procedure described for **10a** starting from **32** (170 mg, 0.38 mmol) and **9a** (45 mg, 0.19 mmol). Purification by column chromatography (heptane:ethylacetate 1:9 to heptane:ethylacetate:MeOH 5:90:5) gave **33** as a red foam (91 mg, 85%). $[\alpha]_D$ = 84 (*c* 0.1, $CHCl_3$); ^{1}H NMR (400 MHz, $CDCl_3$) δ 2.23 (s, 6H), 2.46 (s, 6H), 2.48-2.56 (m, 2H), 3.12-3.21 (m, 2H), 3.45 (dd, J_1= 2.33 Hz, J_2= 11.74 Hz, 1H), 3.65 (dd, J_1= 8.64 Hz, J_2= 11.70 Hz, 1H), 3.85 (s, 3H), 5.65 (dd, J_1= 2.30 Hz, J_2= 8.50 Hz, 1H), 5.99 (s, 2H), 6.36 (s, 1H), 7.11-7.20 (m, 2H), 7.30-7.42 (m, 3H); ^{13}C NMR (100 MHz, $CDCl_3$) δ 14.5 (m, 2C), 16.3 (2C), 25.9, 31.6, 33.8, 53.4, 63.6, 112.6, 115.8, 121.9 (broad), 128.8, 129.31, 129.33 129.6, 130.0, 131.3 (broad), 135.8, 140.3 (broad), 143.9, 147.3, 153.4, 154.5 (broad), 161.3, 168.4; IR ν 1753, 1656, 1549, 1508, 1484, 1198, 1157, 981, and 704 cm^{-1}; MS (electrospray ionization) calcd for [M + H] $C_{30}H_{31}BF_2N_3O_3S$ 562, obsd 562. Elemental analysis calcd (%) for $C_{30}H_{30}BF_2N_3O_3S$: C 64.18, H 5.39, N 7.48; found: C 63.65, H 5.3, N 7.3.

(3R)-5-Oxo-8-phenyl-7-(2-(1,3,5,7-tetramethyl-4,4-difluoro-4-bora-3a,4a-diaza-*s*-indacene-8-yl)ethyl)-3,5-dihydro-2H-thiazolo[3,2-a]pyridine-3-carboxylic acid (34). **34** was synthesized by following the same procedure described for **11a** starting from **33** (30 mg, 0.053 mmol). Purification by column chromatography (CH_2Cl_2:MeOH 97:3 to CH_2Cl_2:MeOH:AcOH 96:3:1) gave **34** as a red non-crystalline solid (25.5 mg, 88%). $[\alpha]_D$ = 34 (*c* 0.05, MeOH); ^{1}H NMR (400 MHz, $[D_6]$DMSO) δ 2.25 (s, 6H), 2.36 (s, 6H), 2.39-2.49 (m, 2H), 3.14-3.25 (m, 2H), 3.43-3.50 (m, 1H), 3.73-3.82 (m, 1H), 5.40-5.47 (m, 1H), 6.19 (s, 2H), 6.30 (s, 1H), 7.18-7.24 (m, 1H), 7.25-7.36 (m, 2H), 7.36-7.43 (m, 2H); ^{13}C NMR (100 MHz, $[D_6]$DMSO) δ 14.1 (m, 2C), 15.7 (2C), 25.5, 31.7, 33.4, 64.0, 111.5, 113.8, 121.8 (broad), 128.2, 129.0 (2C), 129.9 130.1, 130.5, 136.2, 140.9 (broad), 144.9, 147.9, 152.0, 153.6 (broad), 160.2, 169.5; IR ν 1636, 1548, 1509, 1489, 1407, 1198, 981, and 704 cm^{-1}; HRMS (electrospray ionization) calcd for [M + H] $C_{29}H_{29}BF_2N_3O_3S$ 548.1991, obsd 548.1972.

(3R)-Methyl 5-oxo-8-phenyl-7-(2-(1,3,5,7-tetramethyl-4,4-difluoro-4-bora-3a,4a-diaza-*s*-indacene-8-yl)ethyl)-3,5-dihydro-2H-oxazolo[3,2-a]pyridine-3-carboxylate (36). **36** was synthesized by following the previously published procedure[28] starting from **35** (60 mg, 0.25 mmol) and **32** (335 mg, 0.75 mmol). Purification by column chromatography (CH_2Cl_2:acetone 9:1) gave **36** as a red foam (100 mg, 73%). $[\alpha]_D$ = -44 (*c* 0.2, $CHCl_3$); ^{1}H NMR (400 MHz, $CDCl_3$) δ 2.22 (s, 6H), 2.46 (s, 6H), 2.57-2.63 (m, 2H), 3.09-3.23 (m, 2H), 3.86 (s, 3H), 4.63 (dd, J_1= 4.76 Hz, J_2= 9.36 Hz, 1H), 4.78 (t, J = 9.37 Hz, 1H), 5.26 (dd, J_1= 4.75 Hz, J_2= 9.38 Hz, 1H), 5.98 (s, 2H), 6.23 (s, 1H), 7.14-7.20 (m, 2H), 7.29-7.40 (m, 3H); ^{13}C NMR (100 MHz, $CDCl_3$) δ 14.3 (m, 2C), 16.2 (2C), 26.2, 33.7, 53.4, 57.0, 71.3, 99.7, 108.4, 121.7 (broad), 128.1, 128.8 (2C), 130.5 (2C), 131.2 (broad), 131.7, 140.3 (broad), 143.8, 153.7, 154.3 (broad), 156.0, 159.0, 168.0; IR ν 1753, 1671, 1547, 1507, 1195, 974, and 702 cm^{-1}; MS (electrospray ionization) calcd for [M + H] $C_{30}H_{31}BF_2N_3O_4$ 546, obsd 546. Elemental analysis calcd (%) for $C_{30}H_{30}BF_2N_3O_4$: C 66.07, H 5.54, N 7.70; found: C 65.3, H 5.6, N 7.6.

(3R)-5-Oxo-8-phenyl-7-(2-(1,3,5,7-tetramethyl-4,4-difluoro-4-bora-3a,4a-diaza-*s*-indacene-8-yl)ethyl)-3,5-dihydro-2H-oxazolo[3,2-a]pyridine-3-carboxylic acid (37). **37** was synthesized by following a previously published procedure,[28] starting from **36** (51 mg, 0.094 mmol). Purification by column chromatography (CH_2Cl_2:MeOH 97:3 to CH_2Cl_2:MeOH:AcOH 96:3:1) gave **37** as a red non-crystalline solid (27 mg, 54%). $[\alpha]_D$ = -26 (*c* 0.1, $CHCl_3$); ^{1}H NMR (400 MHz, [D$_6$]DMSO) δ 2.23 (bs, 6H), 2.35 (s, 6H), 2.44-2.68 (m, 2H), 3.05-3.29 (m, 2H), 4.58 (dd, J_1= 3.78 Hz, J_2= 8.92 Hz, 1H), 4.78 (t, J = 9.02 Hz, 1H), 4.96 (dd, J_1= 3.72 Hz, J_2= 9.21 Hz, 1H), 6.08 (s, 1H), 6.16 (s, 2H), 7.19-7.28 (m, 3H), 7.29-7.37 (m, 2H); ^{13}C NMR (100 MHz, [D$_6$]DMSO) δ 14.1 (m, 2C), 15.6 (2C), 26.0, 33.3, 58.3, 72.8, 97.8, 107.1, 121.8 (broad), 127.3, 128.4 (2C), 130.5 (2C), 130.8 (broad), 132.8, 140.8 (broad), 145.0, 153.6 (broad), 153.9, 154.4, 158.3, 169.6; IR ν 1659, 1549, 1510, 1197, 981, and 703 cm^{-1}; HRMS (electrospray ionization) calcd for [M + H] $C_{29}H_{29}BF_2N_3O_4$ 532.2219, obsd 532.2213.

1,5-Dioxo-8-phenyl-7-(2-(1,3,5,7-tetramethyl-4,4-difluoro-4-bora-3a,4a-diaza-*s*-indacene-8-yl)ethyl)-3,5-dihydro-2H-thiazolo[3,2-a]pyridine-3-carboxylic acid (38). *m*CPBA (70%, 9 mg, 0.37 mmol) was added to **34** (18.6 mg, 0.034 mmol) in 1.5 mL CH_2Cl_2 at rt. The solution was stirred for 15 min before quenching with $Na_2S_2O_5$ (aq. saturated). Extracted three times with CH_2Cl_2, dried with Na_2SO_4, filtered, and concentrated. Purification by column chromatography (CH_2Cl_2:acetone 9:1 to CH_2Cl_2:acetone:AcOH 88:10:2) gave **38** as a red foam (15.4 mg, 80% yield). $[\alpha]_D$ = -10 (*c* 0.1, DMSO); ^{1}H NMR (400 MHz, [D$_6$]DMSO) δ 2.26 (s, 6H), 2.39 (s, 6H), 2.54-2.64 (m, 2H), 3.14-3.32 (m, 2H), 3.53-3.62 (m, 2H), 5.40-5.49 (m, 1H), 6.02 (s, 2H), 6.79 (s, 1H), 7.30-7.49 (m, 5H); ^{13}C NMR (100 MHz, [D$_6$]DMSO) δ 14.1 (2C), 15.6 (2C), 25.4, 33.2, 51.0, 65.2, 119.9, 120.5, 121.9 (broad), 128.47, 128.54, 128.6 130.4, 130.5 (broad), 131.1, 133.4, 140.9 (broad), 144.6, 149.0, 151.8, 153.7 (broad), 159.0, 170.1; IR ν 1650, 1549, 1509, 1407, 1197, 981, and 704 cm^{-1}; HRMS (electrospray ionization) calcd for [M + H] $C_{29}H_{29}BF_2N_3O_4S$ 564.1940, obsd 564.1928.

^{1}H-NMR and ^{13}C-NMR spectra

^{1}H-NMR spectrum of compound **10a**

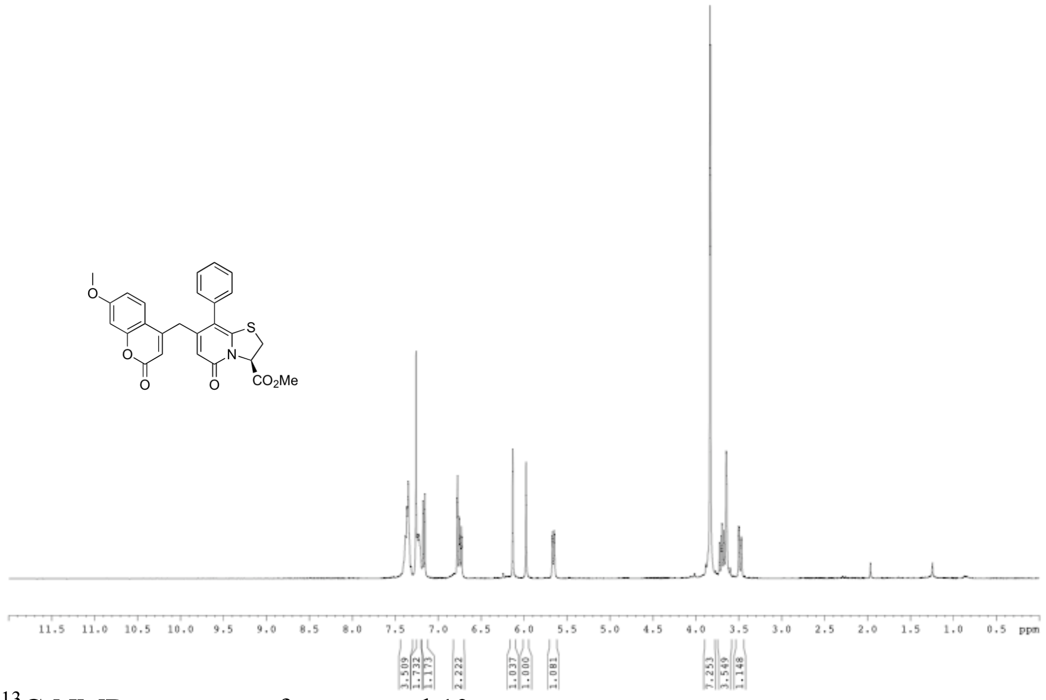

^{13}C-NMR spectrum of compound **10a**

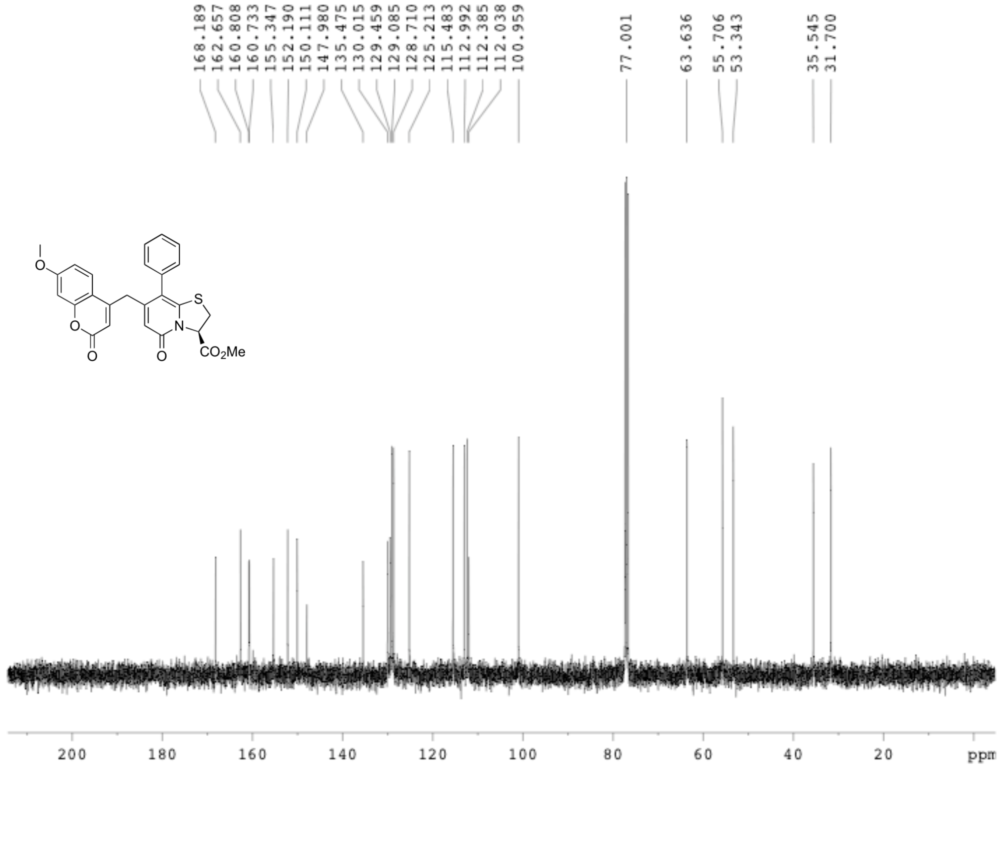

^{1}H-NMR spectrum of compound **10b**

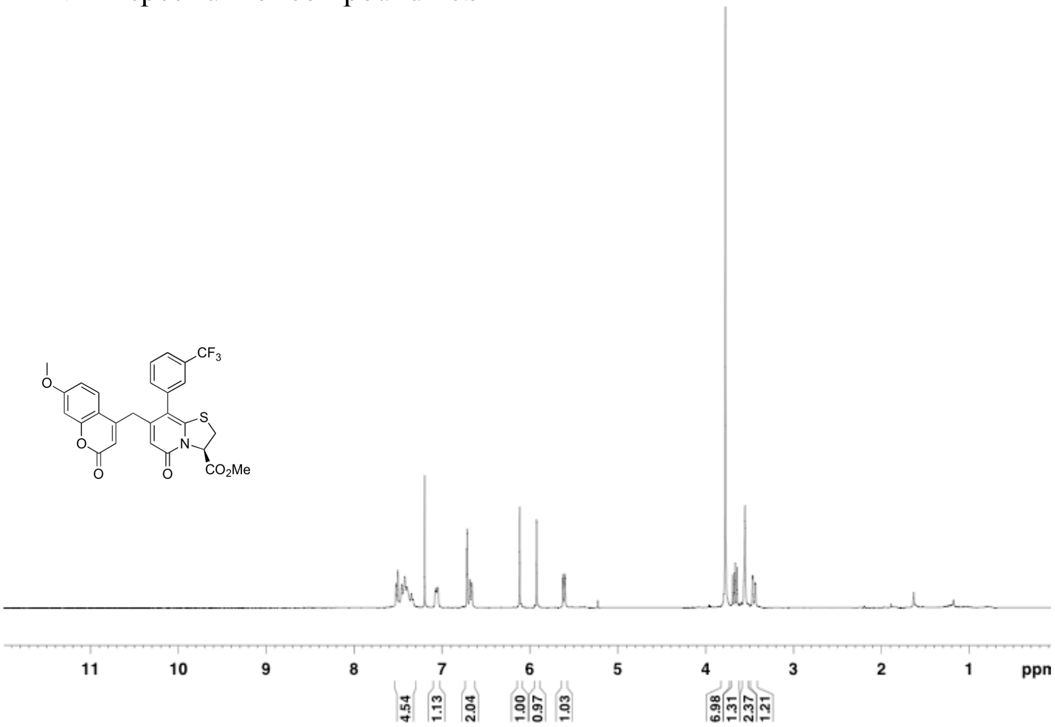

^{13}C-NMR spectrum of compound **10b**

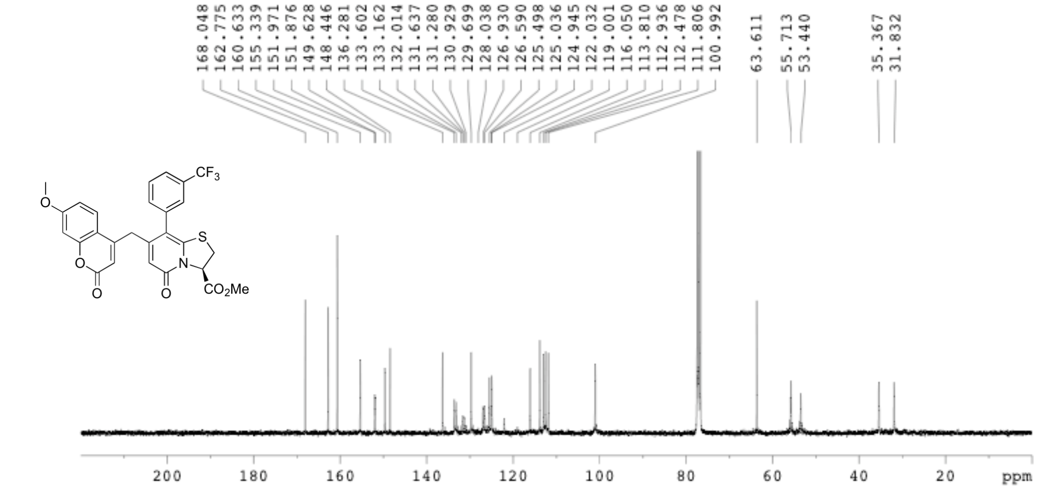

^{1}H-NMR spectrum of compound **10c**

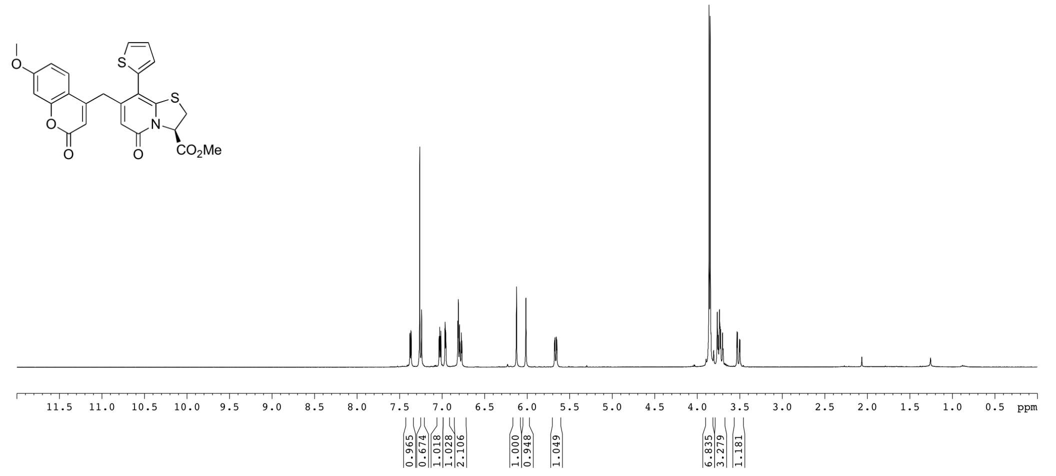

^{13}C-NMR spectrum of compound **10c**

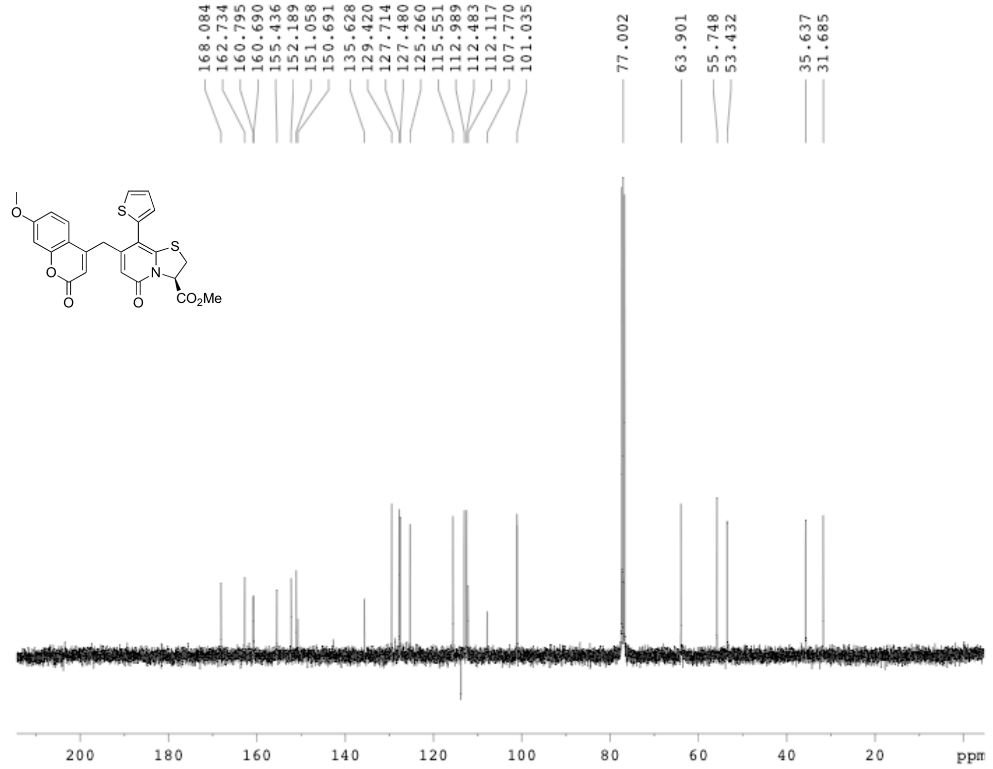

^{1}H-NMR spectrum of compound **10d**

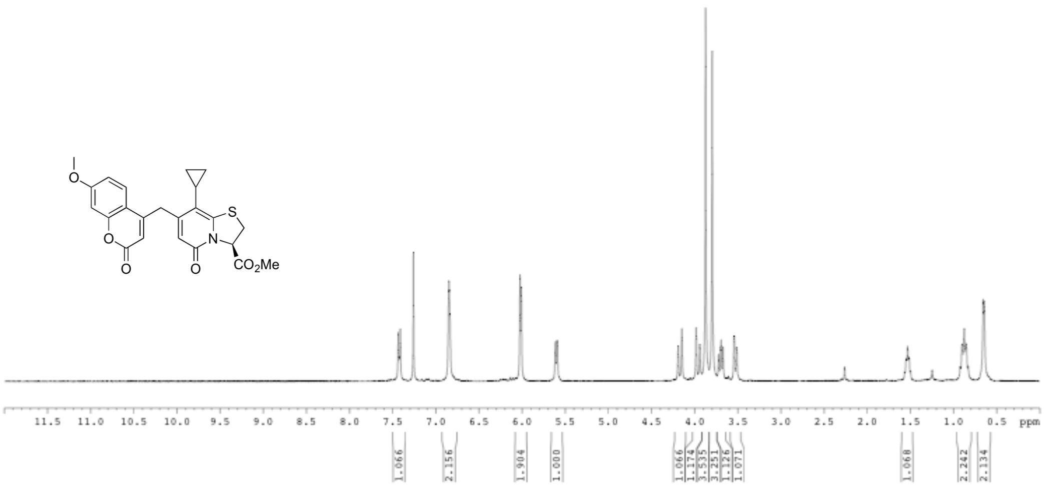

^{13}C-NMR spectrum of compound **10d**

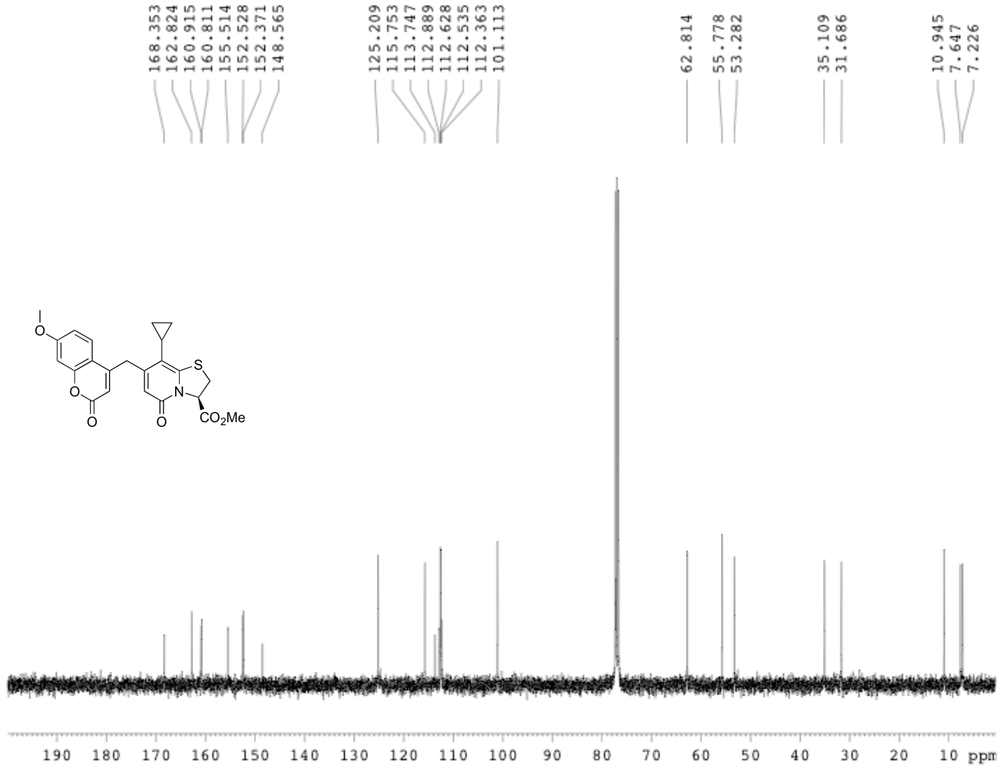

^{1}H-NMR spectrum of compound **11a**

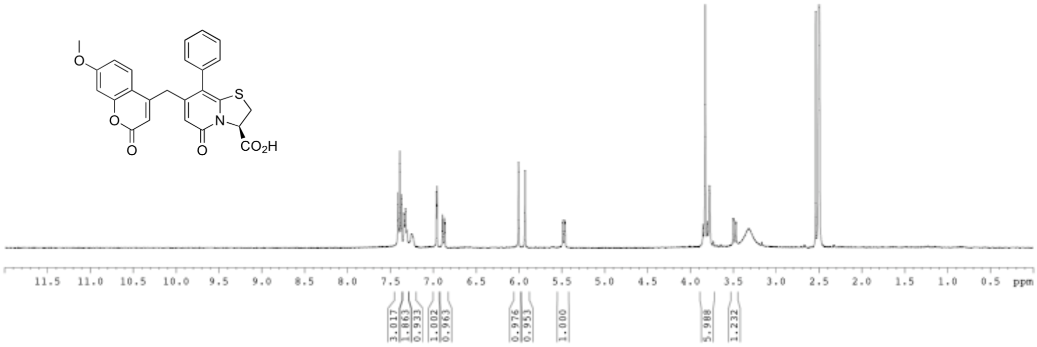

^{13}C-NMR spectrum of compound **11a**

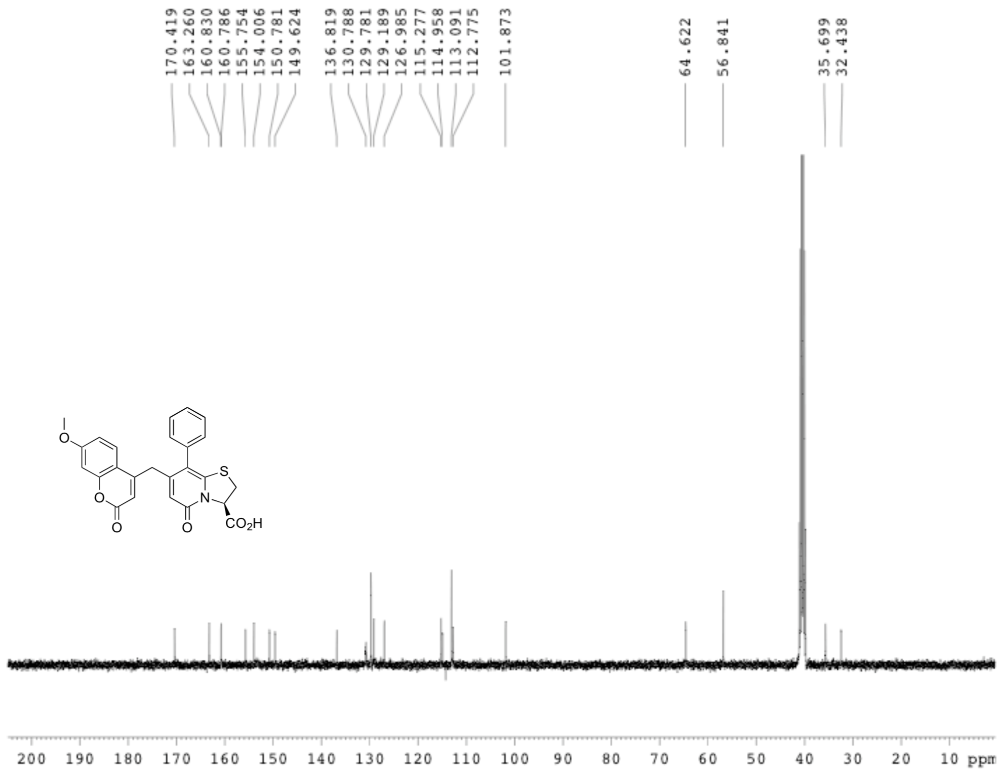

^{1}H-NMR spectrum of compound **11b**

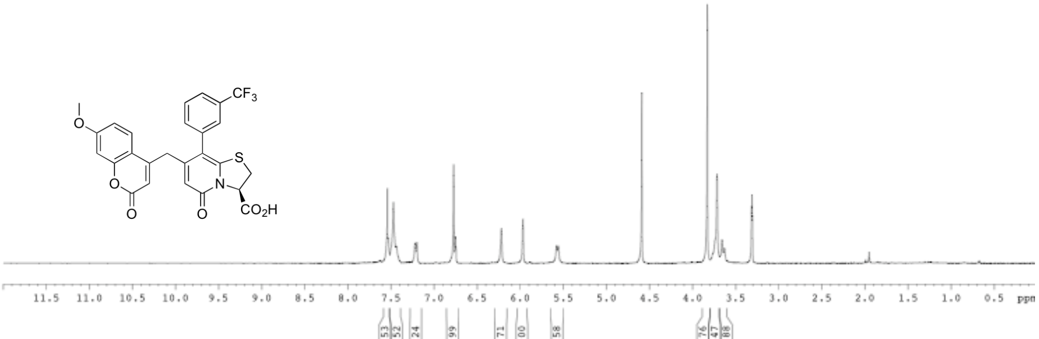

^{13}C-NMR spectrum of compound **11b**

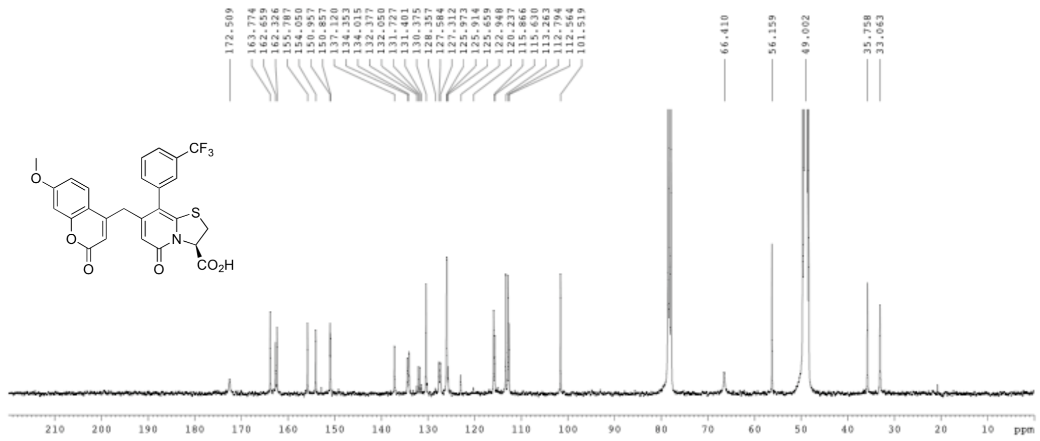

^{1}H-NMR spectrum of compound **11c**

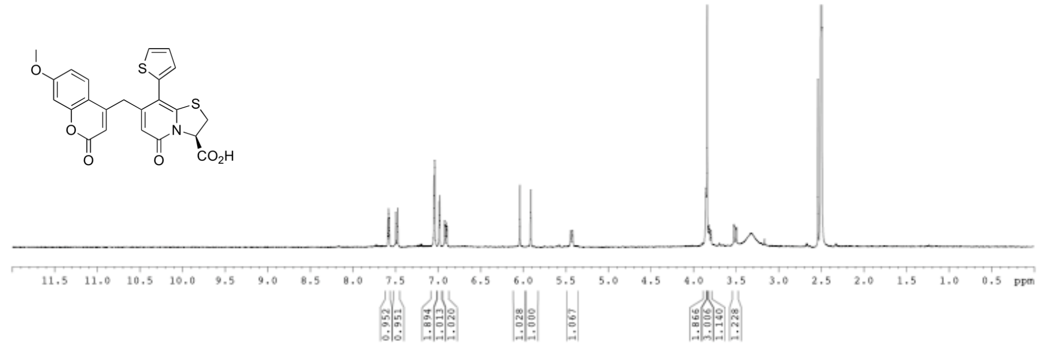

^{13}C-NMR spectrum of compound **11c**

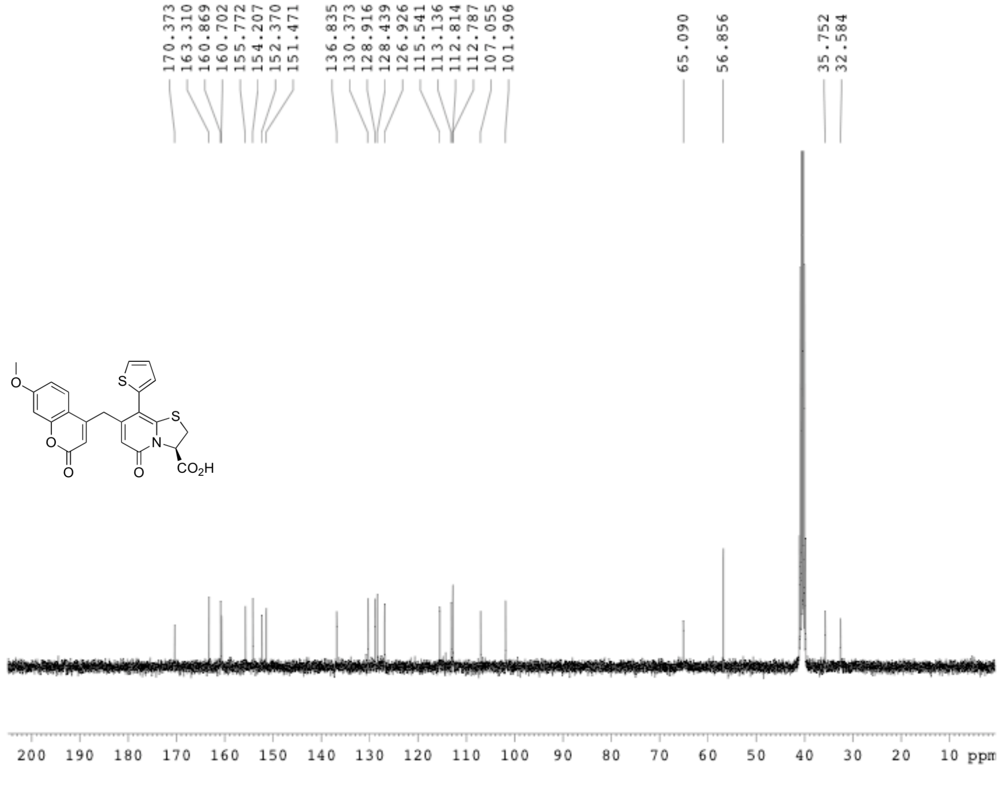

^{1}H-NMR spectrum of compound **11d**

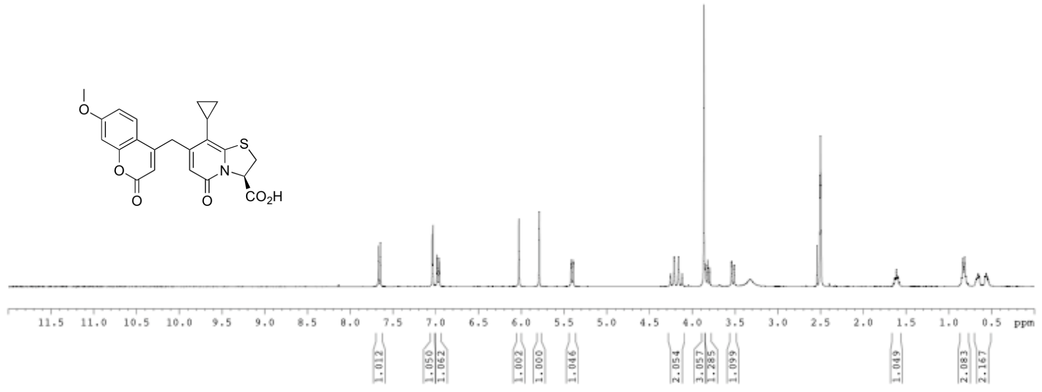

^{13}C-NMR spectrum of compound **11d**

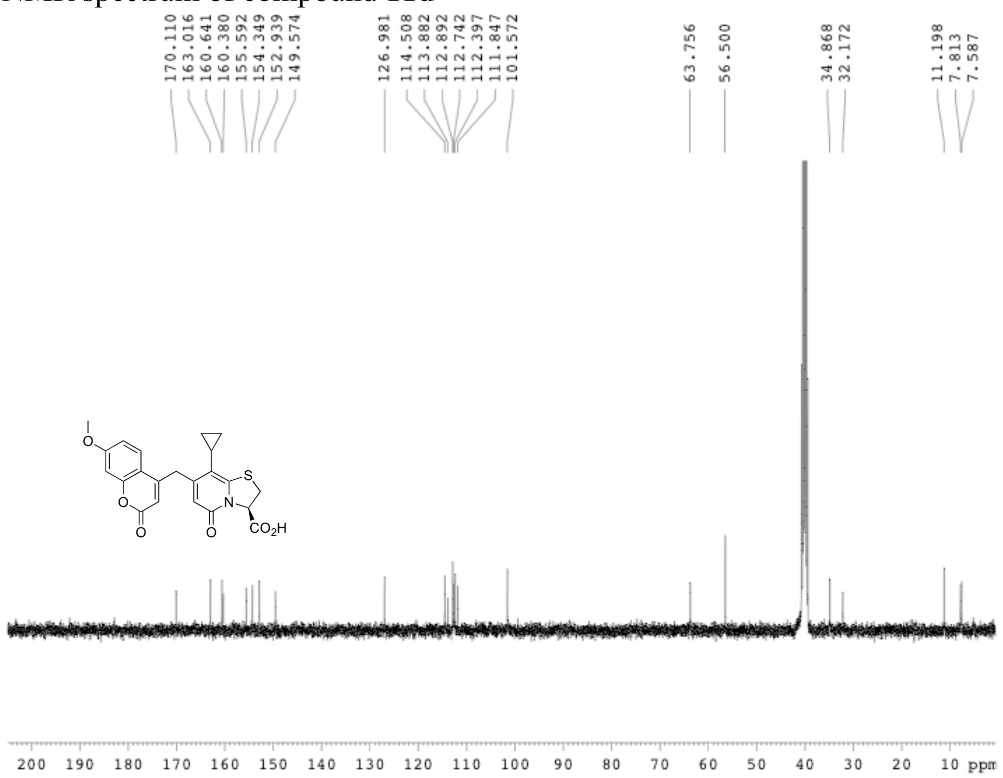

^{1}H-NMR spectrum of compound **15a**

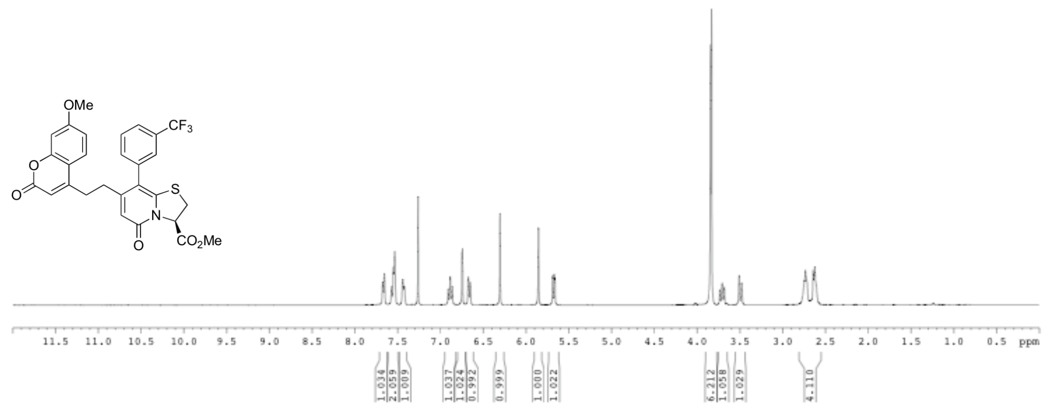

^{13}C-NMR spectrum of compound **15a**

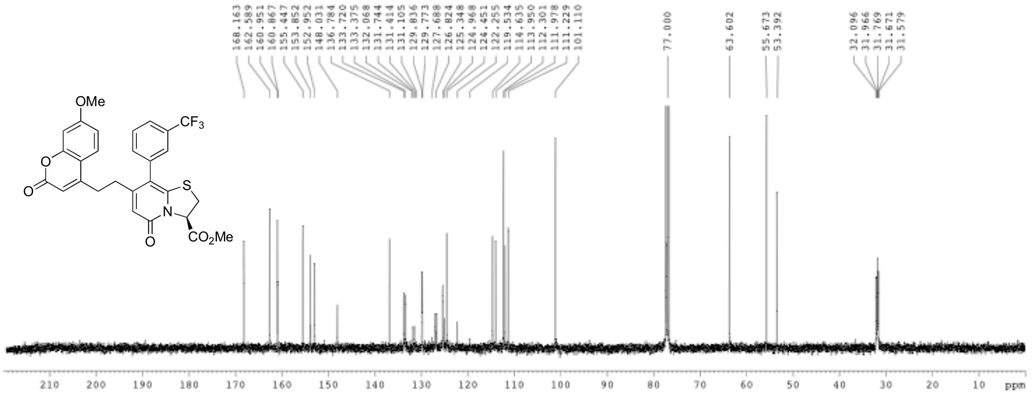

^{1}H-NMR spectrum of compound **15b**

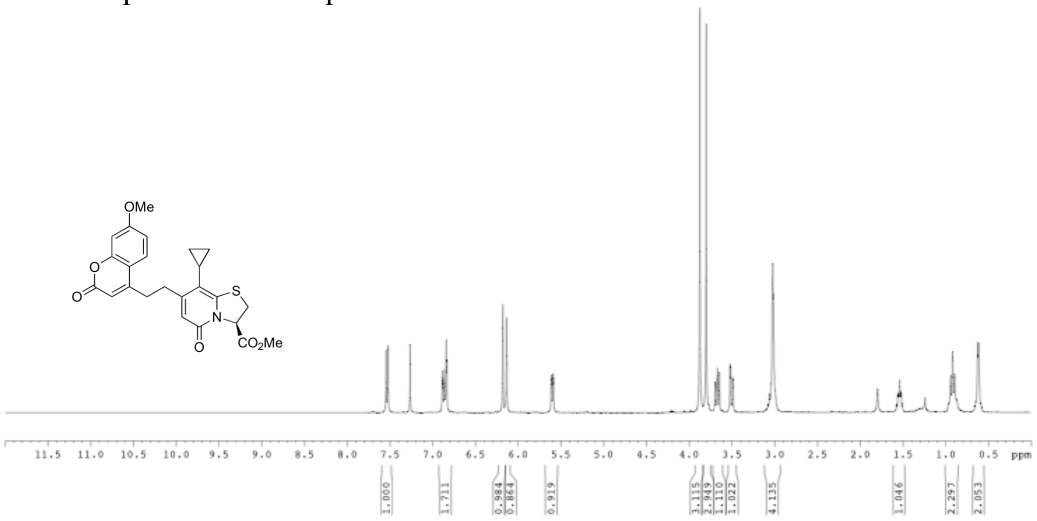

^{13}C-NMR spectrum of compound **15b**

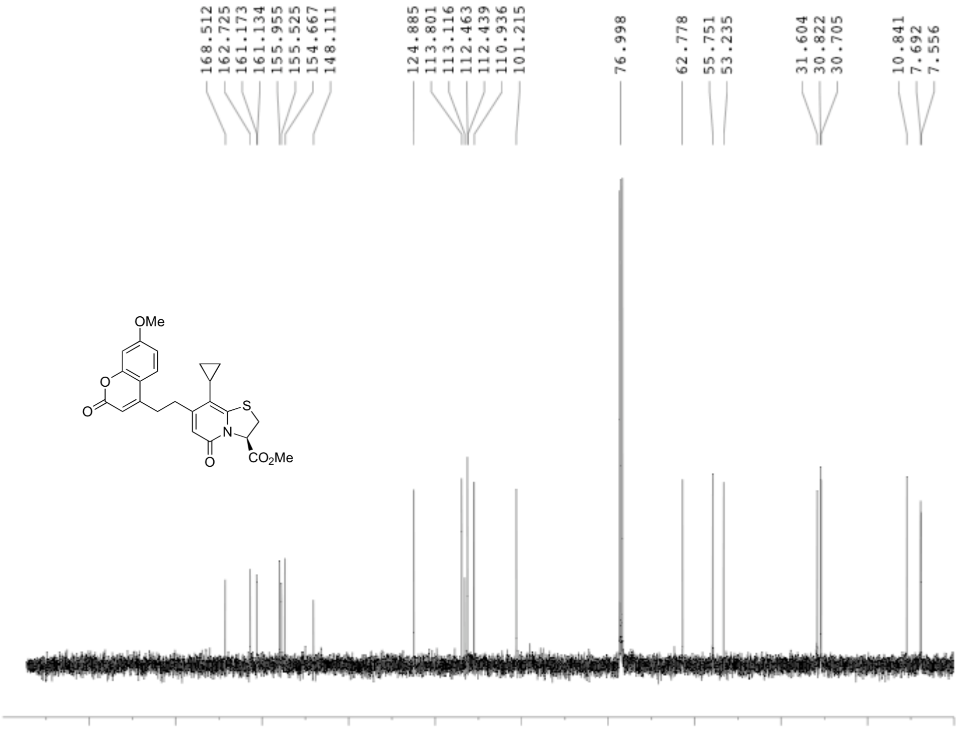

^{1}H-NMR spectrum of compound **15c**

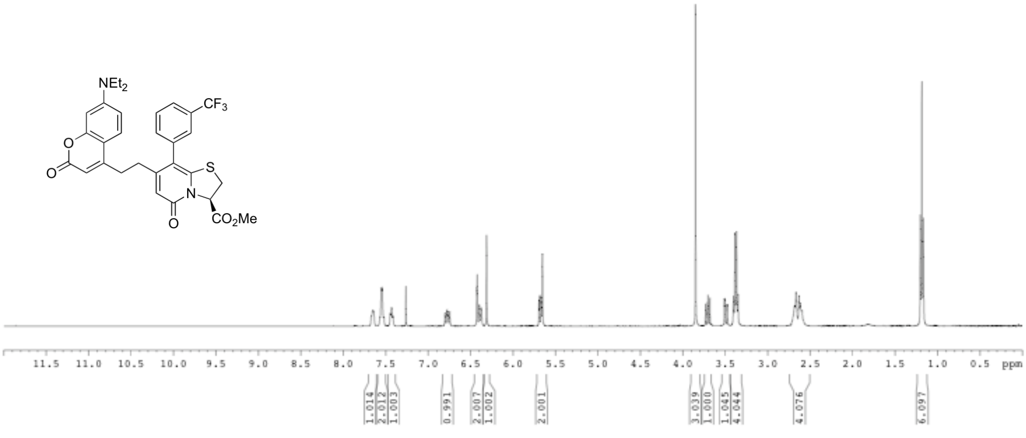

^{13}C-NMR spectrum of compound **15c**

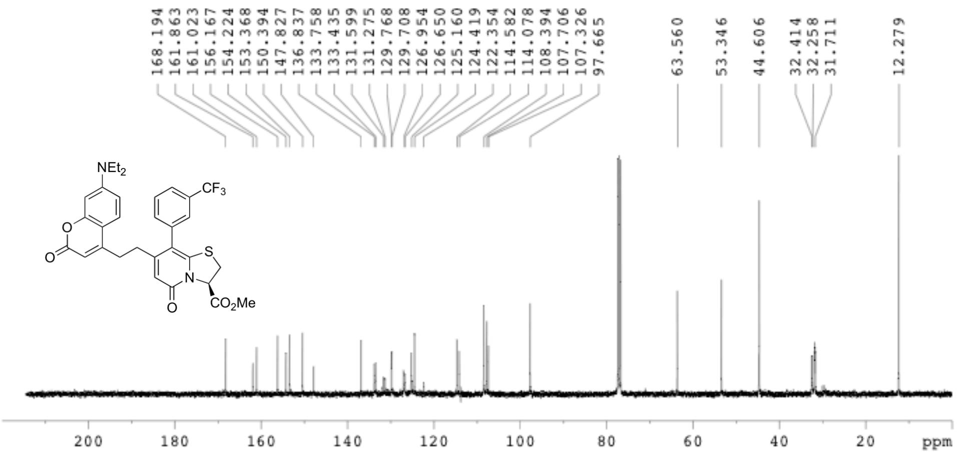

^{1}H-NMR spectrum of compound **15d**

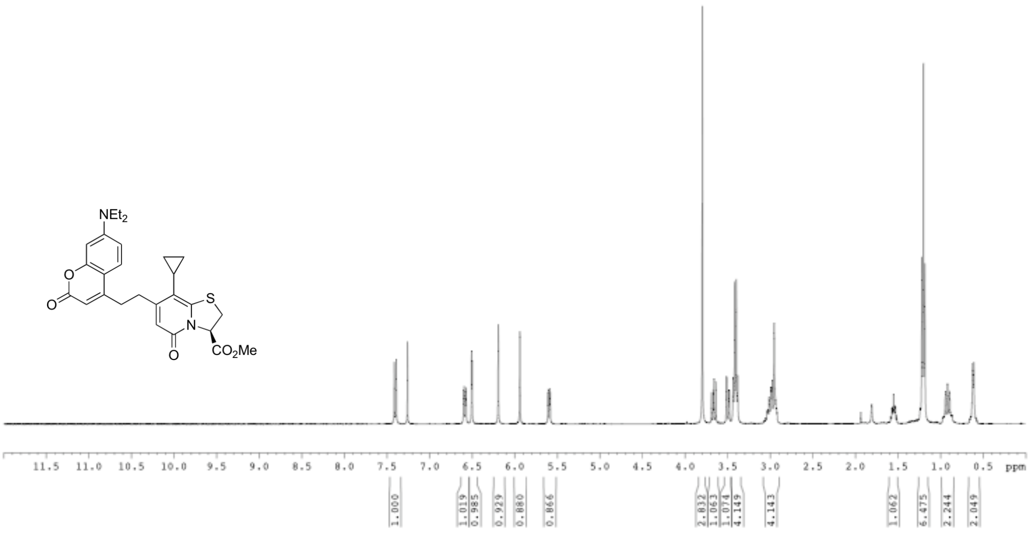

^{13}C-NMR spectrum of compound **15d**

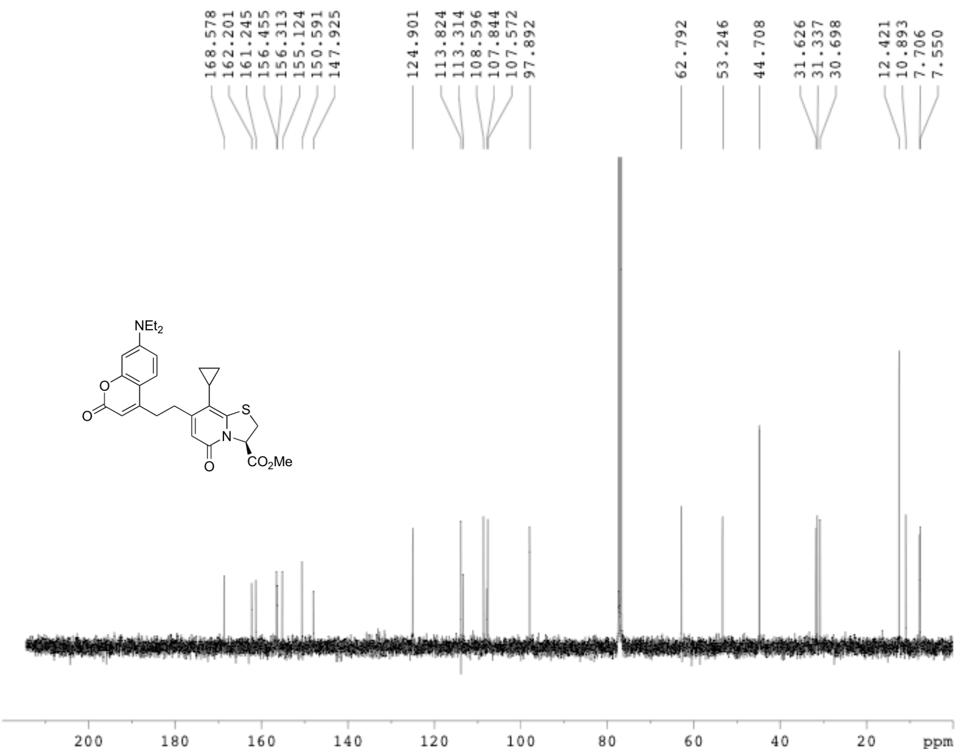

^{1}H-NMR spectrum of compound **16a**

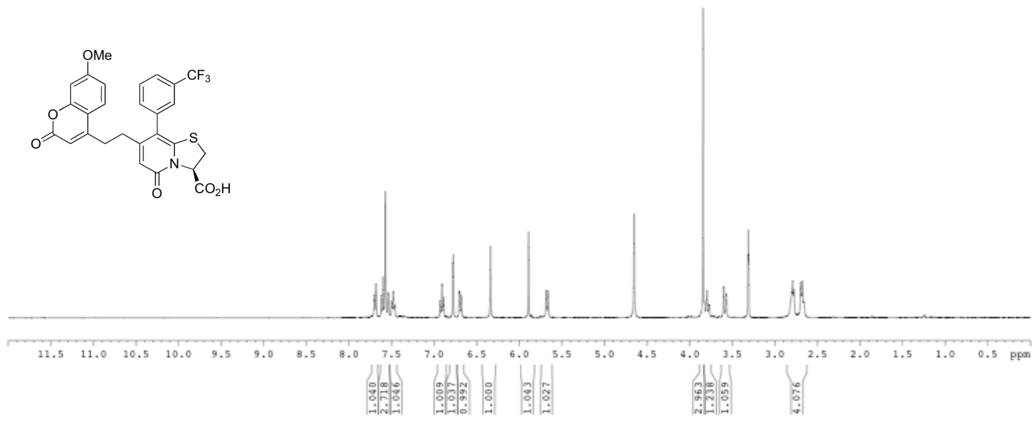

^{13}C-NMR spectrum of compound **16a**

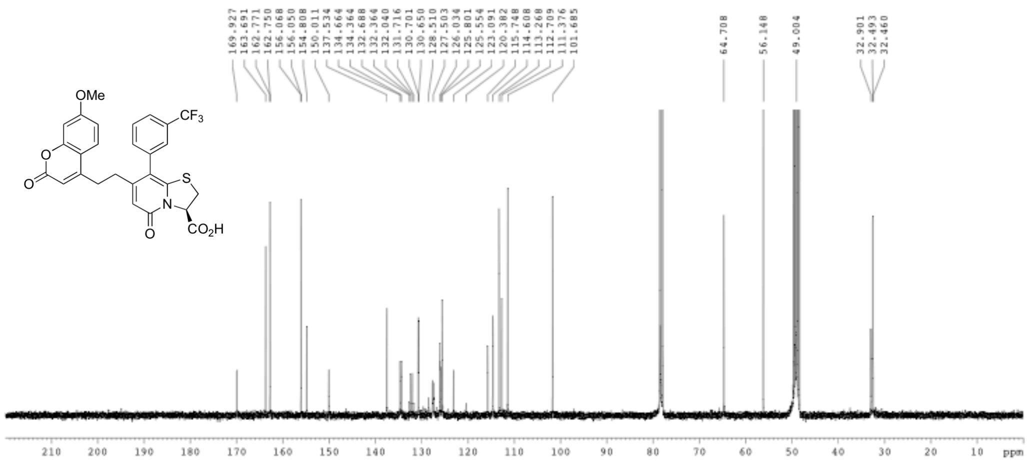

^{1}H-NMR spectrum of compound **16b**

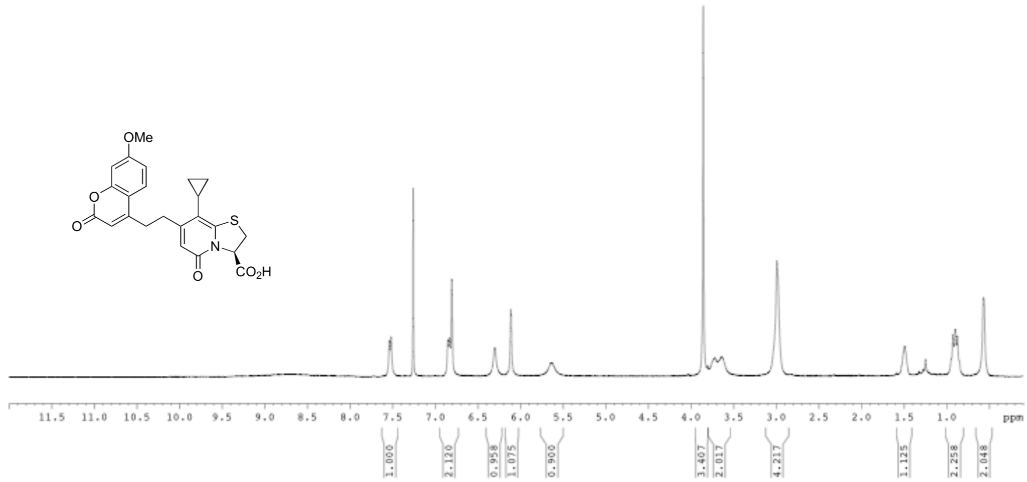

^{13}C-NMR spectrum of compound **16b**

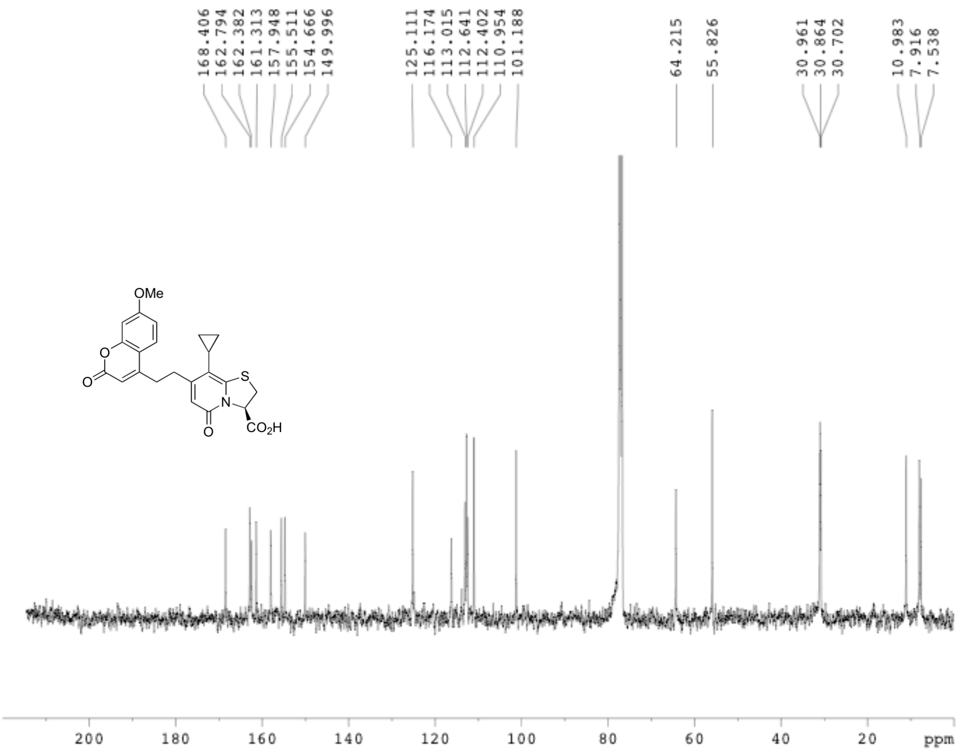

^{1}H-NMR spectrum of compound **16c**

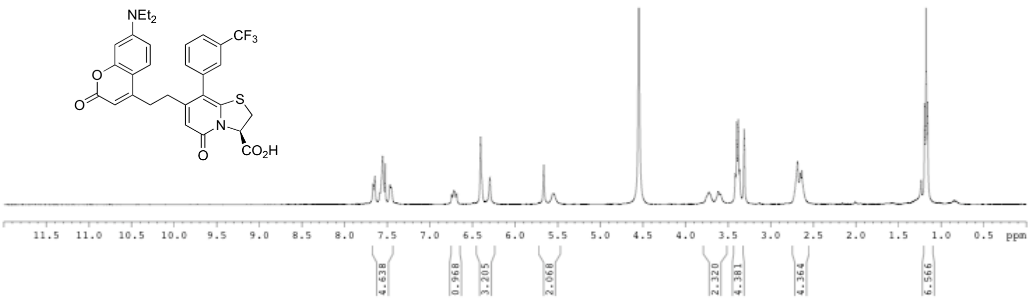

^{13}C-NMR spectrum of compound **16c**

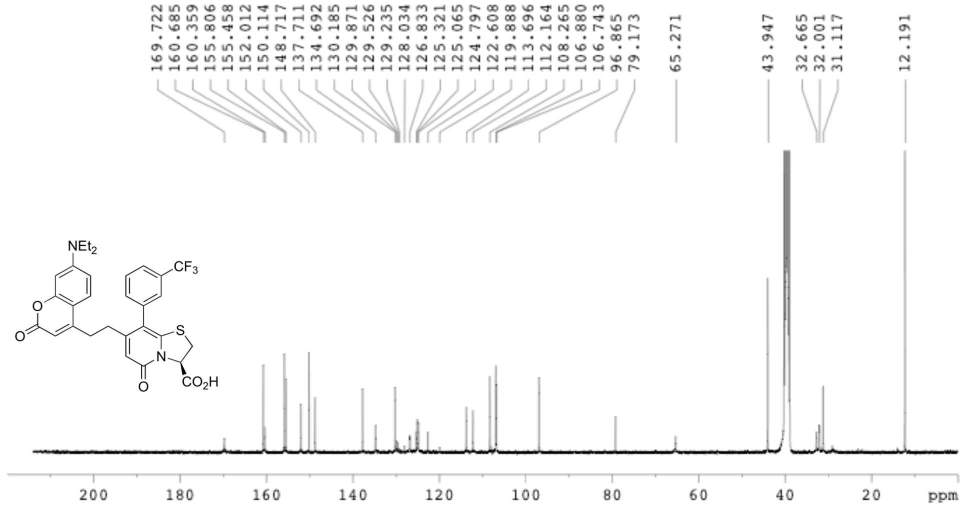

^{1}H-NMR spectrum of compound **16d**

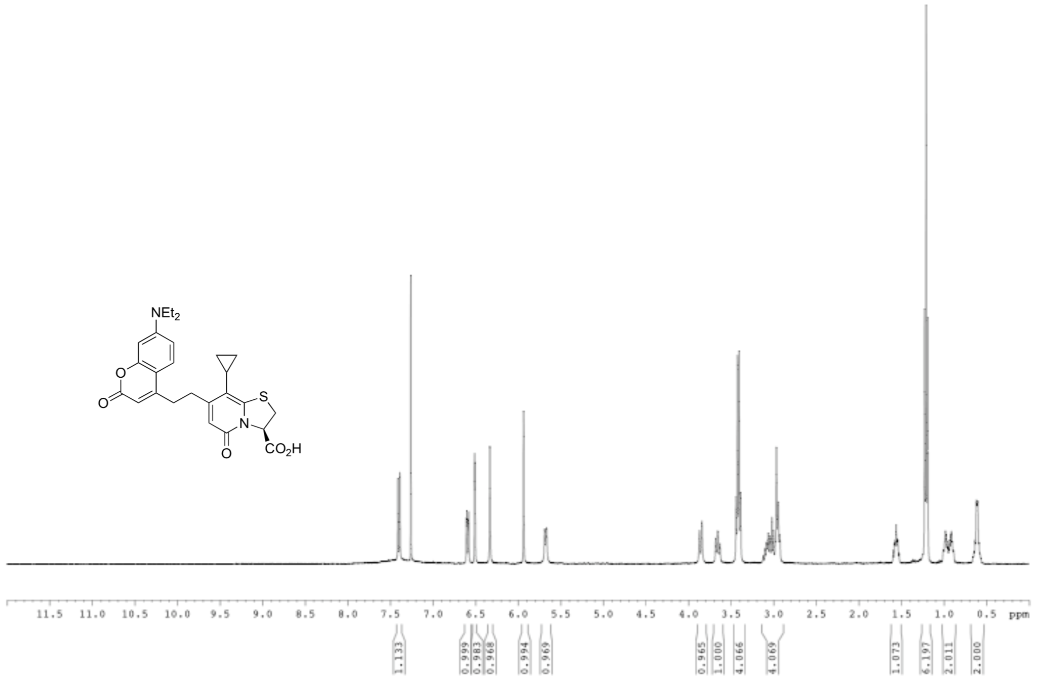

^{13}C-NMR spectrum of compound **16d**

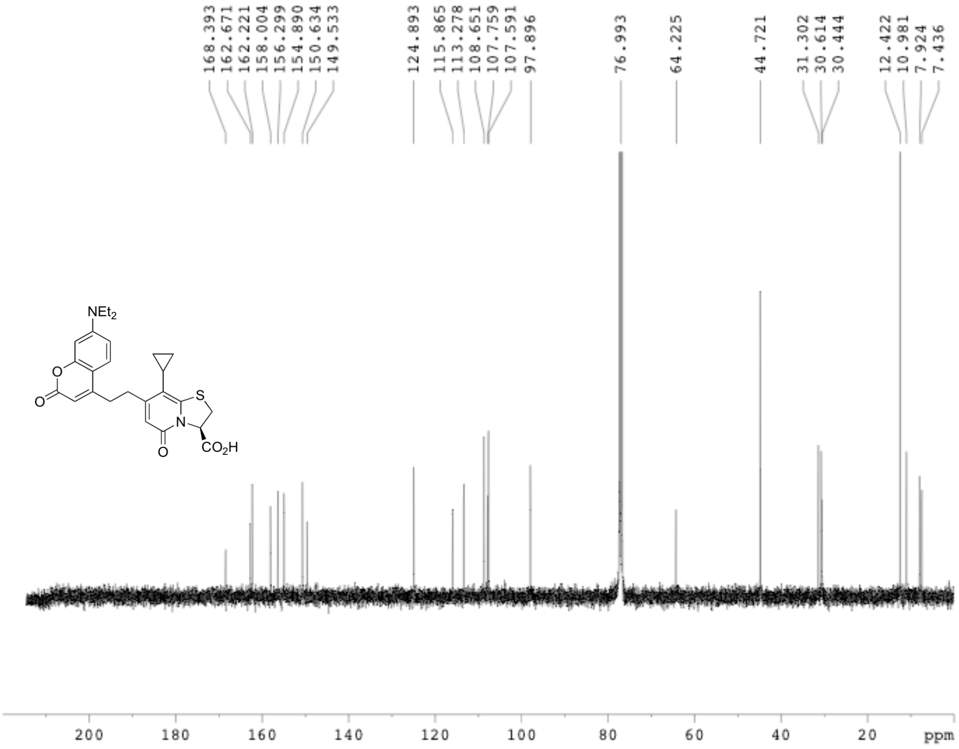

^{1}H-NMR spectrum of compound **17**

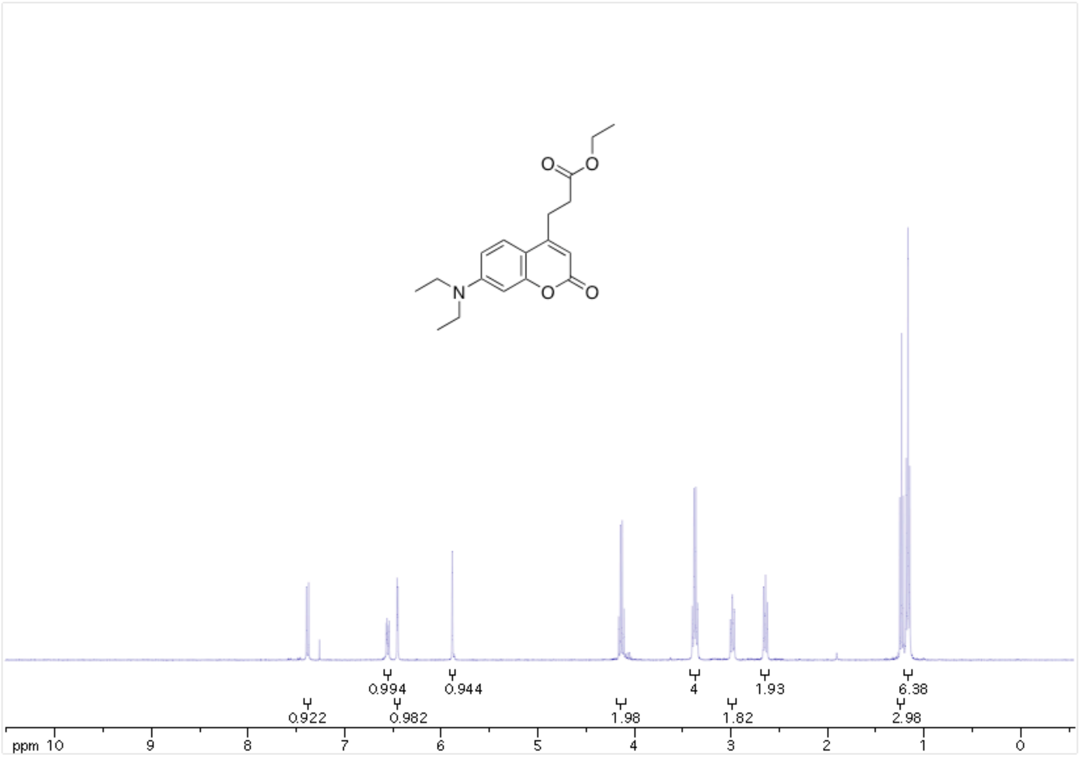

^{13}C-NMR spectrum of compound **17**

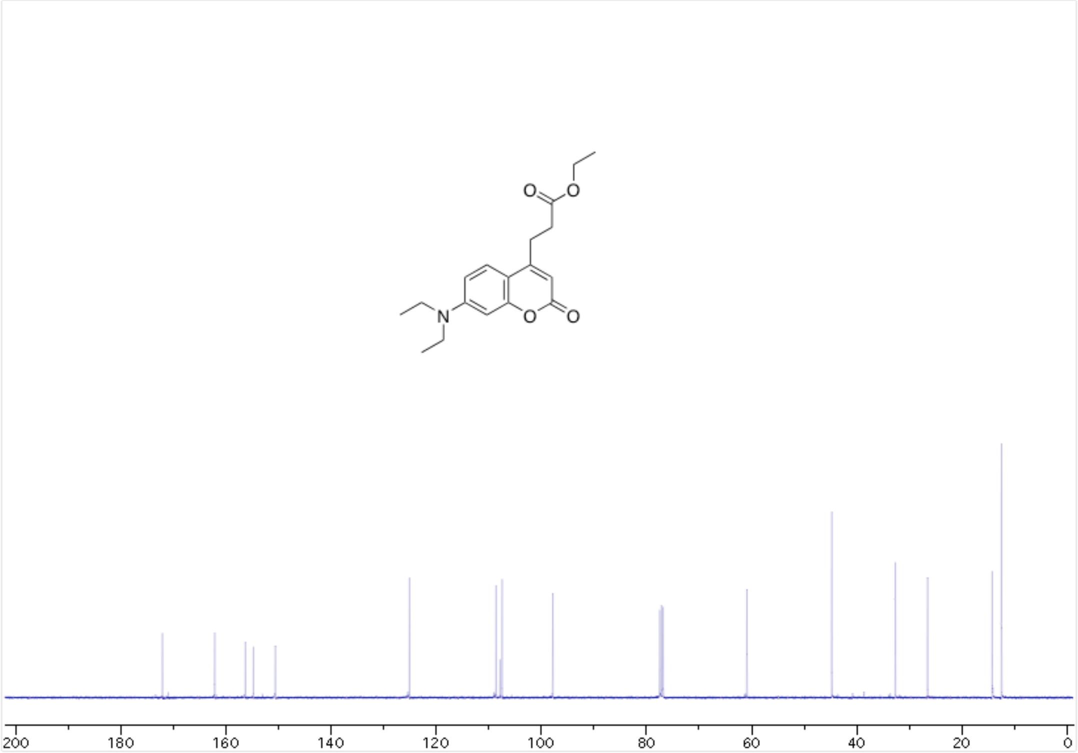

^{1}H-NMR spectrum of compound **19**

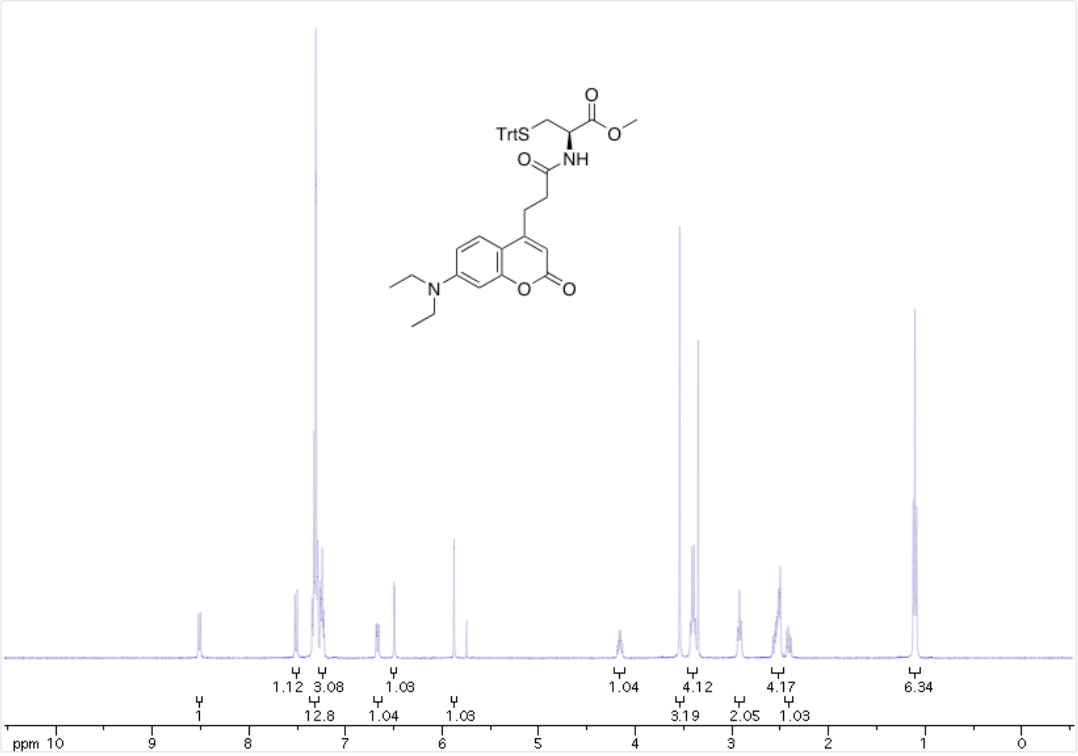

^{13}C-NMR spectrum of compound **19**

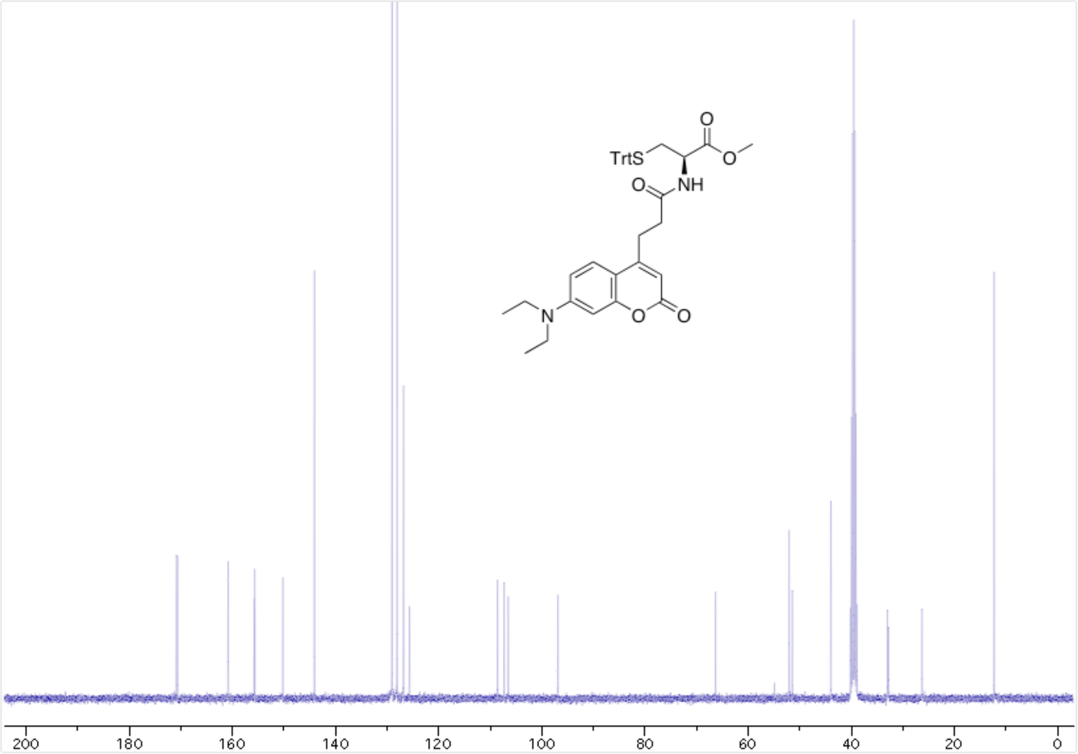

^{1}H-NMR spectrum of compound **20**

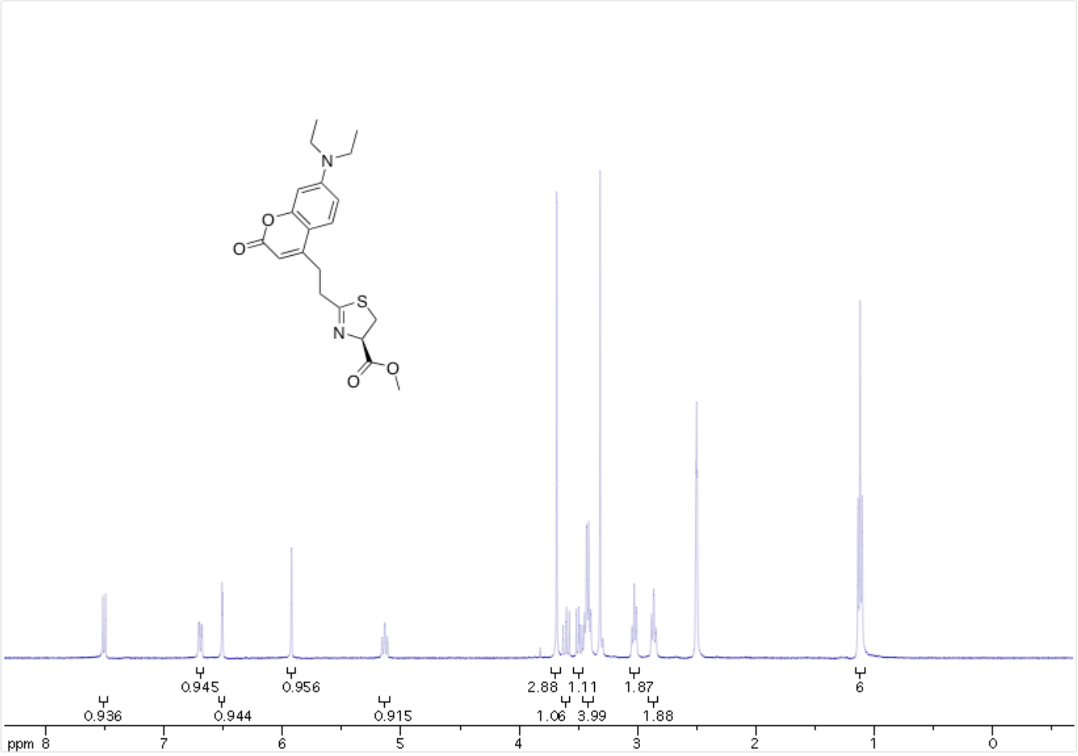

^{13}C-NMR spectrum of compound **20**

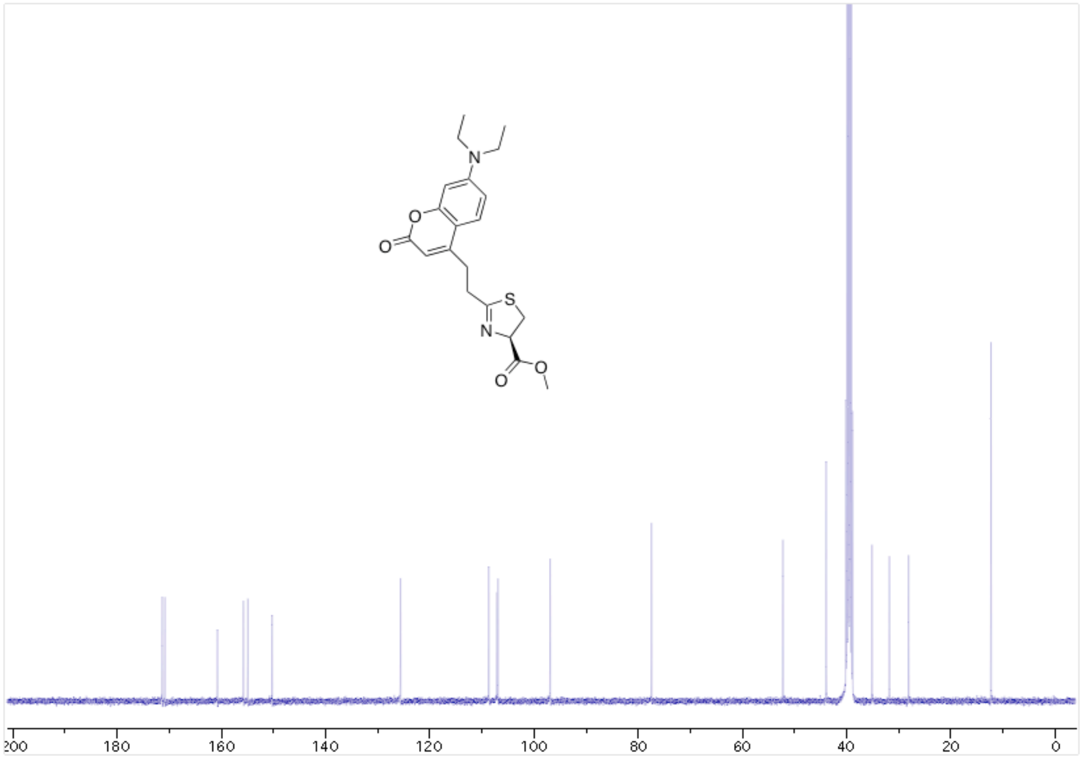

^{1}H-NMR spectrum of compound **21**

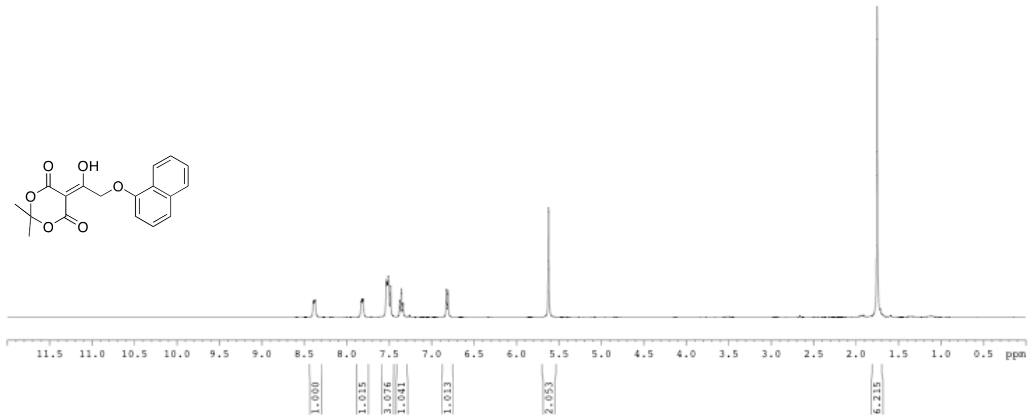

^{13}C-NMR spectrum of compound **21**

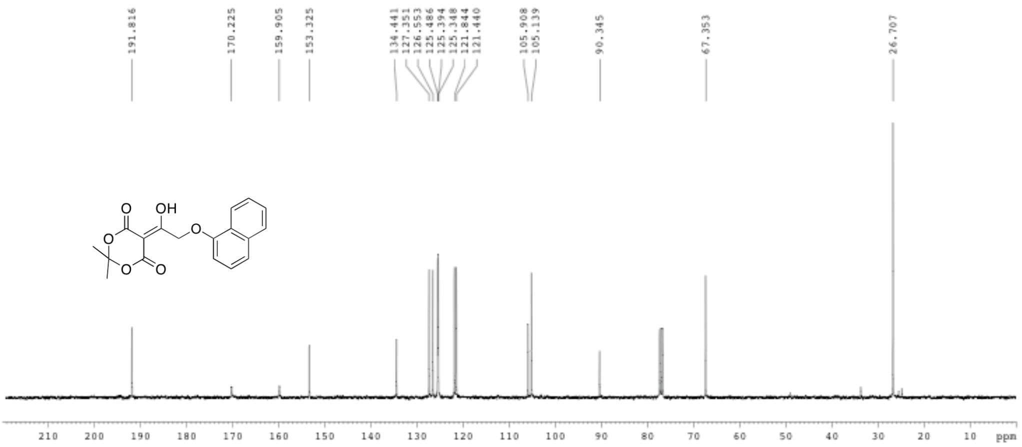

^{1}H-NMR spectrum of compound **22**

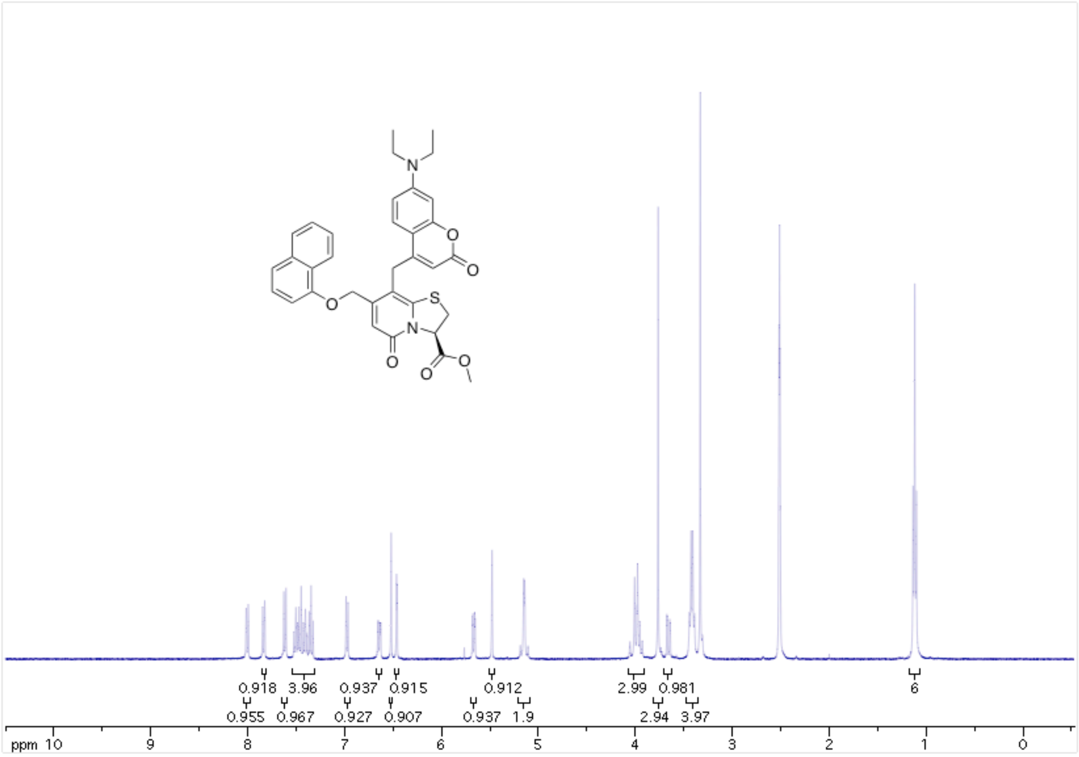

^{13}C-NMR spectrum of compound **22**

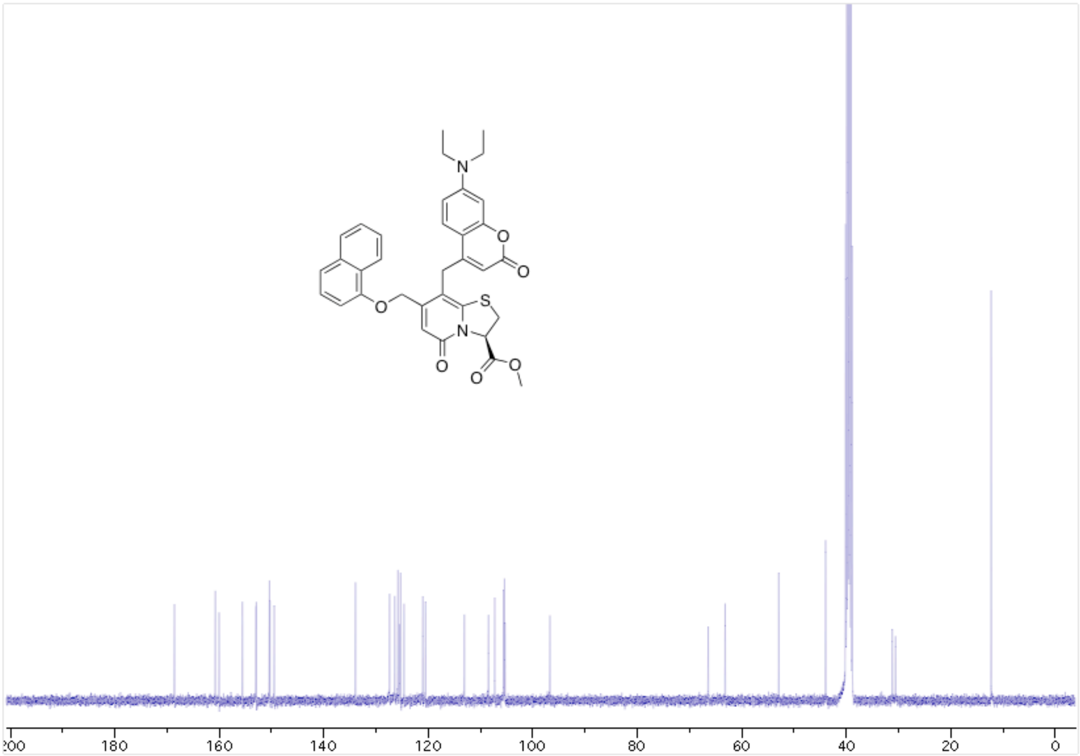

^{1}H-NMR spectrum of compound **23**

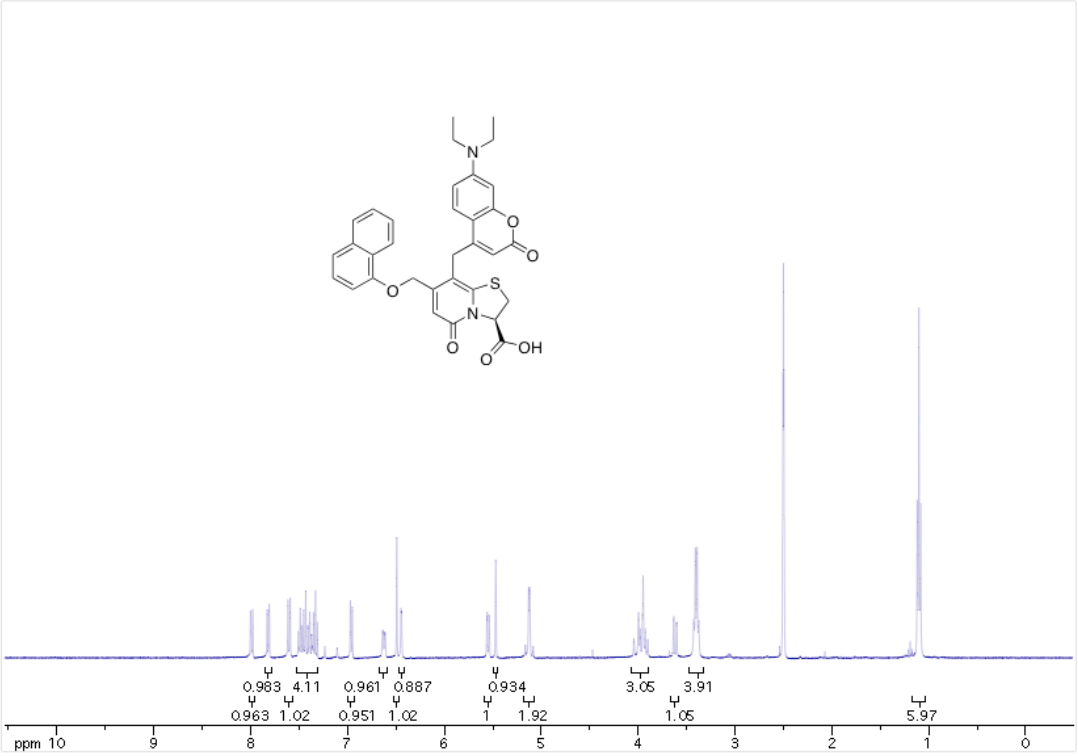

^{13}C-NMR spectrum of compound **23**

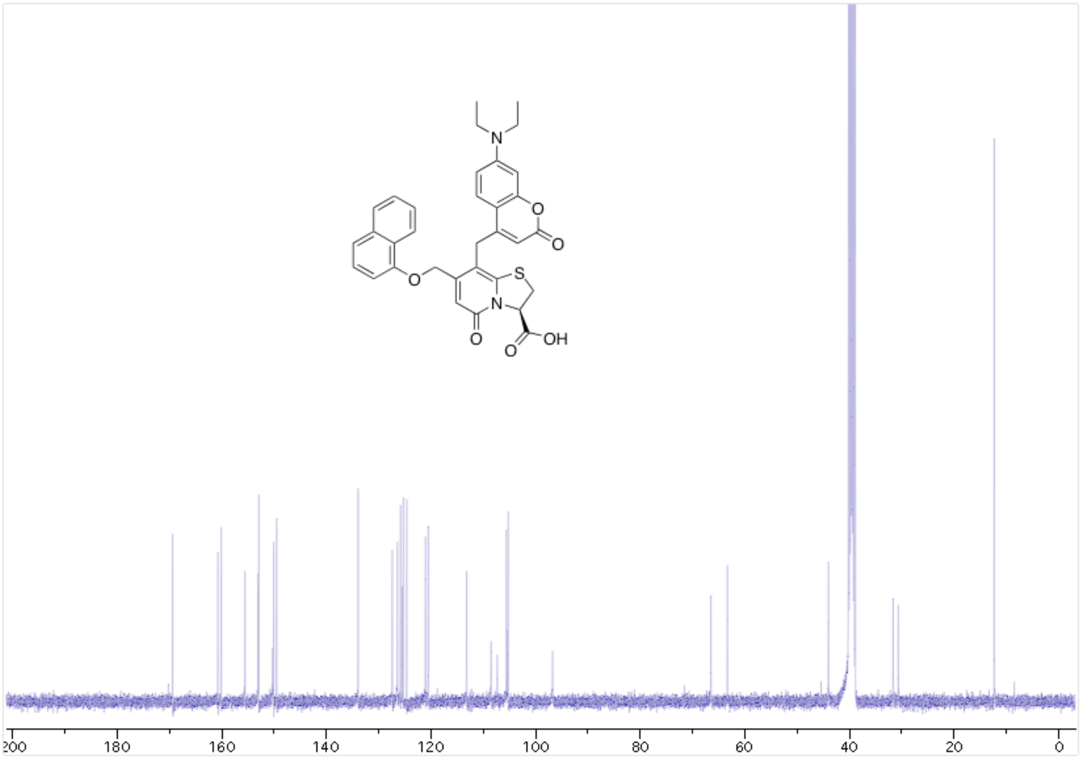

^{1}H-NMR spectrum of compound **24**

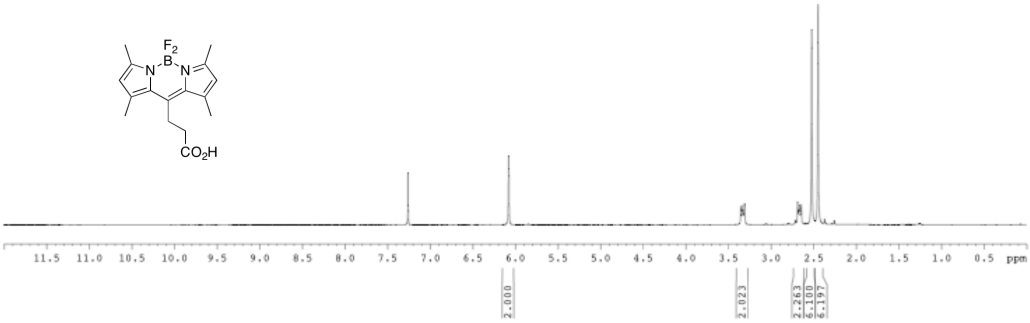

^{13}C-NMR spectrum of compound **24**

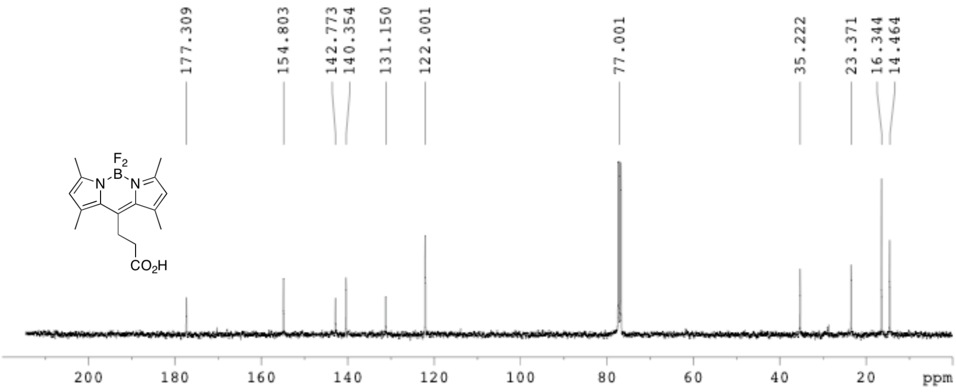

^{1}H-NMR spectrum of compound **25**

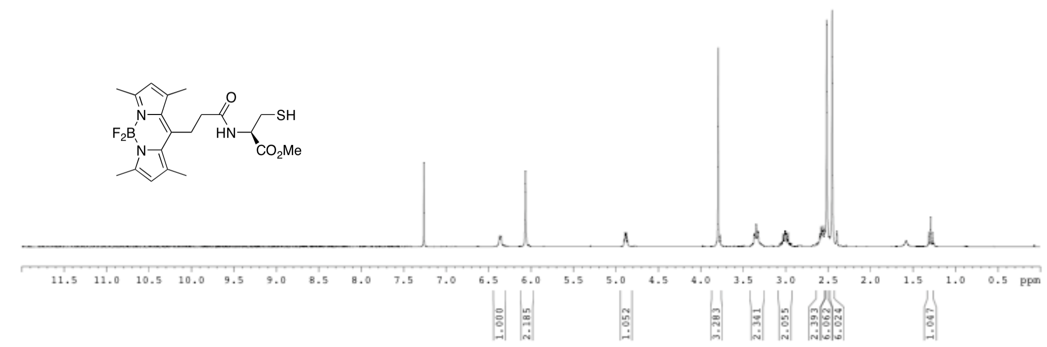

^{13}C-NMR spectrum of compound **25**

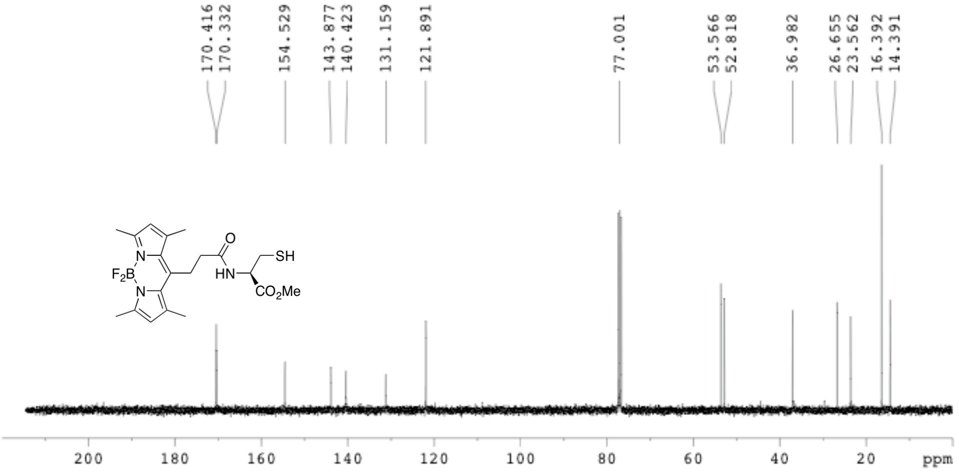

^{1}H-NMR spectrum of compound **26**

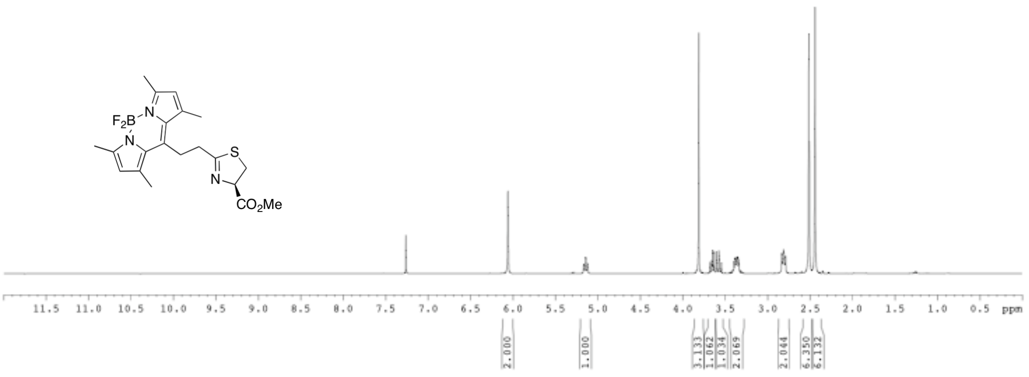

^{13}C-NMR spectrum of compound **26**

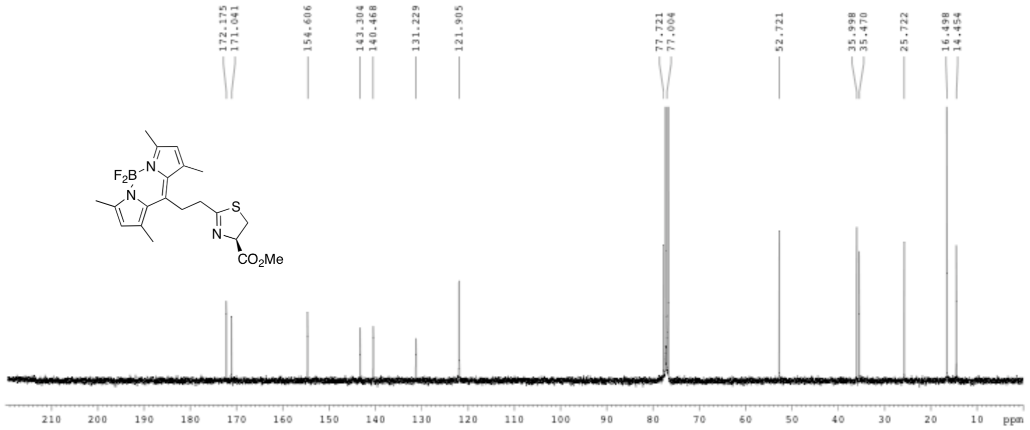

^{1}H-NMR spectrum of compound **27**

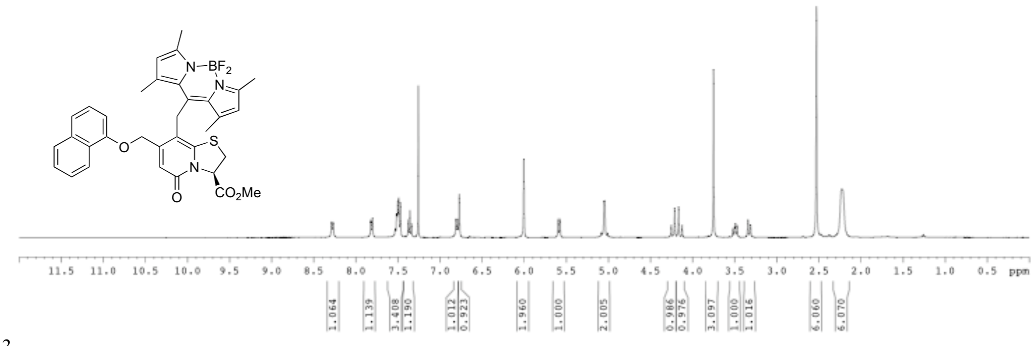

^{13}C-NMR spectrum of compound **27**

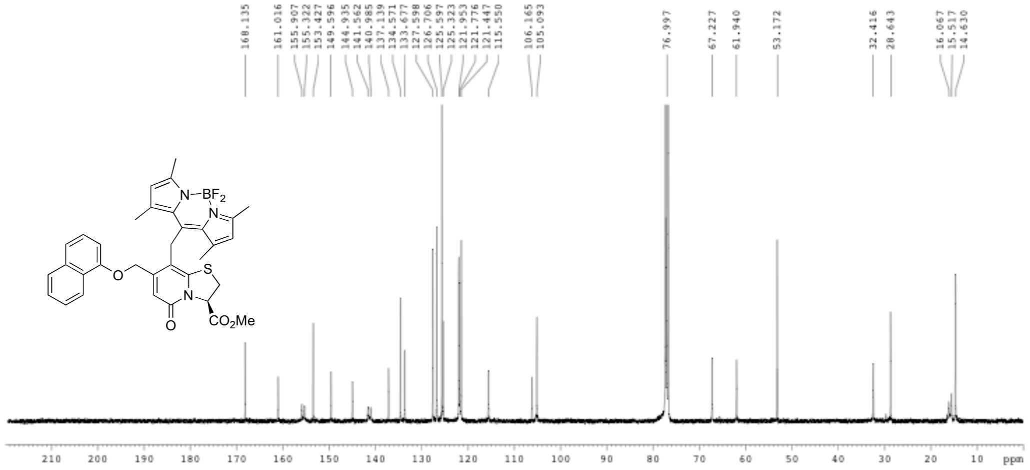

^{1}H-NMR spectrum of compound **28**

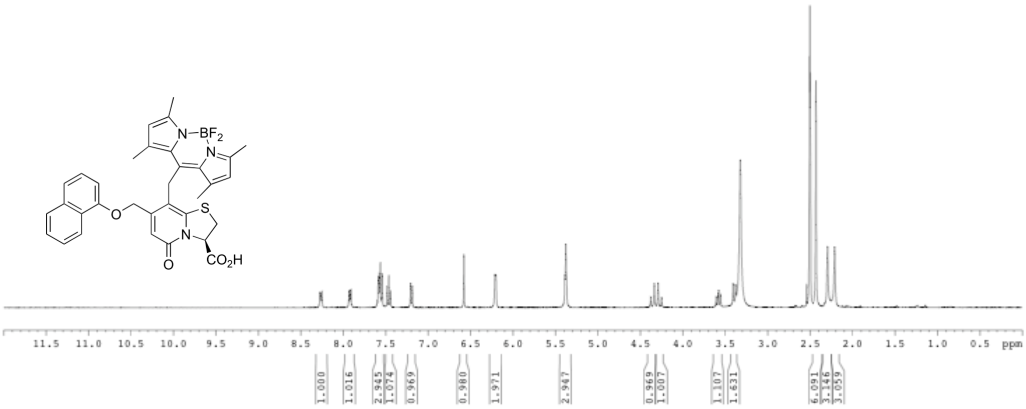

^{13}C-NMR spectrum of compound **28**

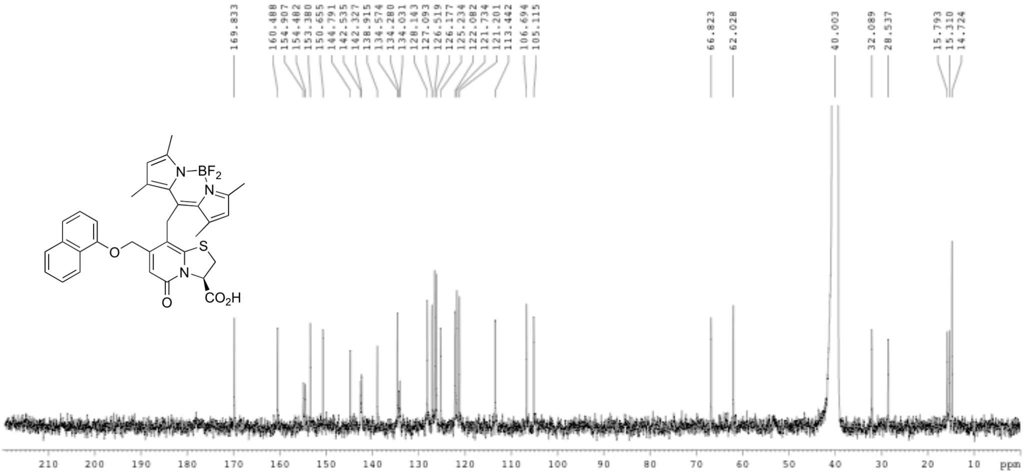

^{1}H-NMR spectrum of compound **30**

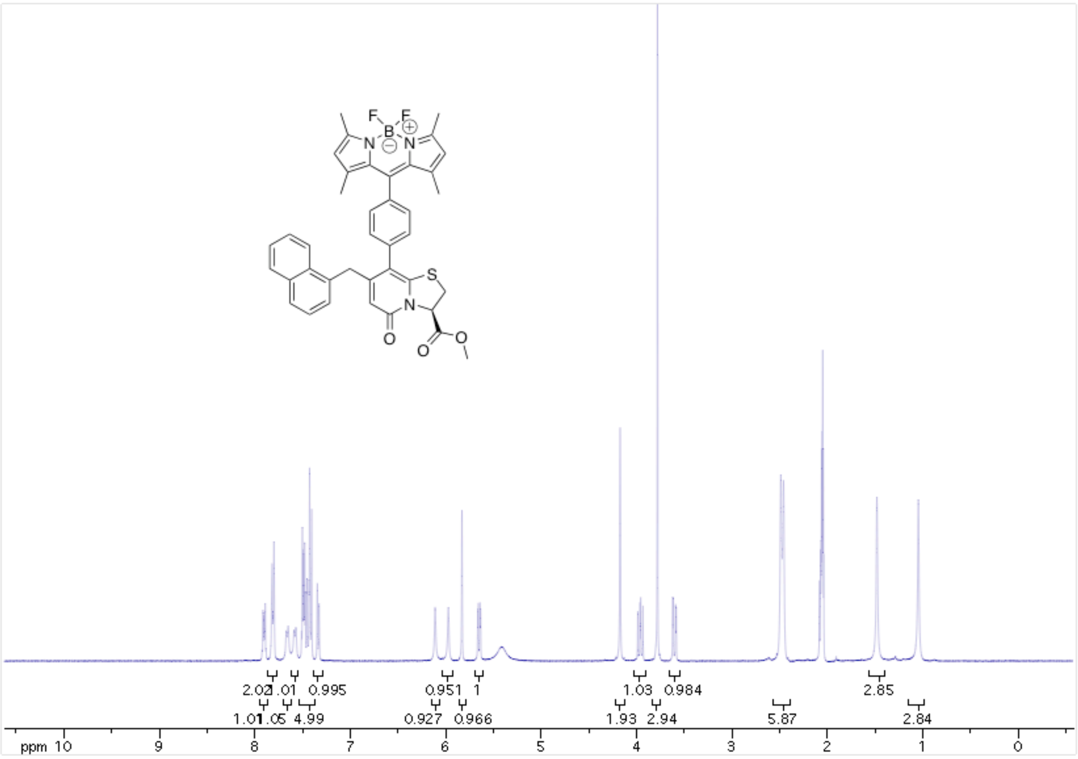

^{13}C-NMR spectrum of compound **30**

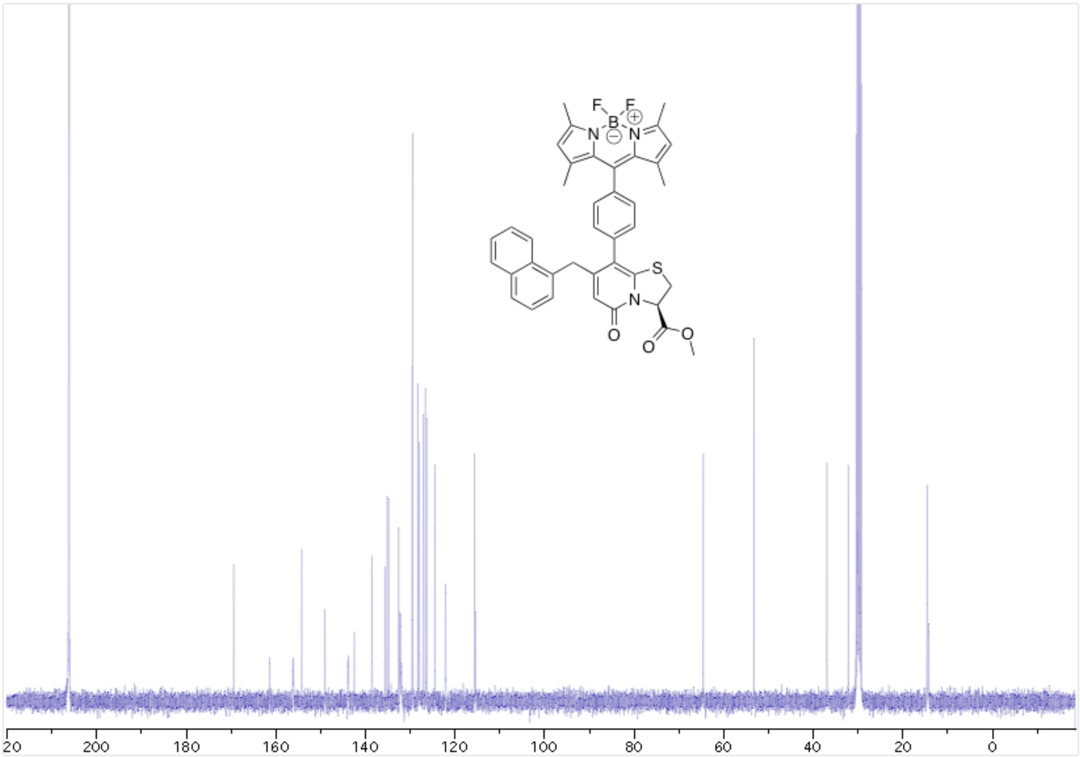

^{1}H-NMR spectrum of compound **31**

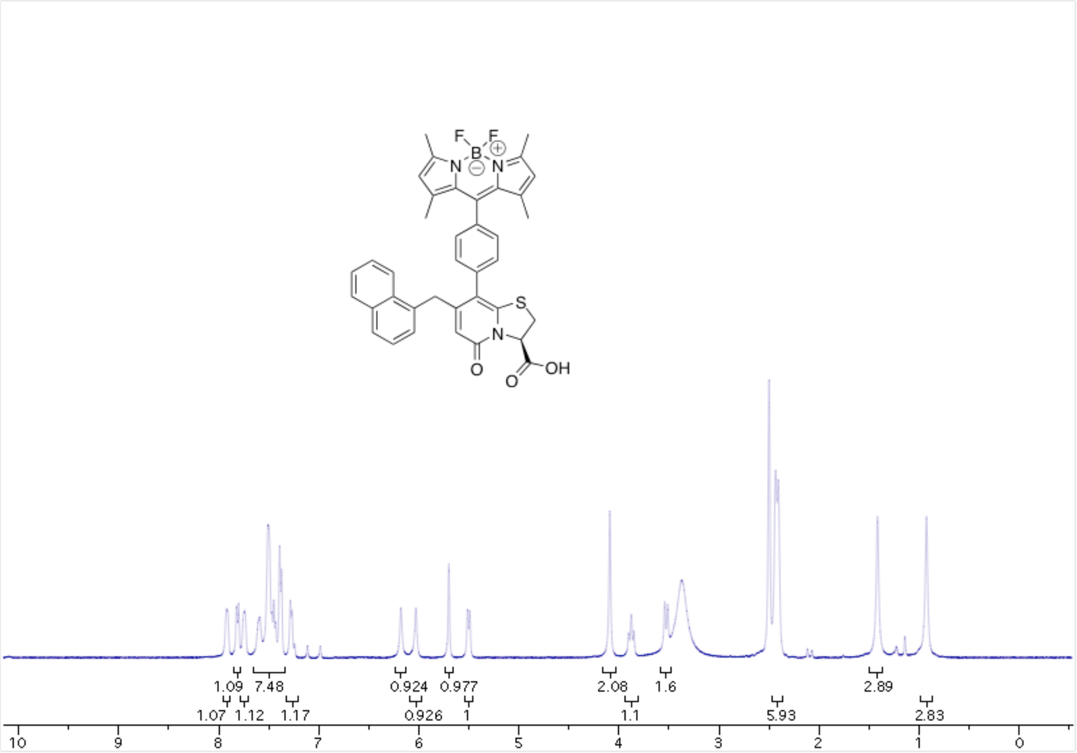

^{13}C-NMR spectrum of compound **31**

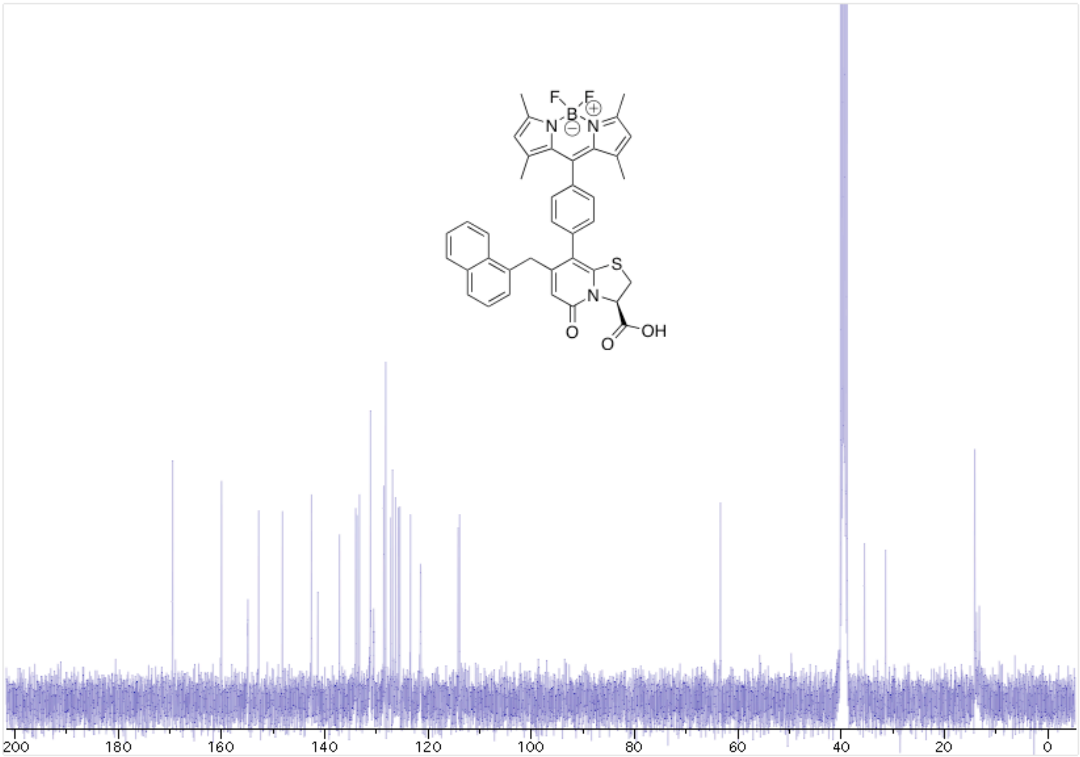

^{1}H-NMR spectrum of compound **32**

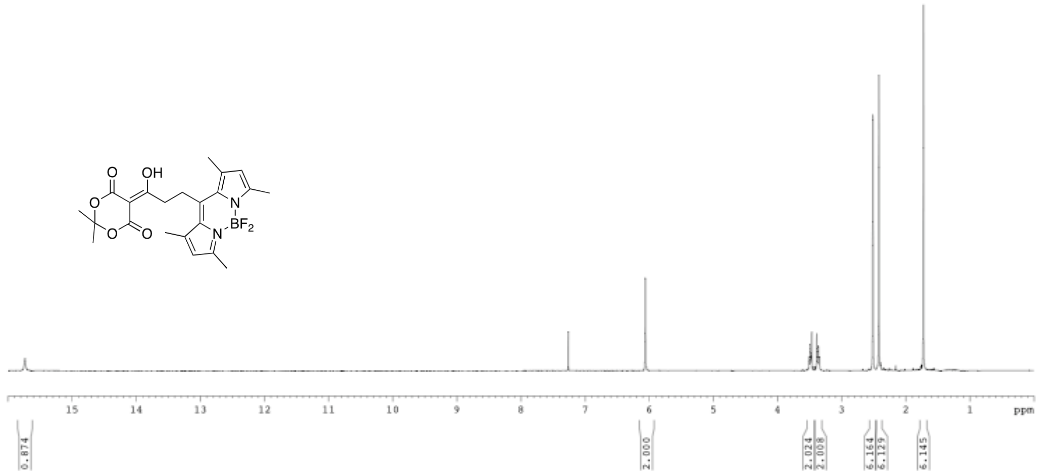

^{13}C-NMR spectrum of compound **32**

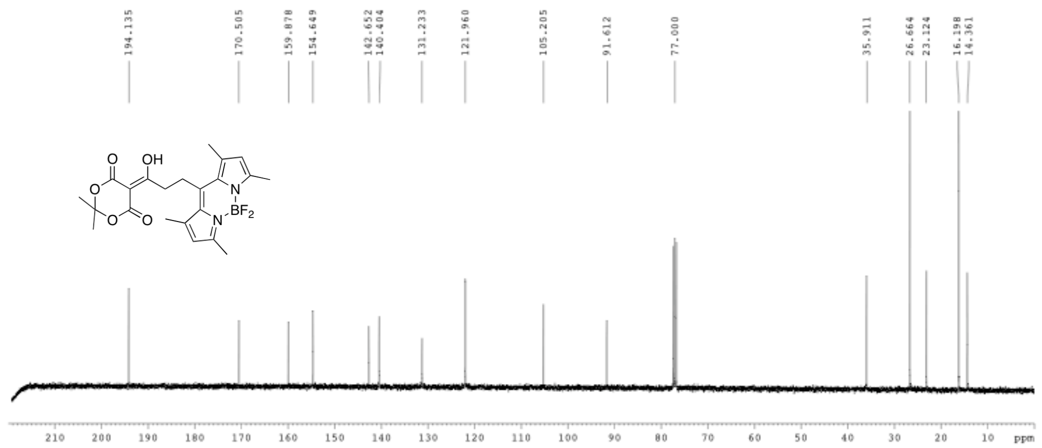

^{1}H-NMR spectrum of compound **33**

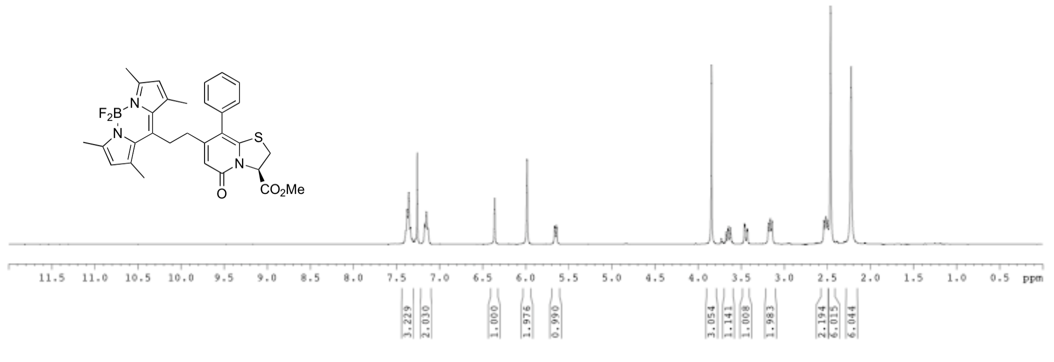

^{13}C-NMR spectrum of compound **33**

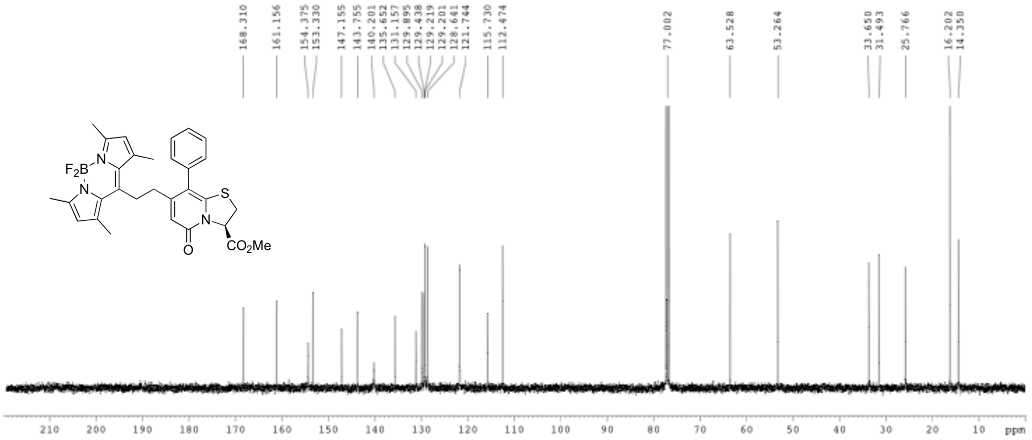

^{1}H-NMR spectrum of compound **34**

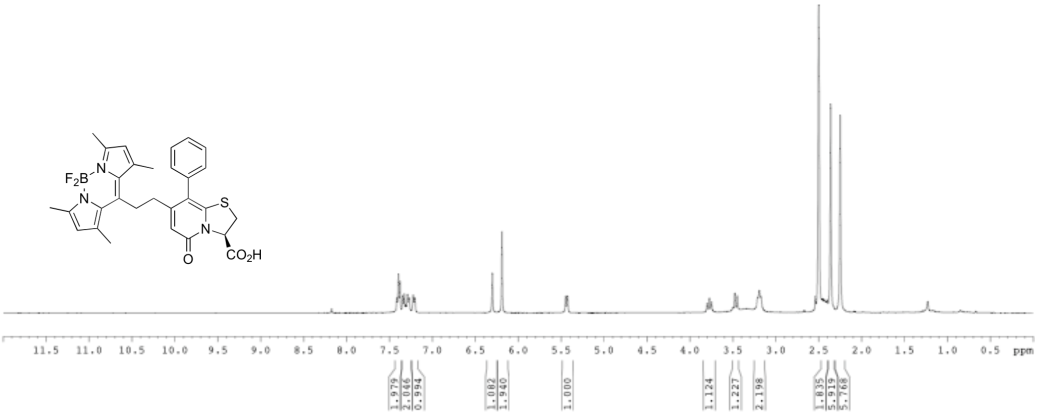

^{13}C-NMR spectrum of compound **34**

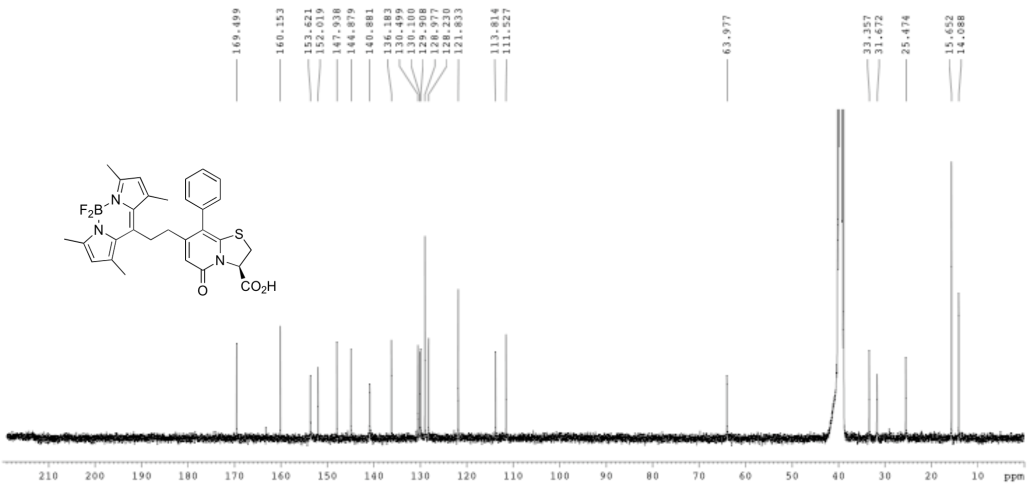

^{1}H-NMR spectrum of compound **36**

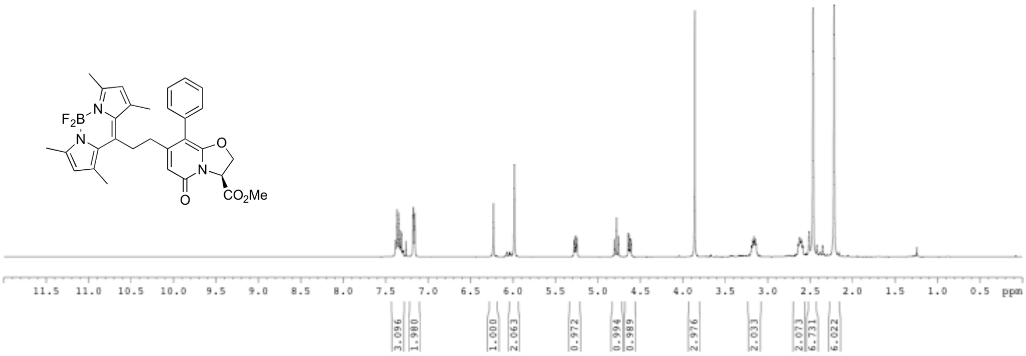

^{13}C-NMR spectrum of compound **36**

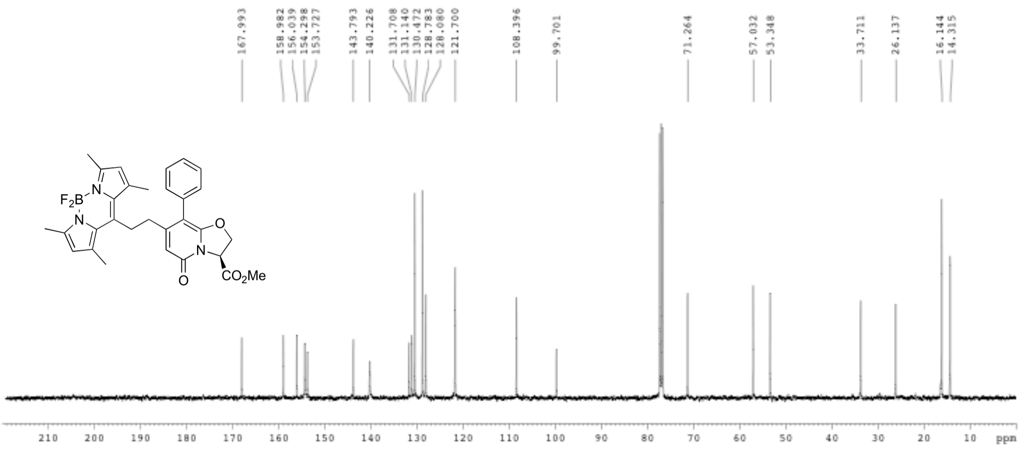

^{1}H-NMR spectrum of compound **37**

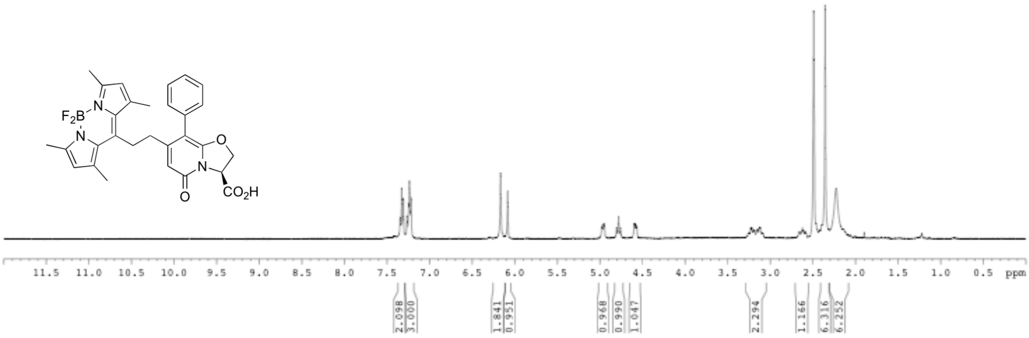

^{13}C-NMR spectrum of compound **37**

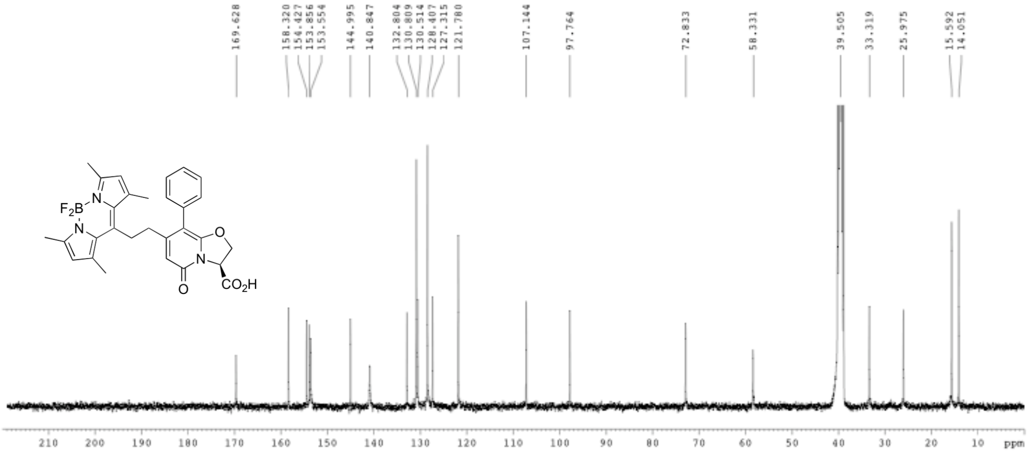

^{1}H-NMR spectrum of compound **38**

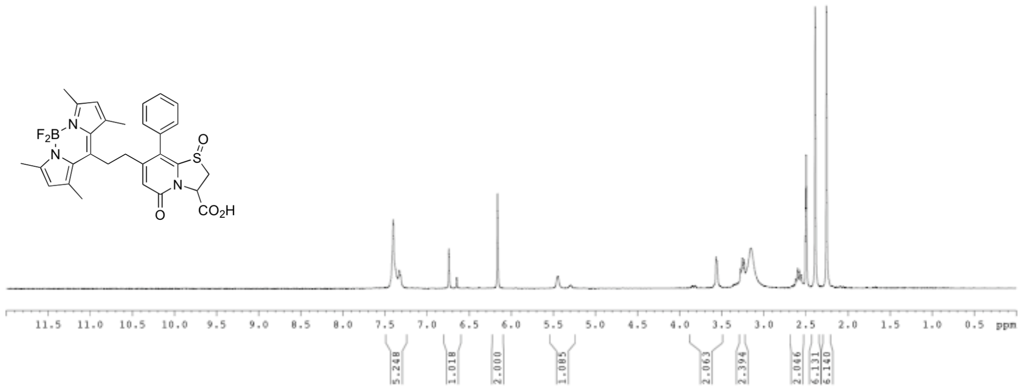

COSEY spectrum of compound **38**

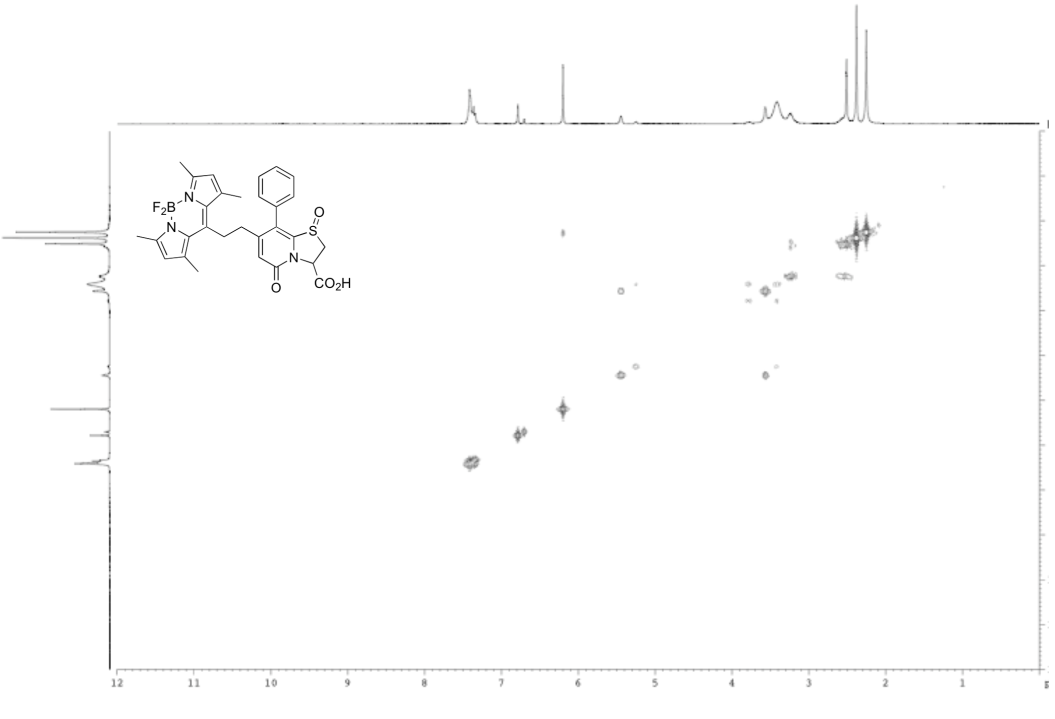

^{13}C-NMR spectrum of compound **38**

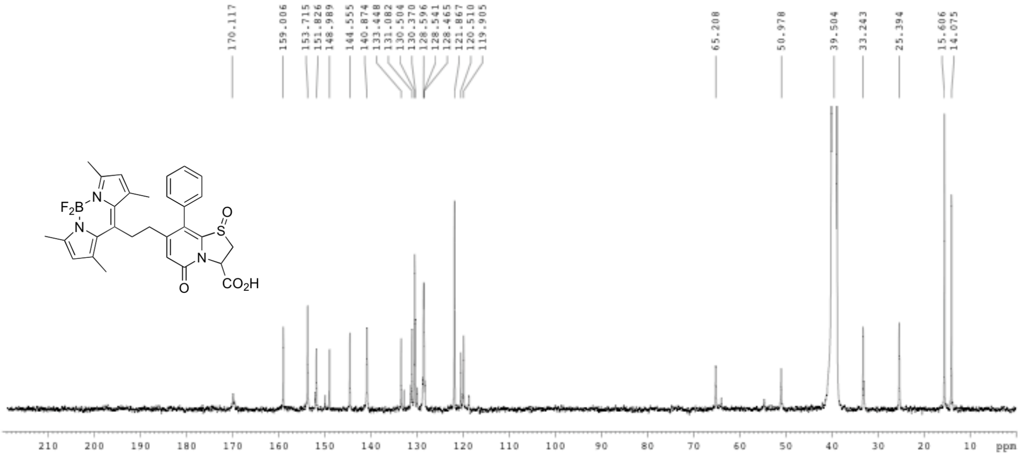

References

(1) Chorell, E.; Bengtsson, C.; Banchelin, T. S.; Das, P.; Uvell, H.; Sinha, A. K.; Pinkner, J. S.; Hultgren, S. J.; Almqvist, F. *E. J. Med. Chem.* **2011**, *46*, 1103-1116.
(2) Emtenäs, H.; Alderin, L.; Almqvist, F. *J. Org. Chem.* **2001**, *66*, 6756-6761.
(3) Pemberton, N.; Pinkner, J. S.; Edvinsson, S.; Hultgren, S. J.; Almqvist, F. *Tetrahedron* **2008**, *64*, 9368-9376.
(4) Mattsson, S.; Dahlstrom, M.; Karlsson, S. *Tetrahedron Lett.* **2007**, *48*, 2497-2499.
